# Metabolomics Insights into Chemical Convergence in *Xanthomonas perforans* and Metabolic Changes Following Treatment with the Small Molecule Carvacrol

**DOI:** 10.3390/metabo11120879

**Published:** 2021-12-16

**Authors:** Mustafa Ojonuba Jibrin, Qingchun Liu, Joy Guingab-Cagmat, Jeffrey B. Jones, Timothy J. Garrett, Shouan Zhang

**Affiliations:** 1 Tropical Research and Education Center, IFAS, University of Florida, Homestead, FL 33031, USA; jibrinmo@ufl.edu (M.O.J.); q.liu1@ufl.edu (Q.L.); 2 Department of Crop Protection, Ahmadu Bello University, Zaria 810103, Nigeria; 3 Department of Pathology, Immunology, and Laboratory Medicine, University of Florida, Gainesville, FL 32610, USA; joydg@ufl.edu (J.G.-C.); tgarrett@ufl.edu (T.J.G.); 4 Plant Pathology Department, University of Florida, Gainesville, FL 32611, USA; jbjones@ufl.edu

**Keywords:** *Xanthomonas*, *Xanthomonas perforans*, carvacrol, pathways, chemicals, metabolites

## Abstract

Microbes are natural chemical factories and their metabolome comprise diverse arrays of chemicals. The genus *Xanthomonas* comprises some of the most important plant pathogens causing devastating yield losses globally and previous studies suggested that species in the genus are untapped chemical minefields. In this study, we applied an untargeted metabolomics approach to study the metabolome of a globally spread important xanthomonad, *X. perforans*. The pathogen is difficult to manage, but recent studies suggest that the small molecule carvacrol was efficient in disease control. Bacterial strains were treated with carvacrol, and samples were taken at time intervals (1 and 6 h). An untreated control was also included. There were five replicates for each sample and samples were prepared for metabolomics profiling using the standard procedure. Metabolomics profiling was carried out using a thermo Q-Exactive orbitrap mass spectrometer with Dionex ultra high-performance liquid chromatography (UHPLC) and an autosampler. Annotation of significant metabolites using the Metabolomics Standards Initiative level 2 identified an array of novel metabolites that were previously not reported in *Xanthomonas perforans*. These metabolites include methoxybrassinin and cyclobrassinone, which are known metabolites of brassicas; sarmentosin, a metabolite of the *Passiflora-*heliconiine butterfly system; and monatin, a naturally occurring sweetener found in *Sclerochiton ilicifolius*. To our knowledge, this is the first report of these metabolites in a microbial system. Other significant metabolites previously identified in non-*Xanthomonas* systems but reported in this study include maculosin; piperidine; β-carboline alkaloids, such as harman and derivatives; and several important medically relevant metabolites, such as valsartan, metharbital, pirbuterol, and ozagrel. This finding is consistent with convergent evolution found in reported biological systems. Analyses of the effect of carvacrol in time-series and associated pathways suggest that carvacrol has a global effect on the metabolome of *X. perforans,* showing marked changes in metabolites that are critical in energy biosynthesis and degradation pathways, amino acid pathways, nucleic acid pathways, as well as the newly identified metabolites whose pathways are unknown. This study provides the first insight into the *X. perforans* metabolome and additionally lays a metabolomics-guided foundation for characterization of novel metabolites and pathways in xanthomonad systems.

## 1. Introduction

Metabolomics aims to uncover the totality of molecules that are present in a system, including biofluids, living cells, and environmental mixtures [1,2,3,4]. In life sciences, untargeted metabolomics enables the characterization of the totality of small molecules in a specimen, leading to the discovery of bioactive metabolites that discriminate between phenotypes [2,3]. Untargeted metabolomics, therefore, helps in generating hypotheses, and such data may be used to identify new metabolic pathways which form the background for further studies [5,6]. Analytical methods for mass spectrometry-based metabolomics profiling include gas chromatography-mass spectrometry (GC-MS), liquid chromatography-mass spectrometry (LC-MS), and capillary electrophoresis-mass spectrometry (CE-MS) [7]. Metabolite identification is, however, often a significant bottleneck in subsequent metabolomics analysis because nontargeted metabolomics generates a large amount of data for the global metabolites present in a sample [8]. Annotation of unknown metabolites from nontargeted metabolomics is an active area of research and often leads to the discovery of novel metabolites that are important in pathways of biological systems.

Over the years, the identification of similar metabolites in different biological systems has spurred interest in convergent evolution of such chemicals [9,10]. Independent evolution and coevolution of similar phenotypes or traits in distinct biological systems defines convergent evolution [10]. Coevolutionary arms race has also resulted in the identification of similar chemicals or compounds in a pathogen and its host, a predator and its prey, and sometimes, different plant and animal systems evolve different pathways in the synthesis of the same metabolite [9,10,11,12,13,14]. Convergent evolution in chemical ecology has also resulted in the synthesis of the same compound in different lineages utilizing unrelated enzymes or the same pathway evolving independently in different biological systems or different chemicals having the same functions in different biological systems [9,13]. A famous example is caffeine, which has been reported in different plants with varying levels of retention, functions, and independent evolution of biosynthetic pathways in *Coffea* (coffee) and *Camellia* (tea) [15,16]. Similarly, bacteria have evolved widely varying but functionally similar siderophores for the purpose of iron scavenging and transport across cell membranes (reviewed in [17]). The identification of similar phenotypes in different genetic lineages improves insights in understanding chemical ecology, coevolutionary, and other pathoadaptation dynamics in different species.

Plants and plant-associated microbes are reservoirs of many important metabolites that function in plant, microbe, animal, and human metabolic pathways [18,19]. The plant-microbe systems harbor a large set of metabolites, with a plant system alone having more than 30,000 phytochemicals [20,21,22,23]. Plants coexist with myriads of microbes, the dynamics of which are shaped by a complex pattern of plant–microbe interactions [19,24]. While many microbes may be harmless, the few that cause diseases produce small molecules that help the microbes to adapt within the plant environment [18]. Some of these chemicals include fungal polyketides and organic acids, alkaloids and terpenes, bacterial siderophores, toxins, lipopeptides, and non-ribosomal peptides (NRPs) [18,25]. Plant-associated microbes can deliver substances, such as toxins, effector proteins, and indole-3-acetic acid, into plants that direct plant development or cause plant diseases [26,27,28]. Microbial virulence factors contribute to the establishment of a disease relationship between a microbe and a ‘host’ plant [29,30]. Methodical identification of these molecules in species across several microbial genera has advanced the understanding of phytochemicals, the chemicals involved in pathogen–pathogen and host–pathogen interactions [30,31,32,33].

Among bacterial plant pathogens, species within the genus *Xanthomonas* cause some of the most devastating plant diseases worldwide [34,35]. Successful infection and multiplication in host tissues often depend on virulence factors, such as adhesins, degradative enzymes, polysaccharides, and lipopolysaccharides (LPSs), that are secreted or delivered to the outer membrane through diverse secretory systems [36]. It is largely held that *Xanthomonas* spp. possess a large yet untapped reservoir of bioactive small molecules and peptides [37]. Species within this genus produce important arrays of chemicals, including the commercially and widely used xanthan, a common additive in the food industry [38]. The small molecule toxin, albicidin, was also discovered in strains of *X. albilineans* [39,40,41,42]. The discovery of albicidin improved the understanding of pathogen virulence factors that contribute to colonization of host plants in the *X. albilineans-*sugarcane pathosystem [39,40]. Genome mining studies further identified new biosynthetic gene clusters of potentially new bioactive compounds in *Xanthomonas* species [37]. However, little effort has been made to understand the metabolome of xanthomonads. Discovery and understanding of the array of metabolites produced in species of this genus could result in the identification of new metabolites that improve our understanding of chemical components in host plant–pathogen interactions.

Our major aim in this study was to characterize the metabolome of *X. perforans,* a pathogen of bacterial spot of tomato (*Solanum lycopersicum* L.) as well as the metabolic changes in *X. perforans* following treatment with the small molecule carvacrol. Bacterial spot of tomato is a significant problem in almost all tomato-growing regions and is favored by warm temperatures, high rainfall, and humidity, where tomato fruit yield losses can reach up to 50% [43,44,45]. *X. perforans* is one of the four bacterial species causing bacterial spot in tomato worldwide; the other species include *X. euvesicatoria* (Xe), *X. vesicatoria* (Xv)*,* and *X. gardneri* (Xg) [45]. *X. perforans* was identified as an emergent T3 race, which subsequently displaced the previously dominant *X. euvesicatoria* strains in Florida [44]. It was reported that the *avrXv3* gene in *X. perforans* interacted with the *Xv3* gene in tomato in a gene-for-gene interaction manner [44]. Subsequently, *X. perforans* became the dominant strain in Florida [46,47,48]. *X. perforans* has also been identified on tomato in other geographic regions [49,50,51]. Control of *X. perforans* has been more difficult due to the emergence of copper-resistant strains in Florida and its ever-evolving genetic groups [52]. Recent efforts have focused on the use of small molecules for its control [53,54]. Carvacrol, a small molecule derived from plants mostly in the Lamiaceae family, has been shown to have antibacterial activities against the bacterial spot of tomato pathogen [53,54]. Furthermore, carvacrol was demonstrated to be effective against copper-resistant *X. perforans* strains in Florida and improve the efficacy of copper-based bactericides against the pathogen [54]. Carvacrol was also reported to prime tomato seeds against the pathogen, promoting root length and seedling vigor [54].

In this study, ultra-high-performance liquid chromatography coupled to orbitrap mass spectroscopy was used to characterize the totality of metabolites in a wild-type strain of *X. perforans*. We implemented time-series metabolomics analysis to understand the metabolic changes in *X. perforans* following treatment with carvacrol. Our results revealed the presence of novel putatively identified metabolites that were not previously reported in microbial systems or xanthomonads, suggesting multiple instances of horizontal gene transfer and convergent evolution of metabolites in the evolution of the pathogen. We also found significant temporal changes in metabolites that occurred following treatment with carvacrol. This study is important in the understanding of the metabolite diversity in *Xanthomonas* spp. and the metabolic changes that occurred in microbes due to the response to treatment with the small molecule, carvacrol.

## 2. Results

### 2.1. Metabolomic Profiling of X. perforans, and Annotation

In this study, 1477 and 1094 metabolites were identified in the positive and negative ion modes, respectively (Supplementary Figure S1). Out of this total of 2571, 620 and 439 peaks in the positive and negative mode, respectively, were identified as significant by ANOVA (*p* < 0.05) (Table 1). Metabolites with single hits from the search were designated as putatively identified metabolites. Supplementary Table S1 and S2 show annotated metabolites of the significant peaks in the positive and negative ion modes, respectively.

In general, metabolites belonging to different metabolic classes and groups were identified. Table 2 shows some of the metabolites that were identified in the *X perforans* metabolome in this study that are being reported for the first time in a microbe or *Xanthomonas* species. These include cyclobrassinone, sarmentosin, and monatin, which are known metabolites of plant systems and have not been previously reported in microbial systems. Additional plant metabolites include piperidine, enterodiol sulfate, and the β-carbolines harman, norharman, and an unannotated β-carboline (Table 2). Other unique microbial metabolites that are reported for the first time in *Xanthomonas perforans* include maculosin, pyrocoll, and saccharopine (Table 2, Supplementary Tables S1 and S2).

Some metabolites that are well-known drugs in the pharmaceutical industry were also annotated among the *X. perforans* metabolome in this study. Some of these metabolites include metharbital, etomidate, ozagrel, pirbuterol, and several amino acid derivates (Supplementary Tables S1 and S2). We also found metabolites in *X. perforans* that have been previously characterized to be important in inhibiting pathogen virulence and managing plant diseases. Table 3 shows examples of some identified metabolites in *X. perforans* that were previously shown to have promise in plant disease management. These metabolites broadly belong to alkaloids, amino acids/amines, polyamines, nucleotides/nucleosides, vitamins, and organic acids (Table 3). β-carboline and piperidine represent some of the alkaloids while L-cysteine, pipecolate, L-kynurenine, L-methionine, and proline represent some of the amino acids. At least three polyamines were putatively identified in *X. perforans* and these include spermidine, putrescine, and the acetylated N-acetyl putrescine (Table 3, Supplementary Table S1). Cytidine, hypoxanthine, uracil, monophosphates, and dimethirimol represent some of the nucleotides and nucleosides. Nicotinamide (vitamin B3), pyridoxine (B6), and biotin (B7) were some of the vitamins found in the *X. perforans* metabolome from this study.

### 2.2. Effect of Carvacrol on Metabolic Changes in a Time Course Experiment in X. perforans

**Figure 1:**
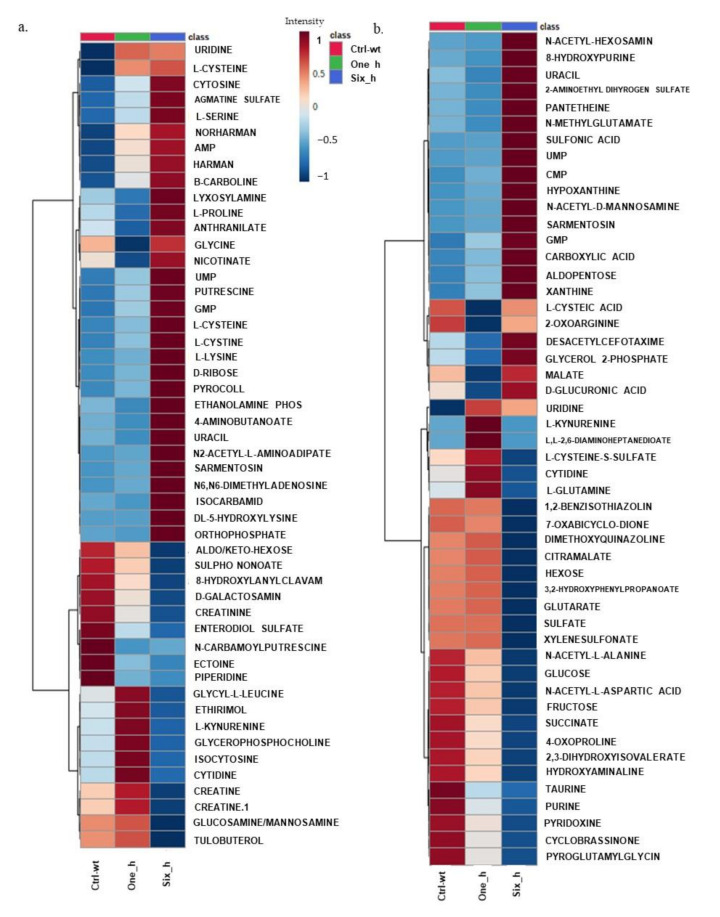
Heatmaps showing the effect of carvacrol on *X. perforans*. Panels (**a**,**b**) show hierarchical clustering of changes in metabolites in the time series in response to carvacrol treatment for the top 50 metabolites. Treatments include untreated wild type (ctrl-wt), samples at 1 h after treatment with carvacrol (One_h), and samples at 6 h after treatment with carvacrol (Six_h). Each treatment was replicated four times. A pooled sample from all four replicates for each treatment was included as a fifth replicate. The average intensities for the five replicates in each treatment are shown. The side bar represents the graduation in intensities, with 1 and −1 representing the highest and lowest intensity or concentration of metabolites, respectively, in both panels. See Supplementary Figure S3xi,xii for changes in all annotated metabolites in response to carvacrol treatment.

Univariate and multivariate analyses of the identified significant metabolites in *X. perforans* show that carvacrol has a global effect on the metabolome of *X. perforans*. The results for data normalization and correlation heatmaps for metabolites in both ion modes are shown in Supplementary Figure S3i–iv. Principal component analyses, partial least square discriminant analyses (PLS-DA), and sparse PLS-DA provided enough support for the variations seen in the data (Supplementary Figure S3v–x). Figure 1 shows the top 50 metabolites where changes in the average variation of metabolites in response to carvacrol occurred. Supplementary Figure S3xi–xii show the average variation in all putatively identified metabolites. Nine patterns of changes in metabolites were observed for the time series effect of carvacrol on *X. perforans* metabolites (Supplementary Table S3a). While many of the metabolites were significantly reduced in intensities at 1 and 6 h after treatment with carvacrol, others showed an increase at 1 and 6 h after treatment with carvacrol. A major pattern of changes in metabolites from the untreated control to 6h after treatment with carvacrol is metabolites that showed low/very low intensity constitutively (untreated wild type, control) and at 1 h but very high intensity at 6 h. This pattern is shown by 3-aminobutanoate, uracil, ethanolamine phosphate, orthophosphate, urocanate, anthranilate, DL-5-hydroxylysine, adenosine 5-monophosphate, norharman, harman, beta-carboline, L-cystine, D-ribose, sarmentosin, uridine 5-monophosphate, putrescine, guanosine 5-monophosphate, pyrocoll, cytosine, agmatine sulfate, L-cysteine, Aldopentose, xanthine, N-acetyl hexoseamine, Asparagine, hypoxanthine, CMP, N-acetyl-D_mannoseamine, 5-aminolevulinic acid, Pantetheine, proline, L-serine, carnosine, Alpha-aminoadipate, Malate, D-Glucoronic acid, citrulline, L-Histidine, and glycerol-2-phosphate. A second major pattern of variation is metabolites that have high intensity constitutively (untreated wild type) and reduced intensity at 1 and 6 h. This pattern is shown by creatine, pyridoxine, piperidine, N-carbamoylputrescine, dopamine, 5-oxo—L-proline, histamine, taurine, citrulline, pipecolate, glucose/fructose, 3-Sulfino-L-Alanine, N-Acetyl-L-Alanine, N-Acetyl-L-Aspartic acid, and 2,3-dihydroxyisovalerate. Other patterns of variation are as shown in Supplementary Table S3a.

### 2.3. Pathway Analysis of Significant Metabolites of X. perforans

**Figure 2:**
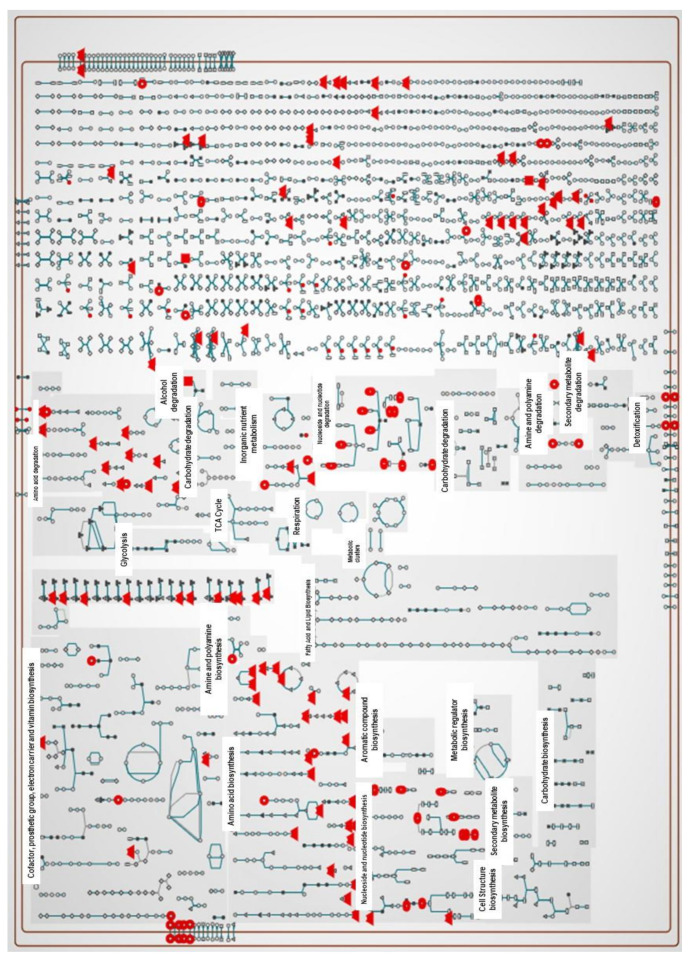
Metabolic pathways of *X. perforans* showing pathways of interaction of some of the annotated significant metabolites in the positive ion phase when the metabolites are superimposed on the cellular metabolic map of strain Xp91-118. a. Out of the input of the identified significant 81 metabolites in the positive phase, only 34 metabolites from our dataset were annotated on the cellular metabolic map of Xp91-118 on BioCyc. These metabolites include anthranilate, cytidine, ethanolamine, guanosine, UMP, putrescine, uracil, agmatine, glycero-3-phosphocholine, L-glutamine, L-proline, uridine, urocanate, adenosine, L-serine, 4-aminobutanoate phosphate, 5-aminolevulinate, L-cysteine, L-asparagine, nicotinate, L-lysine, L-isoleucine, glycine, L-cystathionine, taurine, L-methionine, pyridoxine, L-kynurenine, carbamoylputrescine, sulfate 2′-deoxycytidine, biotin, and orotate (pointing up triangles = Amino acids, Square = Carbohydrates, Vertical ellipse = Purines, Horizontal ellipse = Pyrimidines, Circle = Other metabolites. Analysis was carried out using the BioCyc pipeline).

**Figure 3:**
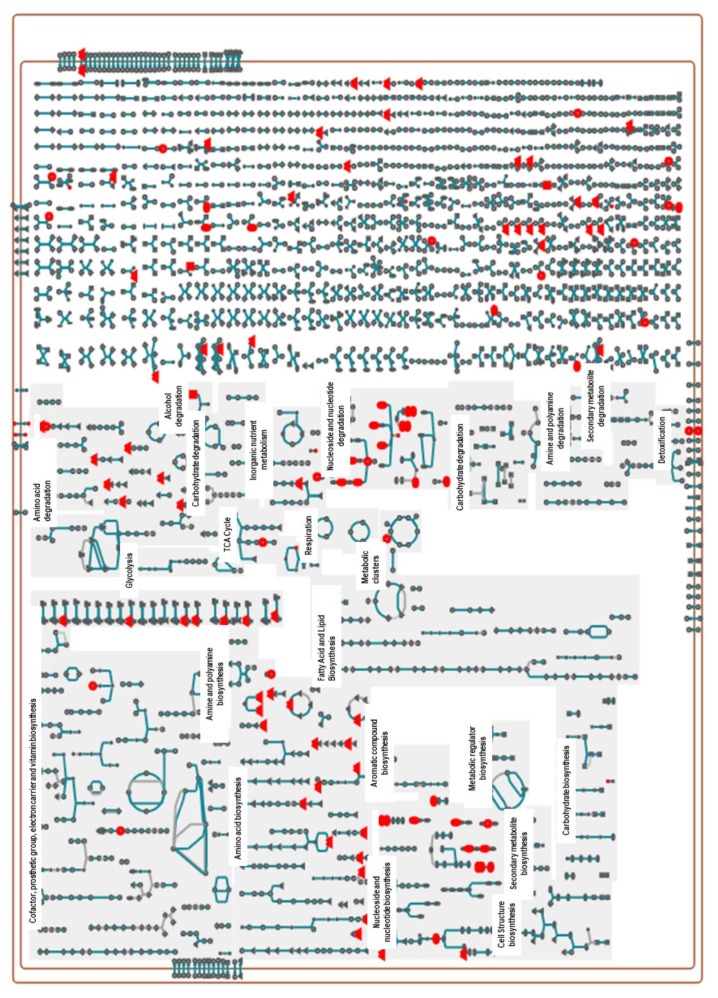
Metabolic pathways of *X. perforans* showing pathways of interaction of some of the annotated significant metabolites in the negative ion phase when the metabolites are superimposed on the cellular metabolic map of strain Xp91-118. Similarly, 34 metabolites out of the 86 significant metabolites from our dataset were annotated on the cellular metabolic map of Xp91-118 on BioCyc. These metabolites include malate, UMP, xanthine, uracil, hypoxanthine, CMP, cytidine, L-glutamine, guanosine, L-histidine, 5-aminolevulinate, L-serine, D-ribose 5-phosphate, urocanate, L-asparagine, adenosine, L-kynurenine, pyridoxine, L-proline, succinate, L-methionine, taurine, (indol-3-yl)acetate, L-cystathionine, glutarate, L-cysteate, sulfate, a carboxylate, L-ornithine, glycerol, L-saccharopine, uridine, and phosphate (pointing up triangles = Amino acids, Square = Carbohydrates, Vertical ellipse = Purines, Horizontal ellipse = Pyrimidines, Circle = Other metabolites. Analysis was carried out using the BioCyc pipeline).

About 217 metabolic pathways are annotated for the reference genome of the *X. perforans* Xp91-118 on the BioCyc website (www.biocyc.org, accessed on 18 May 2020–30 July 2021). The annotated metabolites in this study (Supplementary Tables S3 and S4) were used to carry out metabolic pathway analyses in BioCyc. Out of the 81 and 86 annotated metabolites in the positive and negative ion modes, respectively, the BioCyc metabolic pathway analyses pipeline only recognized 34 metabolites in each ion mode for *X perforans*. This resulted in the identification of 18 pathways for metabolites in the positive ion mode and 48 pathways for metabolites in the negative ion mode. Nine of the identified pathways were common in both the positive and negative ion mode, indicating strong evidence that carvacrol affected these pathways. These nine pathways included L-arginine biosynthesis I (via L-ornithine), L-cysteine biosynthesis VI (from L-methionine), L-histidine degradation II, L-lysine biosynthesis I, L-tryptophan degradation I (via anthranilate), pyrimidine nucleobase salvage I, superpathway of L-isoleucine biosynthesis I, superpathway of pyrimidine deoxyribonucleotides de novo biosynthesis, and the taurine degradation IV pathway. The cellular overview of the 34 annotated metabolites overlaid on Xp91-118 metabolic pathways is shown in Figure 2 and Figure 3, respectively, for metabolites in both the positive and negative ion modes.

**Figure 4:**
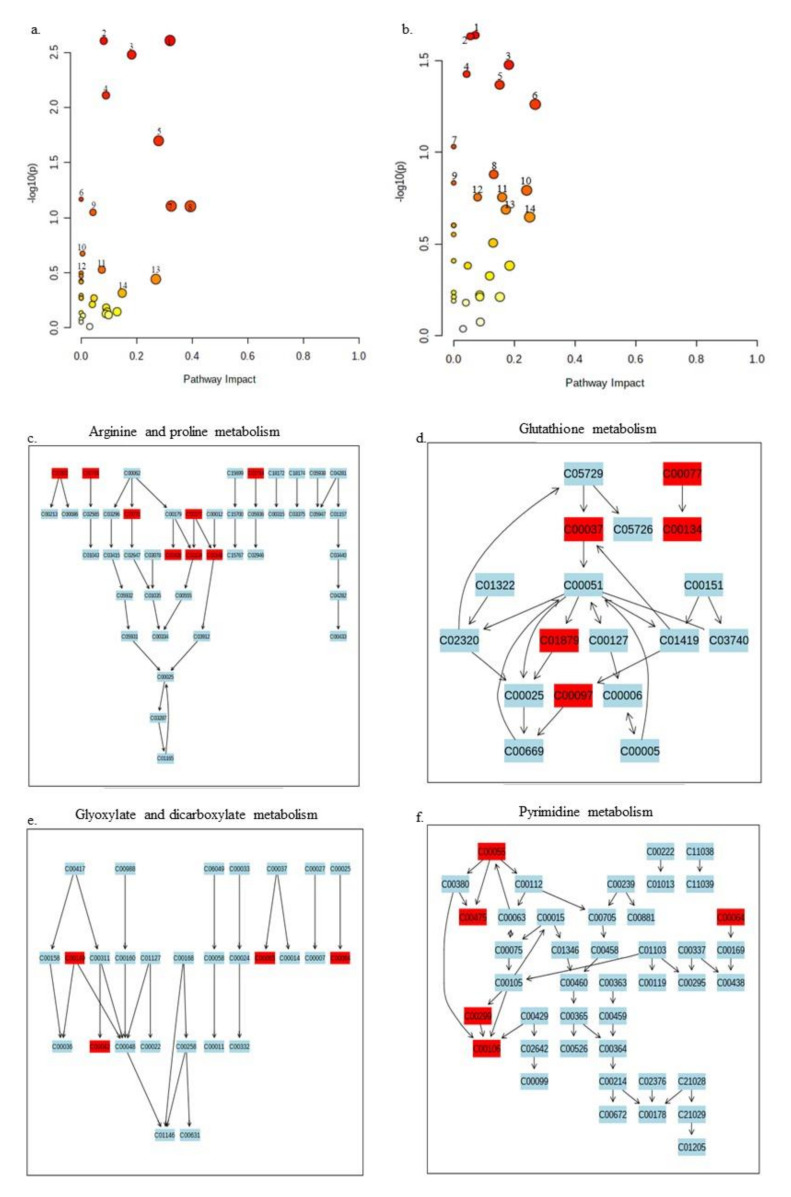
Simulated pathway analyses of significant metabolites in this study against pathways of *Pseudomonas putida* KT2440. The strength of the impact of the analyzed metabolites is indicated in red and yellow. A deep red color indicates that many metabolites with significant changes as a result of carvacrol application are found in the pathway, and this decreases to a different shade of red, yellow, and white (no metabolite in the pathway). The pathways that have more than one annotated metabolite in this study are labeled from 1–14 in both panels. Panel (**a**) shows pathways of positive ion mode metabolites. 1 = arginine and proline metabolism, 2 = glutathione metabolism, 3 = aminoacyl tRNA biosynthesis, 4 = pyrimidine metabolism, 5 = glycine, serine, threonine metabolism, 6 = cyanoamino metabolism, 7 = arginine metablosim, 8 = cysteine and methionine metabolism, 9 = sulfur metabolism, 10 = glyoxylate and dicarboxylate metabolism, 11 = lysine degradation, 12 = pantothenate and CoA biosynthesis, 13 = alanine, aspartate, and glutamate metabolism, 14 = methane metabolism). Panel (**b**) shows pathways of negative ion mode metabolites 1 = glyoxylate and dicarboxylate metabolism, 2 = pyrimidine metabolism 3 = aminoacyl tRNA biosynthesis 4 = sulfur metabolism, 5 = pantothenate and CoA biosynthesis, 6 = alanine, aspartate and glutamate metabolism, 7 = beta-alanine metabolism, 8 = purine metabolism, 9 = cyanoamino acid metabolism, 10 = arginine biosynthesis, 11 = lysine degradation, 12 = citrate (TCA) cycle, 13 = histidine metabolism, 14 = cysteine and methionine metabolism. Panels (**c**,**d**) shows the top two identified pathways for positive ion mode metabolites, Arginine and proline metabolism, and glutathione metabolism pathways. The metabolites in this study that are found in each pathway are colored in red (arginine and proline metabolism: C00300: Creatine, C00791: Creatinine, C02714: N-Acetylputrescine, C03771: 2-Oxoarginine, C00077: L-Ornithine, C00436: N-Carbamoylputrescine, C00134: Putrescine, C00149: Malate; glutathione metabolism: C00077: L-Ornithine; C00124: Putrescine; C00027: Glycine; C01879: 5-Oxoproline; C00097: L-Cysteine). Panels (**e**,**f**) similarly show the top two pathways for annotated metabolites in the negative ion mode in this study. The metabolites in this study that are found in each pathway are colored in red (glyoxylate and dicarboxylate metabolism: C00149: (S)-Malate, C00065: L-Serine, C00064: L-Glutamine, C00042: succinate; pyrimidine metabolism: C00055: CMP, C00475: Cytidine; C00064: L-Glutamine; C00299: Uridine; C00106: Uracil). Metabolites in the other identified pathways are shown in Supplementary Figure S2i.

Since studies on metabolomics for *Xanthomonas* species are limited and the pathways are poorly experimentally characterized, we analyzed annotated significant metabolites of *X. perforans* in our study against the well-studied *Pseudomonas putida* KT2440 annotated metabolic pathways available on the MetaboAnalyst platform (www.metaboanalyst.ca, accessed on 18 May 2020–30 July 2021). A total of 14 pathways in each phase were strongly affected (Figure 4a,b, Supplementary Figure S2i). The arginine and proline metabolism as well as glutathione metabolism pathways showed top effect as evidenced by the annotated metabolites found these pathways (Figure 4c,d). In the negative ion mode, glyoxylate and dicarboxylate metabolism as well as pyrimidine metabolism pathways represent the top pathways of interaction of the annotated metabolites (Figure 4e,f). The other pathways where the annotated metabolites showed marked effect in the simulated study are shown in Supplementary Figure S2i.

## 3. Discussion

### 3.1. LC-MS Identified Convergence of Chemicals from Diverse Systems in the Metabolome of X. perforans

Our study provides important insights into the diversity of metabolites in the plant pathogen *X. perforans.* Although previous studies suggested that species in the genus *Xanthomonas* are a potential reservoir of novel drugs, not much progress has been made in this field. In this study, metabolites that were previously only known in plant and microbial systems were putatively identified in *X. perforans*. Methoxybrassinin, cyclobrassinone, sarmentosin, and monatin were only reported in plant systems and this is the first report of these metabolites in a microbial system. Both methoxybrassinin and cyclobrassinone are metabolites originally identified in Brassicaceae [55,57,58]. Methoxybrassinin is a sulfur-containing phytoalexin that was first studied in the Brassicaceae family [55,108,109]. Subsequently, other plant species were shown to produce methoxybrassinins and other associated phytoalexins [110,111,112]. Similarly, cyclobrassinone was previously reported in *Brassica* [57,58]. While *X. perforans* has not been reported as a pathogen on any Brassicaceae species previously, another xanthomonad, *Xanthomonas campestris* pv. *campestris,* is an economically important pathogen in brassicas, causing black rot on many crucifers [56]. Perhaps, methoxybrassinin and cyclobrassinone are important in host–pathogen interaction between *Xanthomonas campestris* pv. *campestris* and their Brassicaceae hosts and may have acquired genes for these metabolites through horizontal gene transfer. Given the presence of this brassica metabolite in *X. perforans*, the methoxybrassinin pathway may have been acquired in a *Xanthomonas* ancestor and may be a common metabolite between the two *Xanthomonas* species. Sarmentosin may also be of similar evolution. Sarmentosin is an unsaturated γ-hydroxynitrile glucoside produced by plants and is often sequestered by lepidopterans [49]. Sequestration of sarmentosin has been the subject of several chemical ecological studies in the *Passiflora-*heliconiine butterfly system [59,60]. *X. perforans* has not been reported previously as a pathogen of a *Passiflora* species. However, another *Xanthomonas* species, *X. axonopodis* pv. *passiflorae*, causes bacterial leaf spot of *Passiflora* species [61,113]. The pathway for sarmentosin synthesis may also have been acquired through horizontal gene transfer and may be evolutionary conserved in an ancestral lineage of *Xanthomonas* species. Monatin is a naturally occurring sweetener found in *Sclerochiton ilicifolius* [62,63]. There are no reported *Xanthomonas* pathogens of *Sclerochiton ilicifolius*; however, the possibility of a *Xanthomonas* pathogen may not be ruled out as new wild hosts of *Xanthomonas* species are continually being reported [114,115].

The other plant metabolites identified in *X. perforans* include piperidine, the parent backbone for piperine in *Piper nigrum*, and enterodiol sulfate, an alkaloid found in flax seeds [64,65,67]. A *Xanthomonas* species, *X. campestris* pv. *betlicola*, has been previously reported on *P. nigrum* [66], suggesting that the metabolite may be evolutionary conserved in an ancestral lineage of *Xanthomonas* species. While no *Xanthomonas* pathogen has been reported on flax (*Linus* species), *X. campestris* pv. *phormiicola* causes bacterial streak on the unrelated New Zealand flax, *Phormium* species [116]. The β-carbolines harman, norharman and an unannotated β-carboline, which are commonly found in *Peganum harmala, Picrasma quassioides*, and many passion fruits, were also detected in *X. perforans *at different retention times. These metabolites are also found in fungi, insects, and marine animals [68,69,117]. β-carboline alkaloids have been widely studied in human systems as antioxidants and as markers of Parkinson’s disease, tremor, addiction, and cancers [118,119,120,121]. Harmine, harmaline, harman, and norharman, originally isolated from the seeds of *Pegannum harmala* L., are the most widely studied β-carboline alkaloids and have been shown to have antimicrobial activities that could potentially be important in plant protection [68,69,70]. While several plant pathogens have been isolated from *Peganum harmala* and *Picrasma quassioides,* no *Xanthomonas* species was reported [122]. However, the pathogenic lifestyle of *X. axonopodis* pv. *passiflorae* on *Passiflora* species has previously been described and represents a possible route for horizontal acquisition of the β-carboline genes found in *Passiflora* species [61].

Other novel metabolites identified in *X. perforans* include maculosin, pyrocoll, and saccharopine. Maculosin, a diketopiperazine metabolite, is a host-specific phytotoxin initially identified in *Alternaria alternata* [72]. Maculosin was also reported in *Lysobacter capsici, Streptomyces* species, and the non-obligate *Pseudomonas* strain 679-2 [123,124,125]. While it was initially described as a herbicide against knapweed (*Centaurea muculosa)*, maculosin has been shown to reduce the growth of plant pathogenic bacteria *X. axonopodis* and *Ralstonia solanacearum;* oomycetes *Phytophthora cactorum, P. capsici, P. cinnamomic, P. infestans*, and *P. ultimum;* and *Aspergillus niger, Candida. albicans,* and *Cryptococcus neoformans* [123,124,125]. Pyrocoll is a synthetic compound that was known to be a constituent of cigarette smoke but was subsequently detected in a *Streptomyces* species [71]. Pyrocoll exhibited biological activity against many microbes [71]. Saccharopine is a non-proteinogenic amino acid, which was originally detected in yeast, *Saccharomyces cerevisiae*, and has been demonstrated to function as a mitochondrial toxin [126].

Additionally, widely utilized drugs, such as crotamiton (for treating scabies and itching), enterodiol sulfate (modulates the immune system in humans and may have protective roles against the development of chronic diseases), valsartan (for lowering high blood pressure), metharbital (anti-convulsant), etomidate (anesthetic agent), ozagrel (antiplatelet agent), pirbuterol (bronchodilator), and many amino acid derivatives were putatively identified at different spectra in *X. perforans,* suggesting that the microbe is a drug bank of potentially medically relevant molecules [67,127,128,129,130].

Our findings, therefore, point not only to a neglected minefield for possible novel chemicals in the genus *Xanthomonas,* but convergence of metabolites that may have been gained through independent instances of horizontal gene transfer in the evolution of this pathogen. Convergent evolution of chemicals has been described in several biological systems and often point to coevolution of host–pathogen or predator–prey interactions. For example, the sesquiterpene olefin (*E*)-b-caryophyllene found in maize and *Arabidopsis* was also detected in the Asian lady beetle *Harmonia axyridis* [131,132]. Recently, it was shown that a fungus can co-opt ancient antimicrobial molecules to enhance their competitive abilities in the microbiome [133]. In this study, we provide evidence for metabolites only previously described in plant and microbial systems in *X. perforans* and provided instances of a *Xanthomonas–*plant interaction where such a pathogenic lifestyle has been reported. This suggests that the identified novel metabolite in *X perforans* may have been present in an ancestral lineage of *Xanthomonas* before the speciation event that led to the emergence of *X. perforans.* Characterization of these novel metabolites would provide insights into their functions and evolution.

In addition, this study provides a foundation for metabolite discovery in *X. perforans* as well as other species in the *Xanthomonas* genus. Traditionally, actinomycetes are known to be a vast reservoir of novel metabolites among bacterial microbes [134]. The discovery of albicidin and its novel biosynthetic gene clusters in *Xanthomonas* species spurred a call for active mining of bioactive compounds from species in the *Xanthomonas* genus [37]. However, little effort in drug mining in *Xanthomonas* species has ensued. We therefore demonstrate that important metabolites and their potential pathways are yet to be uncovered in species of this genus.

### 3.2. Identified Metabolites as Novel Management Strategies for Xanthomonas Plant Pathogens

Metabolites identified from plant–pathogen systems could be important for managing disease pathogens [18,83]. These metabolites exist at an equilibrium in microbial systems and alteration of the balance of important metabolites can provide an effective strategy in microbial disease management [135]. The metabolites identified in this study that have been experimentally shown to be important in plant disease management are described in Table 3. In *Arabidopsis thaliana,* L-kynurenine inhibited ethylene responses, a major plant hormone involved in plant development and stress tolerance, including resistance to pathogen infection [81]. Similarly, putrescine has been shown to enhance bacterial wilt disease caused by *Ralstonia solanacearum* in tomato [88]. In addition, spermidine, putrescine, and its derivates have been shown to be effective in fungal disease management [84,85,86,87]. Additionally, nucleotides and nucleosides have been shown to be effective in other plant disease systems and may be worth evaluating for their ability to manage bacterial spot of tomato. Interestingly, uridine-5-monophosphate was shown to control black rot in crucifers caused by a xanthomonad and could have similar effects on bacterial spot disease of tomato [100] (Table 3). Vitamins generally provide immunity against microbial infections in animals [136], and some have been shown to be important in plant disease management. Vitamins B1 (thiamine), B3 (niacin), B6 (pyridoxine), and K3 (menadione) were shown to have various degrees of efficacy against *Ralstonia solanacearum* and *Botrytis cinerea* in tomato (124). The plant and microbial metabolites that are newly identified in *X perforans* (Table 2) may equally be efficacious in managing the pathogen at doses that alter the normal metabolic requirement of the bacterium. Maculosin identified in this study has been shown to have potential in managing several plant diseases including those caused by *Xanthomonas* species [123,124,125]. Additionally, pyrocoll, sarmentosin, and piperine have all shown antimicrobial potential [71,136,137].

### 3.3. The Diversity of Metabolites in X. perforans Suggests the Possibility of Alternative Ecological Niches for Bacterial Spot Pathogens

Secondary metabolites found in organisms typically mediate interactions between the organisms, their hosts, and other competitors [14]. The diversity of metabolites in biological systems points to possible adaptation to diverse ecologies [138]. *X. perforans* was first reported in 1991 from Florida as an emergent genetic variant of the previously dominant species *X. euvesicatoria* in tomato fields [44,139]. It was discovered that *X. perforans* possesses an *avrXv3* gene, which interacts with the tomato *Xv3* gene in a gene-for-gene interaction [44]. Recent studies have explored the diversity within this species and unique pathotypes present in different geographies [52]. Strains of *X. perforans* have been recovered from forest tree seedlings, such as *Eucalyptus pellita*, although recent evaluations showed that they were not pathogenic on tomato or pepper [140]; Jones, J.B., personal communication]. Lesions were found on the leaves of the weed *Euphorbia heterophylla* after artificial inoculation with *X. perforans* [114]. Two other bacterial spot pathogens, *X. euvesicatoria* and *X. gardneri,* were found to be pathogenic on *Nicandra physaloides, Solanum americanum, Amaranthus lividus *(Amaranthaceae)*, Sida glomerata* (Malvaceae)*,* and *Emilia fosbergii* (Asteraceae) [114,115]. The identification of metabolites, such as maculosin in *X. perforans,* a metabolite that has been demonstrated to be important in the *Alternaria alternata–*knapweed interaction, may point to a similar lifestyle of the bacterial spot pathogen on weeds. Similarly, while phytoalexins are generally defense molecules produced by plants, the establishment of pathogenesis by some microbes relies on their abilities to detoxify the phytoalexins [141,142,143]. The presence of methoxybrassinin and cyclobrassinone in *X. perforans* metabolites may also suggest its possible interactions with hosts that produce the molecules. Additional studies characterizing the evolution and effect of these metabolites and the associated genes in the colonization of tomato and weeds would be required to draw conclusions.

### 3.4. Pathway Analyses Suggest That Carvacrol Elicits Global Metabolic Changes in X. perforans

Carvacrol has been studied as an antimicrobial, antioxidant, anticancer, and anti-inflammatory agent [144,145,146,147]. Carvacrol has also been suggested to antagonize quorum sensing in bacteria by potentially binding to LuxI or LuxR homologues, but evidence of direct binding to Lux proteins has not been confirmed [148,149]. Recently, carvacrol was shown to have bactericidal effects against *X. perforans* with potential for the management of tomato bacterial spot in greenhouse and field conditions [53,54]. However, studies on the mode of action of its activity against the pathogen was not shown. Understanding the mode of action of drugs is fundamentally important yet often difficult to achieve in drug discovery. Knowing the targets of drugs enables the characterization of off targets, thereby uncovering the overall importance of such drugs.

In this study, carvacrol elicited global changes in metabolites in *X. perforans* including amines, nucleotides/nucleosides, alkaloids, energy sources, and many unknown metabolites. Based on pathway analyses on BioCyc, at least 18 pathways were strongly affected by carvacrol in its activity against *X. perforans* in the positive phase while 48 pathways were affected in the negative phase. Nine of these identified pathways were common in both the positive and negative phases, suggesting a near certainty in the effect of carvacrol on these pathways. The nine pathways included pyrimidine metabolism, aminoacyl tRNA biosynthesis, sulfur metabolism, pantothenate and CoA biosynthesis, alanine, aspartate and glutamate metabolism, cysteine and methionine metabolism, and cyanoamino acid metabolism. Studies on Xp91-118 pathways provided limited information because many pathways are yet to be characterized for *X. perforans.* When the metabolites were analyzed against *Pseudomonas putida* KT2440 pathways, 13 pathways were common for metabolites in both the positive and negative phase spectra. Interestingly, some of these pathways acted upon by carvacrol were the same for both *X. perforans* 91-118 and *Pseudomonas putida* KT2440 in the analyses (Figure 2, Figure 3 and Figure 4).

**Figure 5:**
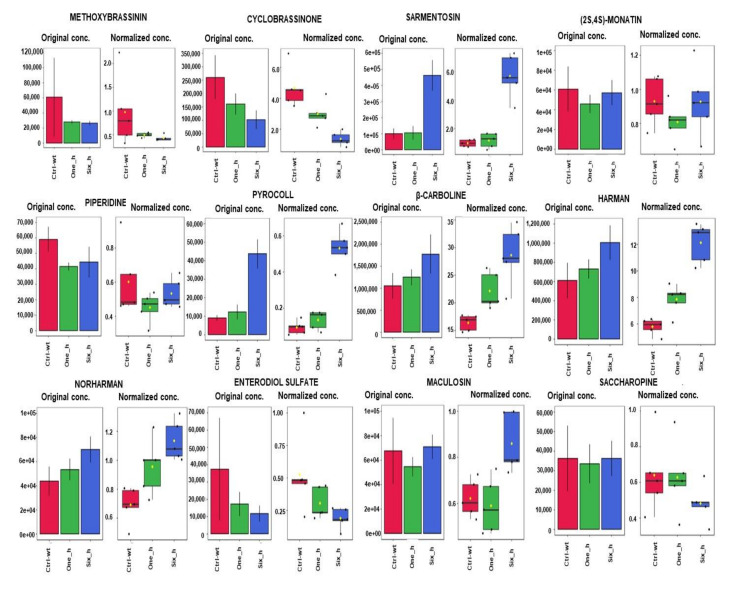
Graphical summary of the effect of carvacrol on changes of the newly identified metabolites in *X. perforans* in the time-series. Treatments include untreated wild type (ctrl-wt), samples at 1 h after treatment with carvacrol (One_h), and samples at 6 h after treatment with carvacrol (Six_h). Each treatment was replicated four times. A pooled sample from all four replicates for each treatment was included as a fifth replicate. Each bar in the plot represents the average intensities of the combined values of the five replicates in each treatment. The bar plots on the left show the original values. The box and whisker plots on the right summarize the normalized values.

Among these pathways, the effects of carvacrol on the pyrimidine pathway (in both phases) and purine pathway (in negative phase) suggested that carvacrol acted on the pathways associated with DNA synthesis in its activity against *X. perforans*. Naturally occurring DNA-damaging drugs, such as oxyresveratrol and resveratrol, have previously been demonstrated to have different effects on microbes [150,151]. Interestingly, resveratrol was shown to affect the purine, amino acid, and energy metabolism on *X. oryzae* pv. *oryzae,* the pathogen of rice bacterial blight [150]. Carvacrol has also been shown to act as a DNA-protecting agent in hepatic mitochondrial enzyme, a hepatic cancer cell line, and leukemic cells [151,152,153,154]. It is, therefore, interesting that carvacrol has effects on the purine and pyrimidine pathways. Interestingly, in the positive phase, cytidine production was decreased in *X. perforans* 6 h after carvacrol treatment, whereas the production of nucleotide/nucleoside uridine, uridine-5′-monophosphate, and guanosine-5-monophosphate was increased (Supplementary Figure S3xi). Similarly, in the negative phase, cytidine production was also decreased at 6 h post treatment with carvacrol, while the production of uracil, guanosine-5-monophosphate, and CMP was increased (Supplementary Figure S3xii). The increase in the intensities of these metabolites may indicate the attempts of the cells to protect themselves from DNA damage. It is also interesting that energy-related metabolites, such as glucose/fructose, hexose sugars, aldo/keto hexose, and 6-sugar alcohol, had decreased 6 h after treatment with carvacrol (Supplementary Figure S3xi,xii). The scavenging of the bacterium for an energy source is also corroborated by carvacrol activity on other pathways, such as the taurine (sulfur metabolism) pathway, which was detected in pathway analyses in both phases (Figure 2 and Figure 3; Supplementary Tables S3 and S4). Taurine is utilized by bacteria as a sulfur source under starvation conditions [155,156]. Interestingly, taurine was not detected in negative phase metabolites after 1 h following treatment with carvacrol (Supplementary Figure S3xii). *X. perforans* also showed differences in the newly identified metabolites at different times when treated with carvacrol (Figure 5). Sarmentosin, the β-carbolines, and maculosin especially showed elevated intensities at 6 h after treatment with carvacrol. In contrast, methoxybrassinin, cyclobrassinone, enterodiol sulfate, and saccharopine had reduced intensities at 6 h after treatment. However, since these are newly identified metabolites in *X. perforans* and their pathways are unknown, it is not possible identify the impact of carvacrol treatment on their pathways in this study.

The relative number of energy biosynthesis and degradation pathways affected by carvacrol in this study suggests that, perhaps, carvacrol acted largely on the energy biosynthesis pathways, thereby eliciting the global changes in the metabolome of *X. perforans.* A recent study has shown that global metabolic changes may be a consequence of a cascade of responses initiated by a unique metabolite [157,158]. It is, therefore, likely that the global changes identified in *X. perforans* were due to the activities of carvacrol on specific pathways. Gene expression studies would benefit from this information to improve the understanding of its mechanisms of action.

## 4. Materials and Methods

### 4.1. Sample Preparation

A wild-type strain of *X. perforans* Xp91-118 was used in this study. Strains of *X. perforans* were first grown on nutrient agar (NA) plates at 28 °C for 24 h, and the bacterial cells were inoculated in nutrient broth and grown overnight to achieve log phase growth. Overnight cultures of *X. perforans* in nutrient broth at 10^8^ cfu/mL were treated with carvacrol at various concentrations to determine the minimum inhibitory concentration (MIC) as described previously [54,159]. Untreated samples were included as the control. All treatments were replicated four times. Samples of *X. perforans* were taken 1 and 6 h after treatment with carvacrol. The bacterial cells were pelleted, flash frozen at −80 °C, and subsequently placed in dry ice for further processing. The experiment consisted of 12 samples, representing three treatments: ctrl-wt (untreated control), One_h (1h after treatment with carvacrol), Six_h (6h after treatment with carvacrol) and 4 replicates for each treatment. For each treatment, all four replicates were pooled together to form a fifth sample. All samples were analyzed at the Southeastern Center for Integrated Metabolomics (SECIM), University of Florida, Gainesville, Florida.

### 4.2. Protein Precipitation and Metabolomics Profiling

Extraction of samples for global metabolomics profiling was carried out using the SECIM in-house standard procedure. Briefly, pelleted bacterial cells were washed in 40 mM ammonium formate three times. The pellets were re-suspended in 50 µL of 5 mM ammonium acetate and homogenized three times with Zirconia beads on a bead beater for 30 s each time with 15 min of incubation on ice between each bead beater treatment. The protein concentration was measured, and cell homogenates were normalized to the protein concentration of 300 ug/mL. A mixture of labeled internal standards (20 µL) was added to each sample except for the extraction blank. Then, 1 mL of ice-cold 80% methanol was added to all samples and the extraction blank. Proteins were pelleted by centrifuging at 20,000× *g* for 10 min at 4 °C, while supernatants were collected and dried using a gentle stream of nitrogen. Samples were reconstituted with 30 uL of the injection standard solution and the extraction blank was reconstituted with 100 µL of 0.1% formic acid in water. All extractions were transferred to LC-MS vials for further analysis.

Global metabolomics profiling was performed on a Thermo Q-Exactive orbitrap mass spectrometer with Dionex UHPLC and an autosampler (Thermo Fisher, San Jose, CA, USA). All samples were analyzed in positive and negative heated electrospray ionization with a mass resolution of 35,000 at m/z 200 as separate injections. All samples were injected as provided. Separation was achieved on an ACE 18-pfp 100 × 2.1 mm, 2 µm column (Mac-Mod Analytical, Chadds Ford, PA, USA) with mobile phase A of 0.1% formic acid in water and mobile phase B of acetonitrile. The flow rate was 350 µL/min with a column temperature of 25 °C, and 4 µL were injected for negative ions and 2 µL for positive ions.

### 4.3. Preliminary Data Analysis and Annotation

Data from positive and negative ion modes were separately subjected to statistical analysis. All data were normalized to the sum of metabolites for each sample prior to analysis. MZmine *v.* 2 [160] (http://mzmine.github.io/, accessed on 18 May 2020–30 July 2021) was used to identify features, deisotopes, and to align features and perform gap filling to fill in any features that may have been missed in the first alignment algorithm. All adducts and complexes were identified and removed from the data set. The data were searched against the SECIM internal retention time metabolite library of 1100 compounds for identification. Subsequent searches against HMDB, METLIN, and KEGG were carried out manually.

Annotation in metabolomics generally includes assigning features with a putative metabolite, molecular formula, adducts, and neutral losses to facilitate accurate characterization and identification of annotated adduct peaks [8]. The chemical analysis working group of the Metabolomics Standards Initiative (MSI) defined four different levels of metabolite identification, which include identified metabolites (level 1), putatively annotated compounds (level 2), putatively characterized compound classes (level 3), and unknown compounds (level 4) [161,162]. In this study, we applied level 2 annotation. Molecular mass and retention time were initially used to search against the in-house metabolomics SECIM database at the University of Florida. Subsequent level 2 annotation focused primarily on the carvacrol-treated experiments. Mass and adduct type were used to search the Human Metabolome database (www.hmdb.ca, accessed on 18 May 2020–30 July 2021) and METLIN (https://metlin.scripps.edu/, accessed on 18 May 2020–30 July 2021).

### 4.4. Univariate and Multivariate Analysis of Metabolic Pathway

For univariate and multivariate analysis, MetaboAnalyst 5.0 https://www.metaboanalyst.ca/, accessed on 18 May 2020–30 July 2021) was used to normalize data, construct Volcano plots, and conduct principal component analysis (PCA), partial least square discriminant analysis (PLS-DA), dendograms, heatmaps, and other analyses [163]. Metabolic pathway analyses were carried out in MetaboAnalyst 5.0 and BioCyc (https://biocyc.org/, accessed on 18 May 2020–30 July 2021). Metabolic pathway analysis in BioCyc was carried out using both the metabolic coverage tool and overlaying identified significant metabolites on the cellular metabolic pathway overview of Xp91-118 to identify the pathways of involvement of the metabolites [164]. The BioCyc Metabolomic Pathway Coverage Report tool used a set of metabolites from a metabolomics experiment as input and computed a minimal cost of metabolites used as substrates in a set of metabolic pathways in a chosen organism [164]. Subsequently, the significant metabolites were used to search the well-annotated metabolic pathways of *Pseudomonas putida* KT2440 and *E. coli* K12 MG1655 at metaboanalyst.ca [163].

## 5. Conclusions

In this study, we utilized LC-MS-based untargeted metabolomics to provide insights into the metabolome of *X. perforans,* the pathogen of bacterial spot, an economically important disease in tomato. Annotation of significant metabolites improved the putative identification of metabolites in the *X. perforans* metabolome. This study provided insights into the chemical reservoir in *X. perforans* as well as the effects of carvacrol on the metabolome of the pathogen. Some of the identified metabolites included those that were previously not identified in a microbial system nor in a xanthomonad, suggesting yet to be characterized pathways in *X. perforans*.

Many significant metabolites were not identified and more than 70% of the significant metabolites (*p* < 0.05) remained unannotated in this study. Improving the annotation and identification of the annotated metabolites to level 1 standard (confidently identified and validated compounds confirming structure and annotation using reference standard) will confirm the metabolites in this *Xanthomonas* system and improve the understanding of the metabolites acted upon by carvacrol.

This study sheds light on the pathways of significant metabolites that are acted upon by carvacrol in its activity against *X. perforans.* While the number of unannotated metabolites is large and it is impossible to account for their roles, many pathways were shown to be affected by the activity of carvacrol. The pathways that utilize amino acids, DNA synthesis, and energy biosynthesis were all implicated in the activity of carvacrol. This study additionally provides a strong foundation to studying expressed genes associated with carvacrol activity in identified pathways to further improve the understanding of the activity of this chemical agent against *X. perforans.*

## Figures and Tables

**Table 1:** Number of significant metabolites in *X perforans* with *p*-value < 0.05 analyzed by ANOVA in this study.

Data Set	Significant Metabolites (*p*-Value < 0.05)		
	Identified Metabolites ^1^	Unidentified Metabolites	Total Metabolites
Positive Mode	81	559	620
Negative Mode	86	353	439
Total	167 ^2^	937	1059

^1^ Identified metabolites in this table are based on peak search against the in-house SECIM (Southeast Center for Integrated Metabolomics, University of Florida) database and subsequent search against HMDB (www.hmdb.ca, accessed on 18 May 2020–30 July 2021) and METLIN (www.metlin.scripps.edu, accessed on 18 May 2020–30 July 2021) databases. Only metabolites with single hits after the search are reported as putatively annotated at Metabolomics Standards Initiative (MSI) level 2. Please see Supplementary Table S1 and S2 for the list of all the annotated metabolites in this study. ^2^ This is the total number of metabolites identified by adding the number of metabolites annotated in the positive and negative ion modes, not the total of unique metabolites in each ion mode.

**Table 2:** Some metabolites of plants and other microbial systems of novel identification in the *Xanthomonas perforans* metabolome ^1^.

*X*. *perforans* Metabolites	Plant or Microbial Systems Where Metabolite Was Reported with References	Known *Xanthomonas* Pathogen of Plants with Metabolite with References	This Study as First Report in a Microbe (M) or *Xanthomonas* Species (X)
Methoxybrassinin A (M/Z = 281.0764; Rt = 10.70 min) ^2^	Brassicas [55]	*Xanthomonas campestris* pv. *campestris* on crucifers [56]	M
Cyclobrassinone (M/Z = 231.0245; Rt = 0.76 min)	Brassicas [57,58]	*Xanthomonas campestris* pv. *campestris* on crucifers [56]	M
Sarmentosin (M/Z = 276.1071; Rt = 3.26 min.)	Plants in the *Passiflora* genus and their heliconiine butterfly pests 9 [59,60]	*X. axonopodis* pv*. passiflorae* on *Passiflora* species [61]	M
(2s,4s)-monatin (M/Z = 293.1127; Rt = 7.66 min)	*Sclerochiton ilicifolius* [62,63]	None reported	M
Piperidine (M/Z = 86.0964; Rt = 1.07 min)	*Piper nigrum* [64,65]	*X. campestris pv. betlicola* on *P. nigrum* [66]	X
Enterodiol sulfate (M/Z = 383.1149; Rt = 0.73 min)	Flax seed [67]	None reported	X
β-Carboline (M/Z = 169.0759; Rt = 8.54 min)Harman (M/Z = 183.0913; Rt = 8.80 min)Norharman (M/Z = 157.1082; Rt = 1.35 min)	Many plants, including passion fruits, *Peganum harmala*, *Picrasma quassioides* [68,69,70]	*X. axonopodis* pv*. passiflorae* on *Passiflora* species [61]	X
Pyrocoll (M/Z = 187.0494; Rt = 6.30 min)	Smoke, Streptomyces [71]		X
Maculosin (M/Z = 261.1224; Rt = 7.69 min)	*Alternaria alternata* [72]		X
Saccharopine (M/Z = 275.1260; Rt = 0.76 min)	*Saccharomyces cerevisiae* [73]		X

^1^ For a complete list of all annotated metabolites, please see Supplementary Table S2 and S3. ^2^ M/Z = mass to charge ratio; Rt = Retention time in minutes (min).

**Table 3:** Annotated metabolites in *X. perforans* that have shown promise in plant disease management.

Class, Metabolites	Disease System	References
**Alkaloids**		
β-Carboline (M/Z = 169.0759; Rt = 8.54 min) ^1^	Rice Bacterial Blight, Kiwifruit Bacterial Canker, and Citrus Bacterial Canker	[74]
Piperidine (M/Z = 860964; Rt = 1.07 min)	*Xanthomonas oryzae pv. oryzae* and *X. axonopodis pv. citri*	[75,76]
**Amines**		
Amino Acids		
L-cysteine (M/Z = 122.0266; Rt = 0.76 min)	*Pseudomonas syringae* pv*. tomato* on *Arabidopsis*	[77]
Pipecolate/L-pipecolic ACID (M/Z = 130.0861; Rt = 1.25 min)	Systemic resistance in *Arabidopsis*, against bacterial plant pathogens	[78,79,80]
L-kynurenine (M/Z = 209.0915; Rt = 6.43 min)	Inhibit ethylene responses in *Arabidopsis* to disease resistance	[81]
L-methionine (M/Z = 150.0581; Rt = 1.43 min)	Powdery mildew on cucumber	[82]
Proline (M/Z = 114.0562; Rt = 0.85 min)	*Pectobacterium brasiliense*	[83]
**Polyamines**		
Spermidine (M/Z = 146.1649; Rt = 0.56 min)	*Blumeria graminis* f.sp. *hordei*, *Pyrenophora avenae*	[84,85,86,87]
Putrescine (M/Z = 89.1073; Rt = 0.60 min)	*Arabidopsis*; virulence factor in *Ralstonia solanacearum*	[88,89,90]
N-acetylputrescine (M/Z = 131.1176; Rt = 1.13 min)		[91]
N-Carbamoylputrescine (M/Z = 132.1130; Rt = 1.47 min)		
**Nucleotides/Nucleosides**		
Cytidine (M/Z = 244.0922; Rt = 1.66 min)	Cauliflower mosaic virus;	[92,93]
Hypoxanthine (M/Z = 137.0456; Rt = 2.11 min)	Powdery mildew in Arabidopsis	[94]
Uracil (M/Z = 113.0344; Rt = 1.44 min)	Programmed cell death	[95]
Adenosine-5′-Monophosphate (M/Z = 348.0694; Rt = 1.70 min)	Crown gall of *Vicia faba*	[96]
Guanosine-5-Monophosphate (M/Z = 364.0642; Rt = 2.01 min)	Arabidopsis, Tobacco	[97,98,99]
Uridine-5-Monophosphate (M/Z = 325.0421; Rt = 1.25 min)	Xanthomonas black rot of crucifers	[100]
Dimethirimol (M/Z = 210.1597; Rt = 7.12 min)	Fungicide	[101,102]
**Vitamins**		
Nicotinamide (M/Z = 123.0551; Rt = 1.70 (Vitamin B3)	Fusarium sp. On barley and Arabidopsis	[103]
Vitamin B6	*Botrytis cinerea* on tomato; *Rhizoctonia solani* on Potato and Arabidopsis	[104,105]
Pyridoxal (M/Z = 168.0649; Rt = 1.94 min (Vitamin B6)		
Pyridoxine (M/Z = 170.0804; Rt = 2.93 min (Vitamin B6)		
Pyridoxine (M/Z = 192.0627; Rt = 2.88 min (Vitamin B6)		
Biotin (M/Z = 245.0946; Rt = 6.58 min)	*Fusarium oxysporium* in *Arabidopsis*	[106]
**Organic Acids**		
Anthranilate (M/Z = 120.0443; Rt = 8.74 min)	Powdery mildew on barley;	[107]

^1^ M/Z = mass to charge ratio; Rt = Retention time in minutes (min).

## Data Availability

Data is available from corresponding author upon request. The data presented in this study is part of an ongoing study and all data will be made publicly available at the completion of the study.

## Supplementary Materials

The following are available online at https://www.mdpi.com/article/10.3390/metabo11120879/s1, Supplementary Figures S1–S3, Supplementary Tables S1–S5.

## References

[1] Vincent I.M., Ehmann D.E., Mills S.D., Perros M., Barrett M.P. (2016). Untargeted metabolomics to ascertain antibiotic modes of action. Antimicrob. Agents Chemother..

[2] Schiffman C., Petrick L., Perttula K., Yano Y., Carlsson H., Whitehead T., Metayer C., Hayes J., Rappaport S., Dudoit S. (2019). Filtering procedures for untargeted LC-MS metabolomics data. BMC Bioinform..

[3] Kellogg J., Kang S. (2020). Metabolomics, an Essential Tool in Exploring and Harnessing Microbial Chemical Ecology. Phytobiomes J..

[4] Sindelar M., Patti G.J. (2020). Chemical Discovery in the Era of Metabolomics. J. Am. Chem. Soc..

[5] Schrimpe-Rutledge A.C., Codreanu S.G., Sherrod S.D., McLean J.A. (2016). Untargeted Metabolomics Strategies—Challenges and Emerging Directions. J. Am. Soc. Mass Spectrom..

[6] Manier S.K., Keller A., Schäper J., Meyer M.R. (2019). Untargeted metabolomics by high resolution mass spectrometry coupled to normal and reversed phase liquid chromatography as a tool to study the in vitro biotransformation of new psychoactive substances. Sci. Rep..

[7] Salem M.A., De Souza L.P., Serag A., Fernie A.R., Farag M.A., Ezzat S.M., Alseekh S. (2020). Metabolomics in the Context of Plant Natural Products Research: From Sample Preparation to Metabolite Analysis. Metabolites.

[8] Domingo-Almenara X., Montenegro-Burke J.R., Benton H.P., Siuzdak G. (2018). Annotation: A Computational Solution for Streamlining Metabolomics Analysis. Anal. Chem..

[9] Pichersky E., Lewinsohn E. (2011). Convergent Evolution in Plant Specialized Metabolism. Annu. Rev. Plant Biol..

[10] Washburn J., Bird K., Conant G., Pires J.C. (2016). Convergent Evolution and the Origin of Complex Phenotypes in the Age of Systems Biology. Int. J. Plant Sci..

[11] Jensen N.B., Zagrobelny M., Hjernø K., Olsen C.E., Houghton-Larsen J., Borch J., Møller B.L., Bak S. (2011). Convergent evolution in biosynthesis of cyanogenic defence compounds in plants and insects. Nat. Commun..

[12] Stern D. (2013). The genetic causes of convergent evolution. Nat. Rev. Genet..

[13] Huang R., O’Donnell A.J., Barboline J.J., Barkman T.J. (2016). Convergent evolution of caffeine in plants by co-option of exapted ancestral enzymes. Proc. Natl. Acad. Sci. USA.

[14] Beran F., Köllner T.G., Gershenzon J., Tholl D. (2019). Chemical convergence between plants and insects: Biosynthetic origins and functions of common secondary metabolites. New Phytol..

[15] Suzuki T., Takahashi E. (1976). Caffeine biosynthesis in *Camellia sinensis*. Phytochemistry.

[16] Ashihara H., Monteiro A.M., Gillies F.M., Crozier A. (1996). Biosynthesis of Caffeine in Leaves of Coffee. Plant Physiol..

[17] Grenade N.L., Howe G.W., Ross A.C. (2021). The convergence of bacterial natural products from evolutionarily distinct pathways. Curr. Opin. Biotechnol..

[18] Stringlis I.A., Zhang H., Pieterse C.M.J., Bolton M.D., de Jonge R. (2018). Microbial small molecules—Weapons of plant subversion. Nat. Prod. Rep..

[19] Pang Z., Chen J., Wang T., Gao C., Li Z., Guo L., Xu J., Cheng Y. (2021). Linking Plant Secondary Metabolites and Plant Microbiomes: A Review. Front. Plant Sci..

[20] Verpoorte R., Choi Y.H., Kim H.K. (2010). Metabolomics: What’s new?. Flavour Fragr. J..

[21] Turi C., Finley J., Shipley P., Murch S.J., Brown P.N. (2015). Metabolomics for Phytochemical Discovery: Development of Statistical Approaches Using a Cranberry Model System. J. Nat. Prod..

[22] Caesar L.K., Kellogg J.J., Kvalheim O.M., Cech N.B. (2019). Opportunities and Limitations for Untargeted Mass Spectrometry Metabolomics to Identify Biologically Active Constituents in Complex Natural Product Mixtures. J. Nat. Prod..

[23] Schirawski J., Perlin M.H. (2018). Plant–Microbe Interaction 2017—The Good, the Bad and the Diverse. Int. J. Mol. Sci..

[24] Jacoby R.P., Koprivova A., Kopriva S. (2021). Pinpointing secondary metabolites that shape the composition and function of the plant microbiome. J. Exp. Bot..

[25] Mine A., Sato M., Tsuda K. (2014). Toward a systems understanding of plant–microbe interactions. Front. Plant Sci..

[26] Rose L.E., Overdijk E.J.R., Van Damme M. (2019). Small RNA molecules and their role in plant disease. Eur. J. Plant Pathol..

[27] Nazarov P.A., Baleev D.N., Ivanova M.I., Sokolova L.M., Karakozova M.V. (2020). Infectious plant diseases: Etiology, current status, problems and prospects in plant protection. Acta Nat..

[28] Speth E.B., Lee Y.N., He S.Y. (2007). Pathogen virulence factors as molecular probes of basic plant cellular functions. Curr. Opin. Plant Biol..

[29] Van Baarlen P., Van Belkum A., Summerbell R.C., Crous P.W., Thomma B.P. (2007). Molecular mechanisms of pathogenicity: How do pathogenic microorganisms develop cross-kingdom host jumps?. FEMS Microbiol. Rev..

[30] Neuhauser S., Kirchmair M., Bulman S., Bass D. (2014). Cross-kingdom host shifts of phytomyxid parasites. BMC Evol. Biol..

[31] Sharma M., Bhatt D. (2014). The circadian clock and defence signalling in plants. Mol. Plant Pathol..

[32] Mhlongo M.I., Piater L.A., Madala N.E., Labuschagne N., Dubery I.A. (2018). The Chemistry of Plant–Microbe Interactions in the Rhizosphere and the Potential for Metabolomics to Reveal Signaling Related to Defense Priming and Induced Systemic Resistance. Front. Plant Sci..

[33] Lin V.S. (2021). Interrogating Plant–Microbe Interactions with Chemical Tools: Click Chemistry Reagents for Metabolic Labeling and Activity-Based Probes. Molecules.

[34] Ryan R., Vorhölter F.-J., Potnis N., Jones J.B., Van Sluys M.-A., Bogdanove A.J., Dow J.M. (2011). Pathogenomics of *Xanthomonas*: Understanding bacterium–plant interactions. Nat. Rev. Genet..

[35] Timilsina S., Potnis N., Newberry E.A., Liyanapathiranage P., Iruegas-Bocardo F., White F.F., Goss E.M., Jones J.B. (2020). Xanthomonas diversity, virulence and plant–pathogen interactions. Nat. Rev. Genet..

[36] Büttner D., Bonas U. (2010). Regulation and secretion of *Xanthomonasvirulence* factors. FEMS Microbiol. Rev..

[37] Royer M., Koebnik R., Marguerettaz M., Barbe V., Robin G.P., Brin C., Carrere S., Gomez C., Hügelland M., Völler G.H. (2013). Genome mining reveals the genus *Xanthomonas* to be a promising reservoir for new bioactive non-ribosomally synthesized peptides. BMC Genom..

[38] Katzen F., Ferreiro D.U., Oddo C.G., Ielmini M.V., Becker A., Pühler A., Ielpi L. (1998). *Xanthomonas campestris* pv
*campestris* gum Mutants: Effects on Xanthan Biosynthesis and Plant Virulence. J. Bacteriol..

[39] Royer M., Costet L., Vivien E., Bes M., Cousin A., Damais A., Pieretti I., Savin A., Megessier S., Viard M. (2004). Albicidin Pathotoxin produced by *Xanthomonas albilineans* is encoded by three large PKS and NRPS genes present in a gene cluster also containing several putative modifying, regulatory, and resistance genes. Mol. Plant Microbe Interact..

[40] Hashimi S.M., Wall M.K., Smith A.B., Maxwell A., Birch R.G. (2007). The Phytotoxin Albicidin is a Novel Inhibitor of DNA Gyrase. Antimicrob. Agents Chemother..

[41] Kretz J., Kerwat D., Schubert V., Grätz S., Pesic A., Semsary S., Cociancich S., Royer M., Süssmuth R.D. (2015). Total Synthesis of Albicidin: A Lead Structure from *Xanthomonas albilineansfor* Potent Antibacterial Gyrase Inhibitors. Angew. Chem. Int. Ed..

[42] Hashimi S.M. (2019). Albicidin, a potent DNA gyrase inhibitor with clinical potential. J. Antibiot..

[43] Pohronezny K., Volin R.B. (1983). The effect of bacterial spot on yield and quality of fresh market tomatoes. Hort Sci..

[44] Stall R.E., Jones J.B., Minsavage G.V. (2009). Durability of Resistance in Tomato and Pepper to Xanthomonads Causing Bacterial Spot. Annu. Rev. Phytopathol..

[45] Potnis N., Timilsina S., Strayer A., Shantharaj D., Barak J.D., Paret M.L., Vallad G.E., Jones J.B. (2015). Bacterial spot of tomato and pepper: Diverse Xanthomonasspecies with a wide variety of virulence factors posing a worldwide challenge. Mol. Plant Pathol..

[46] Tudor-Nelson S.M., Minsavage G.V., Stall R.E., Jones J.B. (2003). Bacteriocin-Like Substances from Tomato Race 3 Strains of *Xanthomonas campestris* pv *vesicatoria*. Phytopathology.

[47] Horvath D.M., Stall R.E., Jones J.B., Pauly M.H., Vallad G.E., Dahlbeck D., Staskawicz B.J., Scott J.W. (2012). Transgenic Resistance Confers Effective Field Level Control of Bacterial Spot Disease in Tomato. PLoS ONE.

[48] Klein-Gordon J.M., Xing M.Y., Garrett K.A., Abrahamian P., Paret M.L., Minsavage G.V., Strayer-Scherer A.L., Fulton J.C., Timilsina S., Jones J.B. (2021). Assessing Changes and Associations in the *Xanthomonas perforans* Population Across Florida Commercial Tomato Fields Via a Statewide Survey. Phytopathology.

[49] Torelli E., Aiello D., Polizzi G., Firrao G., Cirvilleri G. (2015). Draft genome of a *Xanthomonas perforans* strain associated with pith necrosis. FEMS Microbiol. Lett..

[50] Osdaghi E., Taghavi S.M., Hamzehzarghani H., Fazliarab A., Lamichhane J.R. (2017). Monitoring the occurrence of tomato bacterial spot and range of the causal agent *Xanthomonas perforans* in Iran. Plant Pathol..

[51] Jibrin M.O., Potnis N., Timilsina S., Minsavage G.V., Vallad G.E., Roberts P.D., Jones J.B., Goss E.M. (2018). Genomic Inference of Recombination-Mediated Evolution in *Xanthomonas euvesicatoria* and *X. perforans*. Appl. Environ. Microbiol..

[52] Abrahamian P., Klein-Gordon J.M., Jones J.B., Vallad G.E. (2021). Epidemiology, diversity, and management of bacterial spot of tomato caused by Xanthomonas perforans. Appl. Microbiol. Biotechnol..

[53] Liu Q., Qiao K., Zhang S. (2019). Potential of a Small Molecule Carvacrol in Management of Vegetable Diseases. Molecules.

[54] Qiao K., Liu Q., Huang Y., Xia Y., Zhang S. (2020). Management of bacterial spot of tomato caused by copper-resistant Xanthomonas perforans using a small molecule compound carvacrol. Crop. Prot..

[55] Monde K., Sasaki K., Shirata A., Takasugi M. (1990). 4-Methoxybrassinin, a sulphur-containing phytoalexin from *Brassica oleracea*. Phytochemistry.

[56] Vicente J.G., Holub E.B. (2013). *Xanthomonas campestris* pv *Campestris* (cause of black rot of crucifers) in the genomic era is still a worldwide threat to brassica crops. Mol. Plant Pathol..

[57] Yannai S. (2004). Dictionary of Food Compounds with CD-ROM: Additives, Flavors, and Ingredients.

[58] Kirsch G., El-Sawy E., Abdelwahab A.B. (2018). Utilization of 1H-Indole-3-carboxaldehyde as a Precursor for the Synthesis of Bioactive Indole Alkaloids. Synthesis.

[59] Bjarnholt N., Rook F., Motawia M.S., Cornett C., Jørgensen C., Olsen C.E., Jaroszewski J.W., Bak S., Møller B.L. (2008). Diversification of an ancient theme: Hydroxynitrile glucosides. Phytochemistry.

[60] De Castro É.C.P., Zagrobelny M., Zurano J.P., Cardoso M.Z., Feyereisen R., Bak S. (2019). Sequestration and biosynthesis of cyanogenic glucosides in passion vine butterflies and consequences for the diversification of their host plants. Ecol. Evol..

[61] Goncalves E.R., Rosato Y.B. (2000). Genotypic characterization of xanthomonad strains isolated from passion fruit plants (*Passiflora* spp.) and their relatedness to different Xanthomonas species. Int. J. Syst. Evol. Microbiol..

[62] Fry J.C., Yurttas N., Biermann K.L., Lindley M.G., Goulson M.J. (2012). The Sweetness Concentration-Response of R,R-Monatin, a Naturally Occurring High-Potency Sweetener. J. Food Sci..

[63] Maharaj V., Moodley N., Vahrmeijer H. (2018). Characterization of natural monatin isomers, a high intensity sweetener from the plant Sclerochiton ilicifolius from South Africa. South Afr. J. Bot..

[64] Li S., Lei Y., Jia Y., Li N., Wink M., Ma Y. (2011). Piperine, a piperidine alkaloid from *Piper nigrum* re-sensitizes P-gp, MRP1 and BCRP dependent multidrug resistant cancer cells. Phytomedicine.

[65] Schnabel A., Athmer B., Manke K., Schumacher F., Cotinguiba F., Vogt T. (2021). Identification and characterization of piperine synthase from black pepper, *Piper nigrum* L.. Commun. Biol..

[66] Leyns F., Marcel D.C., Jean-Guy S., De Ley J. (1984). The Host Range of the Genus *Xanthomonas*. Bot. Rev..

[67] Saarinen N.M., Smeds A.I., Peñalvo J.L., Nurmi T., Adlercreutz H., Makela S. (2010). Flaxseed Ingestion Alters Ratio of Enterolactone Enantiomers in Human Serum. J. Nutr. Metab..

[68] Wrońska A.K., Boguś M.I. (2019). Harman and norharman, metabolites of the entomopathogenic fungus *Conidiobolus coronatus* (Entomophthorales), affect the serotonin levels and phagocytic activity of hemocytes, insect immunocompetent cells, in *Galleria mellonella* (Lepidoptera). Cell BioSci..

[69] Li S., Yang B., Zhang Q., Zhang J., Wang J., Wu W. (2010). Synthesis and bioactivity of beta-carboline derivatives. Nat. Prod. Commun..

[70] Nenaah G. (2010). Antibacterial and antifungal activities of (beta)-carboline alkaloids of *Peganum harmala* (L) seeds and their combination effects. Fitoterapia.

[71] Dietera A., Hamm A., Fiedler H.-P., Goodfellow M., Müller W.E.G., Brun R., Beil W., Bringmann G. (2003). Pyrocoll, an Antibiotic, Antiparasitic and Antitumor Compound Produced by a Novel Alkaliphilic *Streptomyces* Strain. J. Antibiot..

[72] Stierle A.C., Cardellina J.H., Strobel G.A. (1988). Maculosin, a host-specific phytotoxin for spotted knapweed from *Alternaria alternata*. Proc. Natl. Acad. Sci. USA.

[73] Saunders P., Broquist H.P. (1966). Saccharopine, an Intermediate of the Aminoadipic Acid Pathway of Lysine Biosynthesis. J. Biol. Chem..

[74] Liu H.-W., Ji Q.-T., Ren G.-G., Wang F., Su F., Wang P.-Y., Zhou X., Wu Z.-B., Li Z., Yang S. (2020). Antibacterial Functions and Proposed Modes of Action of Novel 1,2,3,4-Tetrahydro-β-carboline Derivatives that Possess an Attractive 1,3-Diaminopropan-2-ol Pattern against Rice Bacterial Blight, Kiwifruit Bacterial Canker, and Citrus Bacterial Canker. J. Agric. Food Chem..

[75] Wang P., Xiang M., Luo M., Liu H., Zhou X., Wu Z., Liu L., Li Z., Yang S. (2020). Novel piperazine-tailored ursolic acid hybrids as significant antibacterial agents targeting phytopathogens *Xanthomonas oryzae* pv *oryzae* and *X. axonopodis* pv. *citri* probably directed by activation of apoptosis. Pest Manag. Sci..

[76] Xiang M., Song Y., Ji J., Zhou X., Liu L., Wang P., Wu Z., Li Z., Yang S. (2020). Synthesis of novel 18 β-glycyrrhetinic piperazine amides displaying significant in vitro and in vivo antibacterial activities against intractable plant bacterial diseases. Pest Manag. Sci..

[77] Álvarez C., Bermúdez M.Á., Romero L.C., Gotor C., García I. (2012). Cysteine homeostasis plays an essential role in plant immunity. New Phytol..

[78] Chen Y.-C., Holmes E.C., Rajniak J., Kim J.-G., Tang S., Fischer C.R., Mudgett M.B., Sattely E.S. (2018). N-hydroxy-pipecolic acid is a mobile metabolite that induces systemic disease resistance in *Arabidopsis*. Proc. Natl. Acad. Sci. USA.

[79] Hartmann M., Zeier T., Bernsdorff F., Reichel-Deland V., Kim D., Hohmann M., Scholten N., Schuck S., Bräutigam A., Hölzel T. (2018). Flavin monooxygenase-generated N-hydroxypipecolic acid is a critical element of plant systemic immunity. Cell.

[80] Wang C., Liu R., Lim G.-H., de Lorenzo L., Yu K., Zhang K., Hunt A.G., Kachroo A., Kachroo P. (2018). Pipecolic acid confers systemic immunity by regulating free radicals. Sci. Adv..

[81] He W., Brumos J., Li H., Ji Y., Ke M., Gong X., Zeng Q., Li W., Zhang X., An F. (2011). A Small-Molecule Screen Identifies l-Kynurenine as a Competitive Inhibitor of TAA1/TAR Activity in Ethylene-Directed Auxin Biosynthesis and Root Growth in Arabidopsis. Plant Cell.

[82] Dekker J. (1969). L-methionine induced inhibition of powdery mildew and its reversal by folic acid. Eur. J. Plant Pathol..

[83] Joshi J.R., Yao L., Charkowski A.O., Heuberger A.L. (2021). Metabolites from Wild Potato Inhibit Virulence Factors of the Soft Rot and Blackleg Pathogen *Pectobacterium brasiliense*. Mol. Plant Microbe Interact..

[84] Cowley T., Walters D.R. (2002). Polyamine metabolism in barley reacting hypersensitively to the powdery mildew fungus *Blumeria graminisf*. sp *hordei*. Plant Cell Environ..

[85] El Ghachtouli N., Martin-Tanguy J., Paynot M., Gianinazzi S. (1996). First-report of the inhibition of arbuscular mycorrhizal infection of *Pisum sativumby* specific and irreversible inhibition of polyamine biosynthesis or by gibberellic acid treatment. FEBS Lett..

[86] Walters D., Meurer-Grimes B., Rovira I. (2001). Antifungal activity of three spermidine conjugates. FEMS Microbiol. Lett..

[87] Walters D.R. (2003). Polyamines and plant disease. Phytochemistry.

[88] Lowe-Power T.M., Hendrich C.G., von Roepenack-Lahaye E., Li B., Wu D., Mitra R., Dalsing B.L., Ricca P., Naidoo J., Cook D. (2018). Metabolomics of tomato xylem sap during bacterial wilt reveals Ralstonia solanacearum produces abundant putrescine, a metabolite that accelerates wilt disease. Environ. Microbiol..

[89] Liu C., Atanasov K.E., Arafaty N., Murillo E., Tiburcio A.F., Zeier J., Alcázar R. (2020). Putrescine elicits ROS -dependent activation of the salicylic acid pathway in *Arabidopsis thaliana*. Plant Cell Environ..

[90] Liu C., Alcázar R. (2021). A new insight into the contribution of putrescine to defense in *Arabidopsis thaliana*. Plant Signal. Behav..

[91] Walters D.R., Mackintosh C.A. (1997). Control of plant disease by perturbation of fungal polyamine metabolism. Physiol. Plant..

[92] Chen M., Herde M., Witte C.-P. (2016). Of the nine cytidine deaminase like genes in *Arabidopsis thaliana* eight are pseudogenes and only one is required to maintain pyrimidine homeostasis in vivo. Plant Physiol..

[93] Martín S., Cuevas J.M., Grande-Pérez A., Elena S.F. (2017). A putative antiviral role of plant cytidine deaminases. F1000Research.

[94] Ma X., Wang W.-M., Bittner F., Schmidt N., Berkey R., Zhang L., King H., Zhang Y., Feng J., Wen Y. (2016). Dual and Opposing Roles of Xanthine Dehydrogenase in Defense-Associated Reactive Oxygen Species Metabolism in Arabidopsis. Plant Cell.

[95] Stasolla C., Loukanina N., Yeung E.C., Thorpe T.A. (2004). Alterations in pyrimidine nucleotide metabolism as an early signal during the execution of programmed cell death in tobacco BY-2 cells. J. Exp. Bot..

[96] Niles R.M., Mount M.S. (1974). Failure to Detect Cyclic 3′, 5′-Adenosine Monophosphate in Healthy and Crown Gall Tumorous Tissues of *Vicia faba*. Plant Physiol..

[97] Durner J., Wendehenne D., Klessig D.F. (1998). Defense gene induction in tobacco by nitric oxide, cyclic GMP, and cyclic ADP-ribose. Proc. Natl. Acad. Sci. USA.

[98] Hao F., Zhao S., Dong H., Zhang H., Sun L., Miao C. (2010). Nia1 and Nia2 are Involved in Exogenous Salicylic Acid-induced Nitric Oxide Generation and Stomatal Closure in Arabidopsis. J. Integr. Plant Biol..

[99] Gross I., Durner J. (2016). In Search of Enzymes with a Role in 3′, 5′-Cyclic Guanosine Monophosphate Metabolism in Plants. Front. Plant Sci..

[100] Feng F., Yang F., Rong W., Wu X., Zhang J., Chen S., He C., Zhou J.-M. (2012). A Xanthomonas uridine 5′-monophosphate transferase inhibits plant immune kinases. Nat. Cell Biol..

[101] Bent K.J. (1970). Fungitoxic action of dimethirimol and ethirimol. Ann. Appl. Biol..

[102] Vielba-Fernández A., Polonio Á., Ruiz-Jiménez L., De Vicente A., Pérez-García A., Fernández-Ortuño D. (2020). Fungicide Resistance in Powdery Mildew Fungi. Microorganisms.

[103] Miwa A., Sawada Y., Tamaoki D., Hirai M.Y., Kimura M., Sato K., Nishiuchi T. (2017). Nicotinamide mononucleotide and related metabolites induce disease resistance against fungal phytopathogens in Arabidopsis and barley. Sci. Rep..

[104] Zhang Y., Liu B., Li X., Ouyang Z., Huang L., Hong Y., Zhang H., Li D., Song F. (2014). The de novo Biosynthesis of Vitamin B6 Is Required for Disease Resistance Against Botrytis cinerea in Tomato. Mol. Plant Microbe Interact..

[105] Samsatly J., Bayen S., Jabaji S.H. (2020). Vitamin B6 Is Under a Tight Balance During Disease Development by Rhizoctonia solani on Different Cultivars of Potato and on *Arabidopsis thaliana* Mutants. Front. Plant Sci..

[106] Tintor N., Paauw M., Rep M., Takken F.L.W. (2020). The root-invading pathogen Fusarium oxysporum targets pattern-triggered immunity using both cytoplasmic and apoplastic effectors. New Phytol..

[107] Hu P., Meng Y., Wise R.P. (2009). Functional Contribution of Chorismate Synthase, Anthranilate Synthase, and Chorismate Mutase to Penetration Resistance in Barley–Powdery Mildew Interactions. Mol. Plant Microbe Interact..

[108] Chripkova M., Drutovic D., Pilatova M., Mikes J., Budovska M., Vaskova J., Broggini M., Mirossay L., Mojzis J. (2014). Brassinin and its derivatives as potential anticancer agents. Toxicol. Vitr..

[109] Klein A.P., Sattely E.S. (2017). Biosynthesis of cabbage phytoalexins from indole glucosinolate. Proc. Natl. Acad. Sci. USA.

[110] Pedras M.S.C., Okinyo D.P.O. (2006). En route to erucalexin: A unique rearrangement in the crucifer phytoalexin biosynthetic pathway. Chem. Commun..

[111] Pedras M.S.C., Okinyo D.P.O. (2006). Syntheses of perdeuterated indoles and derivatives as probes for the biosyntheses of crucifer phytoalexins. J. Label. Compd. Radiopharm..

[112] Ahuja I., Kissen R., Bones A.M. (2012). Phytoalexins in defense against pathogens. Trends Plant Sci..

[113] Munhoz C.D.F., Santos A., Arenhart R., Santini L., Monteiro-Vitorello C.B., Vieira M.L.C. (2015). Analysis of plant gene expression during passion fruit- *Xanthomonas axonopodis* interaction implicates lipoxygenase 2 in host defence. Ann. Appl. Biol..

[114] Araújo E.R., Costa J.R., Pontes N.C., Quezado-Duval A.M. (2015). *Xanthomonas perforans* and *X. gardneri* associated with bacterial leaf spot on weeds in Brazilian tomato fields. Eur. J. Plant Pathol..

[115] Santos L.V.S., Melo E.A., Silva A.M.F., Félix K.C.S., Quezado-Duval A.M., Albuquerque G.M.R., Gama M.A.S., Souza E.B. (2020). Weeds as alternate hosts of *Xanthomonas euvesicatoria* pv *euvesicatoria* and *X. campestris* pv. *campestris* in vegetable-growing fields in the state of Pernambuco, Brazil. Trop. Plant Pathol..

[116] Tamura K., Takikawa Y., Tsuyumu S., Goto M., Watanabe M. (1992). Coronatine Production by *Xanthomonas campestris* pv *phormiicola*. Jpn. J. Phytopathol..

[117] Szabó T., Volk B., Milen M. (2021). Recent Advances in the Synthesis of β-Carboline Alkaloids. Molecules.

[118] Piechowska P., Zawirska-Wojtasiak R., Mildner-Szkudlarz S. (2019). Bioactive β-Carbolines in Food: A Review. Nutrients.

[119] Spies C.D., Rommelspacher H., Schnapper C., Muller C., Marks C., Berger G., Conrad C., Blum S., Specht M., Hannemann L. (1995). beta-Carbolines in Chronic Alcoholics Undergoing Elective Tumor Resection. Alcohol. Clin. Exp. Res..

[120] Rommelspacher H., Dufeu P., Schmidt L.G. (1996). Harman and Norharman in Alcoholism: Correlations with Psychopathology and Long-Term Changes. Alcohol. Clin. Exp. Res..

[121] Pfau W., Skog K. (2004). Exposure to β-carbolines norharman and harman. J. Chromatogr. B.

[122] Bibi F. (2017). Diversity of antagonistic bacteria isolated from medicinal plant *Peganum harmala* L.. Saudi J. Biol. Sci..

[123] Cain C.C., Lee D., Waldo R.H., Henry A.T., Casida E.J., Wani M.C., Wall M.E., Oberlies N.H., Falkinham J.O. (2003). Synergistic Antimicrobial Activity of Metabolites Produced by a Nonobligate Bacterial Predator. Antimicrob. Agents Chemother..

[124] Puopolo G., Cimmino A., Palmieri M., Giovannini O., Evidente A., Pertot I. (2014). Lysobacter capsici AZ78 produces cyclo(l -Pro-l -Tyr), a 2,5-diketopiperazine with toxic activity against sporangia of *Phytophthora infestans* and *Plasmopara viticola*. J. Appl. Microbiol..

[125] Wattana-Amorn P., Charoenwongsa W., Williams C., Crump M., Apichaisataienchote B. (2016). Antibacterial activity of cyclo(L-Pro-L-Tyr) and cyclo(D-Pro-L-Tyr) from *Streptomyces* sp. strain 22-4 against phytopathogenic bacteria. Nat. Prod. Res..

[126] Zhou J., Wang X., Wang M., Chang Y., Zhang F., Ban Z., Tang R., Gan Q., Wu S., Guo Y. (2019). The lysine catabolite saccharopine impairs development by disrupting mitochondrial homeostasis. J. Biol..

[127] Moore P.F., Constantine J.W., Barth W.E. (1978). Pirbuterol, a selective beta-2 adrenergic bronchodilator. J. Pharmacol. Exp. Ther..

[128] Richards D.M., Brogden R.N. (1985). Pirbuterol A Preliminary Review of its Pharmacological Properties and Therapeutic Efficacy in Reversible Bronchospastic Disease. Drugs.

[129] Kittaka H., Yamanoi Y., Tominaga M. (2017). Transient receptor potential vanilloid 4 (TRPV4) channel as a target of crotamiton and its bimodal effects. Pflüger’s Archiv Gesamte Physiol. Menschen Tiere.

[130] Corsini E., Dell’Agli M., Facchi A., De Fabiani E., Lucchi L., Boraso M.S., Marinovich M., Galli C.L. (2010). Enterodiol and Enterolactone Modulate the Immune Response by Acting on Nuclear Factor-κB (NF-κB) Signaling. J. Agric. Food Chem..

[131] Brown A.E., Riddick E.W., Aldrich J.R., Holmes W.E. (2006). Identification of (−)-β-Caryophyllene as a Gender-Specific Terpene Produced by the Multicolored Asian Lady Beetle. J. Chem. Ecol..

[132] Kollner T.G., Held M., Lenk C., Hiltpold I., Turlings T.C., Gershenzon J., Degenhardt J. (2008). A Maize (E)-β-Caryophyllene Synthase Implicated in Indirect Defense Responses against Herbivores Is Not Expressed in Most American Maize Varieties. Plant Cell.

[133] Snelders N.C., Petti G.C., Berg G.C.M.V.D., Seidl M.F., Thomma B.P.H.J. (2021). An ancient antimicrobial protein co-opted by a fungal plant pathogen for in planta mycobiome manipulation. Proc. Natl. Acad. Sci. USA.

[134] Jakubiec-Krzesniak K., Rajnisz-Mateusiak A., Guspiel A., Ziemska J., Solecka J. (2018). Secondary Metabolites of Actinomycetes and their Antibacterial, Antifungal and Antiviral Properties. Pol. J. Microbiol..

[135] Cabral D., Penumutchu S., Reinhart E.M., Zhang C., Korry B.J., Wurster J.I., Nilson R., Guang A., Sano W.H., Rowan-Nash A.D. (2019). Microbial Metabolism Modulates Antibiotic Susceptibility within the Murine Gut Microbiome. Cell Metab..

[136] Zou L., Hu Y.-Y., Chen W.-X. (2015). Antibacterial mechanism and activities of black pepper chloroform extract. J. Food Sci. Technol..

[137] Boriollo M.F.G., Marques M.B., Da Silva T.A., Da Silva J.J., Dias R.A., Filho T.H.N.S., Melo I.L.R., Dias C.T.D.S., Bernardo W.L.D.C., Oliveira N.D.M.S. (2020). Antimicrobial potential, phytochemical profile, cytotoxic and genotoxic screening *of Sedum praealtum* A. DC. (balsam). BMC Complement. Med. Ther..

[138] Wink M. (2003). Evolution of secondary metabolites from an ecological and molecular phylogenetic perspective. Phytochemistry.

[139] Jones J.B., Lacy G.H., Bouzar H., Stall R.E., Schaad N.W. (2004). Reclassification of the Xanthomonads Associated with Bacterial Spot Disease of Tomato and Pepper. Syst. Appl. Microbiol..

[140] Bophela K.N., Venter S.N., Wingfield M.J., Duran A., Tarigan M., Coutinho T.A. (2019). *Xanthomonas perforans*: A tomato and pepper pathogen associated with bacterial blight and dieback of *Eucalyptus pellita* seedlings in Indonesia. Australas. Plant Pathol..

[141] Sbaghi M., Jeandet P., Bessis R., Leroux P. (1996). Degradation of stilbene-type phytoalexins in relation to the pathogenicity of *Botrytis cinerea* to grapevines. Plant Pathol..

[142] Jeandet P., Hébrard C., Deville M.-A., Cordelier S., Dorey S., Aziz A., Crouzet J. (2014). Deciphering the Role of Phytoalexins in Plant-Microorganism Interactions and Human Health. Molecules.

[143] Raasch-Fernandes L.D., Bonaldo S.M., Rodrigues D., Junior G.M.V., Schwan-Estrada K.R.F., Da Silva C.R., Verçosa A.G.A., De Oliveira D.L., DeBiasi B.W. (2019). Induction of phytoalexins and proteins related to pathogenesis in plants treated with extracts of cutaneous secretions of southern Amazonian Bufonidae amphibians. PLoS ONE.

[144] Ultee A., Smid E. (2001). Influence of carvacrol on growth and toxin production by *Bacillus cereus*. Int. J. Food Microbiol..

[145] Xu J., Zhou F., Ji B.-P., Pei R.-S., Xu N. (2008). The antibacterial mechanism of carvacrol and thymol against *Escherichia coli*. Lett. Appl. Microbiol..

[146] Churklam W., Chaturongakul S., Ngamwongsatit B., Aunpad R. (2020). The mechanisms of action of carvacrol and its synergism with nisin against *Listeria monocytogenes* on sliced bologna sausage. Food Control..

[147] Alagawany M., Farag M.R., Abdelnour S.A., ElNesr S.S. (2021). A review on the beneficial effect of thymol on health and production of fish. Rev. Aquac..

[148] Joshi J.R., Khazanov N., Senderowitz H., Burdman S., Lipsky A., Yedidia I. (2016). Plant phenolic volatiles inhibit quorum sensing in pectobacteria and reduce their virulence by potential binding to ExpI and ExpR proteins. Sci. Rep..

[149] Joshi J.R., Khazanov N., Khadka N., Charkowski A.O., Burdman S., Carmi N., Yedidia I., Senderowitz H. (2020). Direct Binding of Salicylic Acid to *Pectobacterium* N-Acyl-Homoserine Lactone Synthase. ACS Chem. Biol..

[150] Luo H.-Z., Guan Y., Yang R., Qian G.-L., Yang X.-H., Wang J.-S., Jia A.-Q. (2020). Growth inhibition and metabolomic analysis of *Xanthomonas oryzae* pv *oryzae* treated with resveratrol. BMC Microbiol..

[151] Radapong S., Sarker S.D., Ritchie K.J. (2020). Oxyresveratrol Possesses DNA Damaging Activity. Molecules.

[152] Horvathova E., Navarova J., Galova E., Sevcovicova A., Chodakova L., Snahnicanova Z., Melusova M., Kozics K., Slamenova D. (2014). Assessment of Antioxidative, Chelating, and DNA-Protective Effects of Selected Essential Oil Components (Eugenol, Carvacrol, Thymol, Borneol, Eucalyptol) of Plants and Intact Rosmarinus officinalis Oil. J. Agric. Food Chem..

[153] Aristatile B., Al-Numair K.S., Al-Assaf A.H., Pugalendi K.V. (2011). Pharmacological effect of carvacrol on d-galactosamine-induced mitochondrial enzymes and DNA damage by single-cell gel electrophoresis. J. Nat. Med..

[154] Melusova M., Slamenova D., Kozics K., Jantova S., Horvathova E. (2014). Carvacrol and rosemary essential oil manifest cytotoxic, DNA-protective and pro-apoptotic effect having no effect on DNA repair. Neoplasma.

[155] van der Ploeg J.R., Weiss M.A., Saller E., Nashimoto H., Saito N., Kertesz M.A., Leisinger T. (1996). Identification of sulfate starvation-regulated genes in Escherichia coli: A gene cluster involved in the utilization of taurine as a sulfur source. J. Bacteriol..

[156] Eichhorn E., van der Ploeg J.R., Kertesz M., Leisinger T. (1997). Characterization of α-Ketoglutarate-dependent Taurine Dioxygenase from *Escherichia coli*. J. Biol. Chem..

[157] Borchert A.J., Gouveia G.J., Edison A.S., Downs D.M. (2020). Proton Nuclear Magnetic Resonance Metabolomics Corroborates Serine Hydroxymethyltransferase as the Primary Target of 2-Aminoacrylate in a ridA Mutant of *Salmonella enterica*. mSystems.

[158] Borchert A.J., Walejko J.M., Le Guennec A., Ernst D.C., Edison A.S., Downs D.M. (2019). Integrated Metabolomics and Transcriptomics Suggest the Global Metabolic Response to 2-Aminoacrylate Stress in *Salmonella enterica*. Metabolities.

[159] Jibrin M.O., Liu Q., Jones J.B., Zhang S. (2021). Surfactants in plant disease management: A brief review and case studies. Plant Pathol..

[160] Pluskal T., Castillo S., Villar-Briones A., Orešič M. (2010). MZmine 2: Modular framework for processing, visualizing, and analyzing mass spectrometry-based molecular profile data. BMC Bioinform..

[161] Goodacre R., Broadhurst D., Smilde A.K., Kristal B.S., Baker J.D., Beger R., Bessant C., Connor S., Capuani G., Craig A. (2007). Proposed minimum reporting standards for data analysis in metabolomics. Metabolomics.

[162] Salek R.M., Haug K., Steinbeck C. (2013). Dissemination of metabolomics results: Role of MetaboLights and COSMOS. GigaScience.

[163] Chong J., Wishart D.S., Xia J. (2019). Using MetaboAnalyst 4.0 for Comprehensive and Integrative Metabolomics Data Analysis. Curr. Protoc. Bioinform..

[164] Karp P.D., Billington R., Caspi R., Fulcher C.A., Latendresse M., Kothari A., Keseler I.M., Krummenacker M., Midford P.E., Ong Q. (2019). The BioCyc collection of microbial genomes and metabolic pathways. Briefings Bioinform..

