# Supplementary material for: Metabolomics Insights into Chemical Convergence in Xanthomonas perforans and Metabolic Changes Following Treatment with the Small Molecule Carvacrol
Source: Metabolites. 2021 Dec 16;11(12):879. doi: 10.3390/metabo11120879 (PMC8706651; doi:10.3390/metabo11120879)
Supplement: Supplementary file 1 [file metabolites-11-00879-s001.zip › Supplementary File 1-revised.pdf]

Annotation of Metabolites in *Xanthomonas perforans*

The total identified features in the initial study without carvacrol treatment is 1477 in the positive ion mode and 1094 in the negative ion mode (Supplementary Figure S1). The significant peaks as identified by ANOVA ($p < 0.05$) is presented in Supplementary Table 1.2.

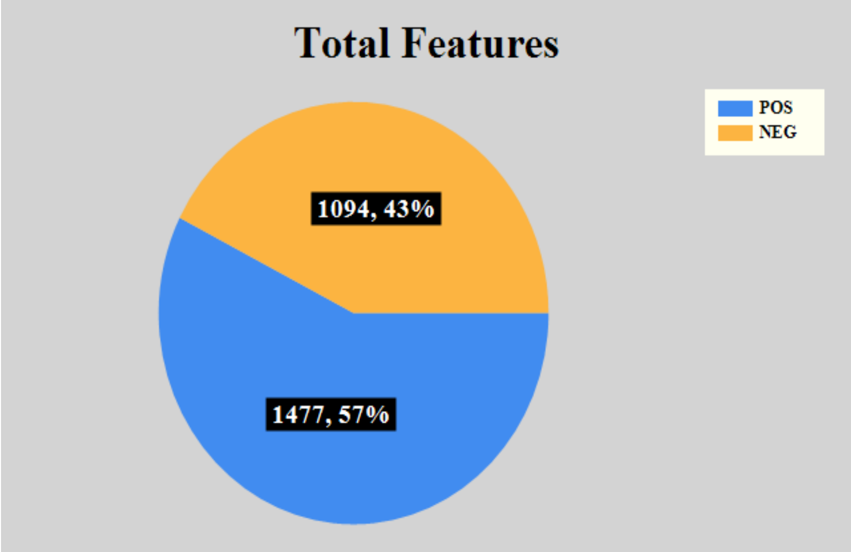

Supplementary Figure S1 Total number of metabolites in positive (POS) and negative (NEG) ion phases in the Xp91-118 carvacrol-treated study

Supplementary Table S1. Significant metabolites (P<0.05) annotated from untargeted metabolomics of carvacrol-treated *Xanthomonas perforans* reference strain 91-118 in the positive phase LC-MS. Unique metabolites identified for the first time in a microbial or xanthomonad system are in bold. Significant peaks were initially searched against SECIM database. The unidentified metabolites after SECIM search (unknown metabolites) were further searched against HMDB and METLIN databases. The annotation level is level 2 based on the chemical analysis working group of the Metabolomics Standards Initiative (MSI). For each metabolite, metabolite name is followed by an underscore followed by the molecular mass, a dash, and the retention time.

**Metabolite (Positive Mode)**	**FDR**	***p*-value**
**SECIM Annotation**		
Sn-glycerol 3-phosphate_173.0210-0.73	2.7916E-27	3.1508E-30
Alpha-aminoadipate/n-methyl-l-glutamate_162.0758-0.91	7.7314E-24	5.2357E-26
Pyridoxal_168.0649-1.94	1.4782E-19	2.1689E-21
Spermidine_146.1649-0.56	4.0805E-17	1.0593E-18
Nepsilon, nepsilon, nepsilon-trimethyllysine_189.1594-0.71	4.9628E-17	1.3443E-18
N-acetyl-hexosamine_244.0786-0.81	9.0358E-17	2.6516E-18
Hypoxanthine_137.0456-2.11	8.8689E-16	3.2032E-17
Leu pro_229.1541-7.19	1.939E-15	7.8784E-17
Anthranilate_120.0443-8.74	8.4898E-15	4.312E-16
Glycyl-L-leucine_189.1230-6.75	2.1202E-13	1.388E-14
Cytidine_244.0922-1.66	5.945E-12	5.7034E-13
Isocytosine_112.0504-1.65	1.008E-11	1.0011E-12
Ethanolamine phosphate_142.0258-0.69	1.0641E-11	1.0809E-12
Nicotinamide_123.0551-1.70	1.8002E-11	1.9912E-12
Guanosine 5'-monophosphate_364.0642-2.01	1.9751E-11	2.2293E-12
Uridine-5-monophosphate_325.0421-1.25	3.2889E-11	3.7863E-12
D-ribose_173.0420-0.78	6.8474E-11	8.1148E-12
Creatine-D3_157.0774-0.91	1.1706E-10	1.4533E-11
Glucosamine/mannosamine_180.0862-0.74	1.3264E-08	2.1408E-09
Putrescine_89.1073-0.60	2.387E-08	4.0142E-09
Uracil_113.0344-1.44	2.6606E-08	4.5045E-09
Carnosine_227.1132-0.71	4.7355E-08	8.5516E-09
Cytosine_112.0504-0.86	1.92E-07	3.7707E-08
Agmatine sulfate_131.1289-0.66	2.6697E-07	5.3936E-08
Glycerophosphocholine_258.1094-0.77	4.4735E-07	9.2904E-08
L-glutamine_147.0761-0.87	6.031E-07	1.2933E-07
L-cystine_241.0307-0.69	1.128E-06	2.5082E-07
L-proline_116.0705-0.86	2.206E-06	5.1042E-07
Aldo/keto-hexose_203.0522-0.73	1.2413E-05	3.1942E-06
L-cysteine_122.0266-0.76	1.4654E-05	3.83E-06

6c-sugar alcohol_205.0678-0.72	2.1934E-05	5.8673E-06
Uridine_267.0581-2.90	2.4197E-05	6.5272E-06
5-aminolevulinic acid_132.0653-0.74	5.0565E-05	1.4896E-05
Urocanate_139.0500-2.02	9.5746E-05	2.907E-05

Supplementary Table S1 (Continued).

**Metabolite (Positive Mode)**	**FDR**	**p-value**
**SECIM Annotation**		
Adenosine 5'-monophosphate_348.0694-1.70	0.0001137	3.4905E-05
L-serine_106.0498-0.70	0.000121	3.7284E-05
4-aminobutanoate gaba_104.0705-0.79	0.00020969	6.6979E-05
Orthophosphate_98.9841-0.88	0.00021828	7.0461E-05
N-alpha-acetyl-l-lysine_189.1229-1.07	0.00054279	0.00019053
D--galactosamine_202.0682-0.67	0.0021838	0.00088152
Alpha-aminoadipate/n-methyl-l-glutamate_162.0758-1.05	0.0032597	0.0013833
5-aminolevulinic acid_132.0654-0.88	0.0032626	0.0013883
L-cysteine_122.0267-0.70	0.0047266	0.0020912
L-asparagine_133.0607-0.71	0.005	0.0022404
Ornithine_133.0969-0.63	0.0051804	0.0023388
Dl-5-hydroxylysine_163.1074-0.63	0.0085508	0.0041403
Nicotinate_124.0392-1.42	0.0085933	0.0041706
L-lysine_147.1125-0.63	0.01338	0.0069017
Citrulline_176.1026-0.78	0.01426	0.0074519
N-acetyl-arginine_217.1291-1.66	0.016184	0.0086397
Pyridoxine_170.0804-2.93	0.022344	0.012811
Histamine_112.0867-0.62	0.024173	0.014187
Pipecolate/l-pipecolic acid_130.0861-1.25	0.024876	0.014656
L-isoleucine_132.1017-2.22	0.025579	0.015209
Creatinine_114.0661-0.94	0.025579	0.015215
Glycine_76.0393-0.70	0.028101	0.016873
Dopamine_154.0859-2.92	0.030192	0.018503
5-oxo-l-proline_130.0497-1.86	0.034851	0.021668
L-cystathionine_223.0744-0.69	0.034851	0.021674
Creatine_132.0766-0.91	0.039409	0.024998
Taurine_126.0219-0.71	0.044435	0.028988
3-methyl-2-oxindole_148.0753-8.79	0.049676	0.03308
L-methionine_150.0581-1.43	0.058479	0.039734
Pyridoxine_192.0627-2.88	0.058949	0.04012
L-carnitine_162.1121-0.97	0.059693	0.040694
N-acetylputrescine_131.1176-1.13	0.059786	0.040824

L-kynurenine_209.0915-6.43	0.06655	0.046022

Supplementary Table S1 (Continued).

**Metabolite (Positive Mode)**	**FDR**	**p-value**
**HMDB/METLIN Annotation**		
**Sarmentosin_276.1071-6.30**	**2.79E-27**	**3.15E-30**
N6,N6-dimethyladenosine_296.1344-7.38	7.66E-25	2.59E-27
**Pyrocoll_187.0494-6.30**	**7.73E-24**	**4.56E-26**
N2-acetyl-l-aminoadipate_204.0863-0.82	2.89E-22	2.61E-24
Cytosine_204.0862-6.92	1.11E-15	4.37E-17
N-carbamoylputrescine_132.1130-1.47	5.84E-14	3.43E-15
Valyvaline_217.1542-5.97	1.06E-12	8.41E-14
**Harman_183.0915-8.80**	**5.52E-12**	**5.21E-13**
**Piperidine_86.0964-1.07**	**6.44E-10**	**8.73E-11**
**Norharman_157.1082-1.35**	**9.18E-10**	**1.27E-10**
**Beta-carboline_169.0759-8.54**	**1.78E-09**	**2.53E-10**
Oxoarginine_174.0872-0.97	9.08E-08	1.73E-08
Hydroxypentobarbital_243.1334-8.19	2.03E-07	4.02E-08
5-nitro-ortho-anisidine_169.0602-1.70	3.89E-07	1.71E-06
8-hydroxylanylclavam_217.0814-1.48	5.43E-06	2.05E-05
**Enterodiol sulfate_383.1149-0.73**	**3.11E-05**	**8.64E-06**
**(2s,4s)-monatin_293.1127-7.66**	**3.33E-05**	**9.31E-06**
Prenyl-l-cysteine_190.0901-0.91	4.85E-05	1.41E-05
Leu-gly-pro_286.1757-7.57	0.000216	6.93E-05
8-hydroxypurine_139.0612-2.11	0.000218	7.04E-05
N-lactoyl-tryptophan_277.1177-8.65	0.000288	9.53E-05
Ozagrel_229.0963-8.31	0.000344	0.000117
Ethirimol_210.1597-7.12	0.001004	0.000376
**Maculosin_261.1225-7.78**	**0.001276**	**0.000485**
Ampicillin_350.1181-1.22	0.001706	0.000664
2-oxoarginine_174.0870-1.11	0.002284	0.000931
Tulobuterol_228.1139-11.67	0.002858	0.001183
Pyroglutamylglycine_187.0708-1.13	0.004322	0.001903
Sulpho nonoate_142.9764-0.77	0.00916	0.004487
Deoxycytidine_228.0973-1.51	0.010255	0.005104
Pyroglutamylglycine_187.0709-1.59	0.010863	0.005493
L-formylkynurenine_237.0863-3.95	0.011078	0.005614
Isocarbamid_186.1232-0.91	0.011955	0.006086
Zalcitabine_212.1025-3.70	0.012503	0.006392
Metharbital_199.1072-5.85	0.016213	0.008674

Ectoine_143.0813-3.27	0.016308	0.008743
Aprobarbital_211.1070-6.13	0.016656	0.008967
Barbital_185.0915-2.57	0.020804	0.011599
Etomidate_245.1275-8.70	0.020858	0.011677

Supplementary Table S1 (Continued).

**Metabolite (Positive Mode)**	**FDR**	**p-value**
**HMDB/METLIN Annotation**		
Homocitrulline_190.1181-0.82	0.020978	0.011767
**Methoxybrassinin_267.0621-3.47**	**0.021912**	**0.01244**
Pyroglutmylglycine_187.0709-1.75	0.022412	0.012901
Biotin_245.0949-6.24	0.022911	0.01324
5-aminopentanamide_117.1021-0.81	0.023288	0.013536
Lyxosylamine_150.0760-0.71	0.024173	0.014186
Pirbuterol_241.1541-6.19	0.053326	0.036077
Heptanoylcholine_216.1953-10.20	0.064041	0.043874
Orotic acid_157.0241-1.22	0.066898	0.046436

Supplementary Table S2. Significant metabolites (P<0.05) annotated from untargeted metabolomics of *Xanthomonas perforans* reference strain 91-118 in the negative phase LC-MS. Unique metabolites identified for the first time in a microbial or xanthomonad system are in bold. Significant peaks were initially searched against SECIM database. The unidentified metabolites after SECIM search (unknown metabolites) were further searched against HMDB and METLIN databases. The annotation level is level 2 based on the chemical analysis working group of the Metabolomics Standards Initiative (MSI). For each metabolite, metabolite name is followed by an underscore followed by the molecular mass, a dash, and the retention time.

Metabolite (negative mode)	FDR	p-value
**SECIM Annotation**		
N-methyl-l-glutamate_160.0624-0.91	2.9343E-24	8.9462E-27
Glycerol 2-phosphate/sn-glycerol 3-phosphate_171.0064-0.71	7.8672E-24	3.8632E-26
Pantetheine_277.1228-8.01	9.2761E-21	7.0702E-23
Malate_133.0149-1.13	1.1837E-15	2.7065E-17
2-aminoethyl dihydrogen phosphate_140.0121-0.70	1.4002E-15	3.6287E-17
N-acetyl-hexosamine_220.0831-0.82	4.0311E-15	1.3519E-16
Uridine-5-monophosphate_323.0292-1.26	4.0268E-14	1.6574E-15
N-acetyl-d-mannosamine_256.0600-0.81	7.2005E-14	3.0734E-15
Xanthine_151.0264-2.59	8.4553E-14	3.895E-15
Uracil_111.0202-1.42	8.4553E-14	3.9957E-15
Aldopentose_149.0470-0.79	1.371E-13	6.8966E-15
Hypoxanthine_135.0315-2.13	8.3636E-13	4.9723E-14
L-cysteine-s-sulfate_199.9696-0.75	5.2772E-12	4.344E-13
Cmp_322.0451-1.07	1.1542E-11	1.0557E-12
D-glucuronic acid/d-glucuronolactone/d--galacturonic acid_193.0359-0.78	2.1825E-11	2.2291E-12
Cytidine_242.0787-1.65	2.6934E-10	3.2436E-11
L-glutamine_145.0622-0.73	3.612E-10	4.4049E-11
Guanosine 5'-monophosphate_362.0515-2.02	6.606E-10	8.9625E-11
L-histidine_154.0626-0.69	1.0128E-09	1.4821E-10
5-aminolevulinic acid_130.0513-0.74	1.4427E-09	2.1772E-10
Uridine_243.0627-2.91	1.7721E-09	2.8095E-10
L-serine_104.0354-0.69	2.3794E-09	3.8811E-10
1,2-Benzisothiazolin-3-one_150.0022-10.07	5.2743E-09	9.4874E-10
D-ribose 5-phosphate/ribulose 5-phosphate_229.0124-0.73	8.0711E-09	1.4887E-09
$C_6H_{12}O_6$-hexose/ketose/inositol_179.0566-0.75	9.0725E-09	1.7426E-09
N-acetyl-l-aspartic acid_174.0416-1.71	5.3133E-08	1.1582E-08
3-2-hydroxyphenylpropanoate_165.0560-8.64	8.0539E-08	1.8048E-08

Urocanate_137.0359-2.03	2.2001E-07	5.2654E-08
Ll-2,6-diaminoheptanedioate_189.0898-0.85	2.7276E-07	6.6527E-08
Alpha-aminoadipate_160.0619-1.06	3.3311E-06	9.3435E-07
Citrulline_174.0864-0.78	3.5314E-05	1.1305E-05
Asparagine_131.0466-0.71	5.2996E-05	1.7207E-05
Adenosine 5'-monophosphate_346.0564-1.72	6.9942E-05	2.3349E-05

Supplementary Table S2 (Continued).

**Metabolite (negative mode)**	**FDR**	**p-value**
**SECIM Annotation**		
L-kynurenine_207.0779-6.43	0.00020696	7.3508E-05
Carnosine_225.0999-0.71	0.00031548	0.00011542
Pyridoxine_168.0669-2.93	0.00049281	0.00018781
2,3-dihydroxyisovalerate_133.0514-1.43	0.0011866	0.00048477
4-oxoproline_128.0356-1.86	0.0015953	0.00067606
Glucose/fructose_217.0307-0.72	0.0022663	0.00098712
Dl-5-hydroxylysine_161.0936-0.63	0.0023271	0.0010359
Proline_114.0562-0.85	0.0034356	0.0016235
Pantothenic acid_218.1037-6.59	0.0036309	0.0017269
Succinate_117.0195-2.31	0.0038121	0.0018189
3-sulfino-l-alanine_152.0027-0.77	0.0062424	0.0031593
Glucose/fructose_215.0333-0.73	0.0064674	0.0033027
L-methionine_148.0428-1.43	0.011411	0.0062272
Citramalate_147.0308-2.27	0.0182	0.010431
Taurine_124.0075-0.69	0.018616	0.010699
N-acetyl-l-alanine_130.0513-2.29	0.018833	0.010854
C12H22O11-disaccharide-6C _341.1095-0.89	0.018833	0.010881
Indole-3-acetate_174.0583-9.27	0.018945	0.011003
L-cystathionine_221.0606-0.85	0.022196	0.013162
Xylenesulfonate_185.0281-8.21	0.027803	0.016741
Glutarate_131.0353-4.77	0.038273	0.023581
L-cysteic acid_167.9975-0.72	0.052222	0.033116
**HMDB/METLIN Annotation**		
**Sarmentosin_274.0923-6.30**	**3.56E-25**	**5.42E-28**
Sulfate_96.9600-0.91	1.54E-13	7.99E-15
Sulfonic acid_334.0762-0.84	9.47E-13	5.77E-14
Uridine_243.0626-1.52	5.21E-10	6.59E-11
8-Hydroxypurine_137.0472-2.13	1.52E-09	2.32E-10
Desacetylcefotaxime_412.0401-1.06	8.89E-09	1.69E-09
Glycylprolylhydroxyproline_284.1258-3.09	9.64E-09	1.88E-09

Carboxylic acid_265.0195-2.57	1.69E-08	3.34E-09
L-Ornithine_302.1365-7.13	7.29E-07	1.88E-07
N-histidine_316.1156-1.61	8.90E-07	2.31E-07
Glycerol_245.0435-0.75	1.19E-06	3.17E-07
Glycerophosphoinositol_333.0584-2.92	6.28E-06	1.81E-06
Hydroxyaminaline_126.0199-1.09	1.03E-05	3.03E-06
2-Oxoarginine_172.0727-1.12	9.05E-05	3.08E-05
Penmacric acid_201.0526-0.79	9.57E-05	3.27E-05
Propanoic acid_275.1043-8.64	0.00028	0.000101
**Saccharopine_275.1260-0.76**	**0.000283**	**0.000103**

Supplementary Table S2 (Continued).

**Metabolite (negative mode)**	**FDR**	**p-value**
**HMDB/METLIN Annotation**		
p-Toluenesulfonic acid_171.0126-7.17	0.000408	0.000154
Pyroglutamylglycine_185.0577-1.64	0.000753	0.000295
N-Nonanoylglycine_214.1453-9.28	0.00093	0.000368
Phenylalanyl_261.0885-8.00	0.001006	0.000405
Dimethoxyquinazoline_219.0880-7.30	0.001873	0.000802
**Cyclobrassinone_231.0245-0.76**	**0.002399**	**0.001071**
Trihydroxyoxane-2-carboxylic acid_305.0636-1.49	0.002433	0.001098
7-oxabicyclo[4.1.0]hept-3-ene-2,5-dione_123.0089-1.19	0.004355	0.002105
Purine_119.0363-1.26	0.004829	0.002363
**beta-carboline-3-carboxylic acid_215.0830-8.22**	**0.008274**	**0.004351**
R-138727(Prasugrel)_348.1060-1.16	0.015033	0.008359
Potassium 2-(1'-ethoxy) ethoxypropanoate_199.0375-0.84	0.027385	0.016406
Uridine 2',3'-cyclic phosphate_305.0191-2.31	0.031861	0.01933
Propanoic acid_275.1036-8.03	0.057912	0.037342
