# Supplementary material for: Metabolomics Insights into Chemical Convergence in Xanthomonas perforans and Metabolic Changes Following Treatment with the Small Molecule Carvacrol
Source: Metabolites. 2021 Dec 16;11(12):879. doi: 10.3390/metabo11120879 (PMC8706651; doi:10.3390/metabo11120879)
Supplement: Supplementary file 1 [file metabolites-11-00879-s001.zip › Supplementary File 2-revised.pdf]

Pathway analyses

A. **Pathway Analyses on BioCyc** (www.biocyc.com, Karp *et al.* 2019; Paley and Karp, 2021).

Pathway coverage analyses were carried out on BioCyc using the metabolomics pathway coverage tool.. Here, the annotation for each of the significant pathways in Xp91-118 in both positive and negative ion phases are presented. Annotation of gene loci in Xp91-118 are also available on BioCyc (www.biocyc.org).

a. Positive ion phase: Metabolic pathway coverage report identified of 18 pathways for metabolites in the positive phase. The table below shows the pathways and metabolites.

Supplementary Table S3. Positive Phase Pathways and metabolites covered in each pathway. Only the underlined compounds are the significant metabolites from our metabolomics annotation.

Pathways	Covered Compounds
adenosylcobalamin salvage from cobinamide I	GMP<br>phosphate<br>nicotinate
biotin-carboxyl carrier protein assembly	phosphate<br>biotin<br>AMP
L-arginine biosynthesis I (via L-ornithine)	L-ornithine<br>phosphate<br>L-glutamine<br>AMP
L-asparagine biosynthesis II	L-asparagine<br>AMP
L-cysteine biosynthesis VI (from L-methionine)	L-cysteine<br>L-cystathionine<br>L-serine<br>L-methionine<br>phosphate
L-histidine degradation II	urocanate
L-lysine biosynthesis I	phosphate<br>L-lysine
L-tryptophan degradation I (via anthranilate)	L-kynurenine<br>anthranilate
proline to cytochrome bo oxidase electron transfer	L-proline
putrescine degradation II	putrescine<br>phosphate
pyridoxal 5'-phosphate salvage I	pyridoxine
pyrimidine ribonucleosides salvage II	uridine<br>uracil<br>cytidine
superpathway of L-isoleucine biosynthesis I	L-isoleucine<br>phosphate
superpathway of L-serine and glycine biosynthesis I	L-serine<br>glycine<br>phosphate

Supplementary Table S3 (continued).

**Pathways**	**Covered Compounds**
superpathway of pyrimidine deoxyribonucleoside salvage	2'-deoxycytidine
superpathway of pyrimidine ribonucleotides de novo biosynthesis	L-glutamine<br>phosphate<br>UMP<br>orotate
taurine degradation IV	taurine
tetrapyrrole biosynthesis I (from glutamate)	AMP<br>5-aminolevulinate

b. Negative ion phase: A pathway covering set of 48 pathways covers significant metabolites in the negative phase. The table below shows the pathways and metabolites in each pathway. While 24 metabolites originally cover pathways of Xp91-118, the different forms of the metabolites are also included.

Supplementary Table S4. Negative Phase Pathways and metabolites covered in each pathway. Only the underlined compounds are the significant metabolites from our metabolomics annotation.

**Pathways**	**Covered Compounds**
2-oxopentenoate degradation	(S)-4-hydroxy-2-oxopentanoate<br>pyruvate<br>2-oxopent-4-enoate
biotin biosynthesis I	S-adenosyl-L-methionine<br>S-adenosyl-4-methylthio-2-oxobutanoate<br>L-methionine<br>L-alanine<br>S-adenosyl-L-homocysteine
CMP-2-deoxy-D-manno-octulosonate biosynthesis	phosphoenolpyruvate<br>2-deoxy-α-D-manno-2-octulosonate
D-galactonate degradation	D-galactonate<br>2-dehydro-2-deoxy-D-galactonate<br>pyruvate
glycerol and glycerophosphodiester degradation	glycerol
glycine betaine biosynthesis I (Gram-negative bacteria)	glycine betaine
glycolysis III (from glucose)	2-phospho-D-glycerate<br>2-phospho-D-glycerate<br>pyruvate<br>phospho*enol*pyruvate<br>2-phospho-D-glyceroyl-phosphate<br>2,2-diphospho-D-glycerate
glyoxylate cycle	(S)-malate<br>oxaloacetate<br>*cis*-aconitate<br>D-*threo*-isocitrate<br>glyoxylate<br>succinate
indole-2-acetate biosynthesis V (bacteria and fungi)	(indol-2-yl)acetate

Supplementary Table S4 (continued).

**Pathways**	**Covered Compounds**
L-arginine biosynthesis I (via L-ornithine)	N-acetyl-L-ornithine<br>L-ornithine<br>acetate<br>2-oxoglutarate<br>L-glutamate<br>*N*-acetyl-L-glutamate<br>L-glutamine<br>L-citrulline<br>L-arginine<br>fumarate<br>L-aspartate<br>AMP
L-asparagine degradation I	L-asparagine<br>L-aspartate
L-cysteine biosynthesis VI (from L-methionine)	L-cysteine<br>O-succinyl-L-homoserine<br>L-cystathionine<br>succinate<br>L-homocysteine<br>L-serine<br>*S*-adenosyl-L-methionine<br>*S*-adenosyl-L-homocysteine<br>L-methionine<br>S-ribosyl-L-homocysteine
L-histidine degradation II	N-formyl-L-glutamate<br>L-glutamate<br>formate<br>*N*-formimino-L-glutamate<br>urocanate<br>L-histidine

Supplementary Table S4 (continued).

**Pathways**	**Covered Compounds**
L-lysine biosynthesis I	L-aspartate<br>L-aspartyl-4-phosphate<br>L-aspartate-semialdehyde<br>pyruvate<br>2-oxoglutarate<br>L-glutamate<br>L,L-diaminopimelate<br>succinate<br>*meso*-diaminopimelate<br>L-lysine
L-lysine degradation VI	L-2-aminoadipate<br>L-lysine<br>2-oxoglutarate<br>L-glutamate
L-phenylalanine biosynthesis I	chorismate<br>*keto*-phenylpyruvate<br>L-glutamate<br>L-phenylalanine<br>2-oxoglutarate
L-proline biosynthesis I	L-glutamate<br>L-glutamate-5-semialdehyde<br>L-proline<br>(S)-1-pyrroline-5-carboxylate
L-threonine degradation II	glycine<br>L-2-amino-2-oxobutanoate<br>L-threonine
L-tryptophan degradation I (via anthranilate)	L-tryptophan<br>L-kynurenine<br>formate<br>L-alanine

Supplementary Table S4 (continued).

**Pathways**	**Covered Compounds**
L-tyrosine degradation I	2-oxoglutarate<br>L-tyrosine<br>4-hydroxyphenylpyruvate<br>L-glutamate<br>fumarate<br><br>acetoacetate
L-valine degradation I	(S)-2-amino-2-methylpropanoate<br>2-oxoglutarate<br>L-glutamate<br>2-methyl-2-oxobutanoate<br><br>L-valine
levulinate degradation	4-hydroxypentanoate<br>AMP<br>4-oxopentanoate
methylglyoxal degradation I	(R)-lactate<br><br>pyruvate
octane oxidation	octanoate<br>AMP
palmitate biosynthesis II (bacteria and plants)	laurate<br>AMP<br>palmitate
palmitoleate biosynthesis I (from (5Z)-dodec-5-enoate)	palmitoleate
phosphopantothenate biosynthesis I	2-methyl-2-oxobutanoate<br>(R)-pantoate<br>β-alanine<br>AMP

Supplementary Table S4 (continued).

Pathways	Covered Compounds
protocatechuate degradation I (meta-cleavage pathway)	2-hydroxy-4-oxobutane-1,2,4-tricarboxylate<br><br>pyruvate<br>oxaloacetate<br>(1*E*)-4-oxobut-1-ene-1,2,4-tricarboxylate<br>(1*E*,2*E*)-4-hydroxybuta-1,2-diene-1,2,4-tricarboxylate<br><br>protocatechuate
protocatechuate degradation II (ortho-cleavage pathway)	2-oxoadipate<br>2-carboxy-*cis*,*cis*-muconate<br>protocatechuate
purine nucleotides degradation II (aerobic)	xanthine<br><br>GMP<br>AMP<br>hypoxanthine
purine ribonucleosides degradation	D-ribose 5-phosphate<br><br>hypoxanthine<br><br>xanthine
pyridoxal 5'-phosphate salvage I	pyridoxine
pyrimidine nucleobases salvage I	UMP<br>uracil
S-methyl-5-thio-α-D-ribose 1-phosphate degradation	2-oxoglutaramate<br>2-oxoglutarate<br>L-methionine<br>4-(methylsulfanyl)-2-oxobutanoate<br>L-glutamine<br>formate
sorbitol biosynthesis II	D-gluconate<br>keto-D-fructose

Supplementary Table S4 (continued).

**Pathways**	**Covered Compounds**
stearate biosynthesis II (bacteria and plants)	stearate<br>AMP
superpathway of β-D-glucuronosides degradation	aldehydo-D-glucuronate<br>pyruvate<br>2-dehydro-2-deoxy-D-gluconate<br><br>D-mannonate
superpathway of branched chain amino acid biosynthesis	2-oxobutanoate<br>L-threonine<br>pyruvate<br>(*S*)-2-aceto-2-hydroxybutanoate<br><br>2-oxoglutarate<br>L-isoleucine<br>L-glutamate<br>(*S*)-2-methyl-2-oxopentanoate<br><br>(*S*)-2-acetolactate<br><br>(2*R*)-2,2-dihydroxy-2-methylbutanoate<br>L-valine<br>2-methyl-2-oxobutanoate<br>4-methyl-2-oxopentanoate<br>L-leucine
superpathway of L-isoleucine biosynthesis I	2-oxobutanoate<br>L-threonine<br>pyruvate<br>(*S*)-2-aceto-2-hydroxybutanoate<br>2-oxoglutarate<br>L-isoleucine<br>L-glutamate<br>(*S*)-2-methyl-2-oxopentanoate<br><br>O-phospho-L-homoserine<br><br>L-homoserine<br>L-aspartate-semialdehyde<br>L-aspartyl-4-phosphate<br>L-aspartate, oxaloacetate

Supplementary Table S4 (continued).

**Pathways**	**Covered Compounds**
superpathway of L-methionine biosynthesis (by sulfhydrylation)	sulfate<br>L-homocysteine<br>L-methionine<br>L-homoserine<br>*O*-acetyl-L-homoserine<br><br>acetate<br>L-aspartate-semialdehyde<br>L-aspartyl-4-phosphate<br>L-aspartate<br>2-oxoglutarate<br><br>oxaloacetate<br>L-glutamate
superpathway of pyrimidine deoxyribonucleotides de novo biosynthesis	L-glutamine<br>L-glutamate<br><br>UMP<br><br>orotate<br>*N*-carbamoyl-L-aspartate<br>L-aspartate
superpathway of sulfate assimilation and cysteine biosynthesis	2-phospho-L-serine<br>L-serine<br>2-phospho-D-glycerate<br>2-oxoglutarate<br>L-glutamate<br>*O*-acetyl-L-serine<br>L-cysteine<br>acetate<br>sulfate

Supplementary Table S4 (continued).

**Pathways**	**Covered Compounds**
superpathway of tetrahydrofolate biosynthesis	4-aminobenzoate<br>pyruvate<br>L-glutamine<br>chorismate<br>L-glutamate<br><br>formate<br>AMP
taurine degradation IV	2-oxoglutarate<br>taurine<br>succinate
tetrapyrrole biosynthesis I (from glutamate)	L-glutamate<br>AMP<br>5-aminolevulinate
trehalose degradation VI (periplasmic)	α-D-glucopyranose<br><br>β-D-glucopyranose
UDP-N-acetylmuramoyl-pentapeptide biosynthesis I (meso-diaminopimelate containing)	D-alanine<br>*meso*-diaminopimelate<br>L-glutamate<br>D-glutamate<br>L-alanine<br>phosphoenolpyruvate
UTP and CTP dephosphorylation I	CMP<br>UMP<br>cytidine<br>uridine<br>L-glutamine<br>L-glutamate

B. Pathway Analyses on MetaboAnalyst

The additional pathways identified in *Pseudomonas putida* KT2440 using annotated metabolites in our study are shown in the figures below (Please see figure 3 in main paper for additional details).

Aminoacyl-tRNA biosynthesis

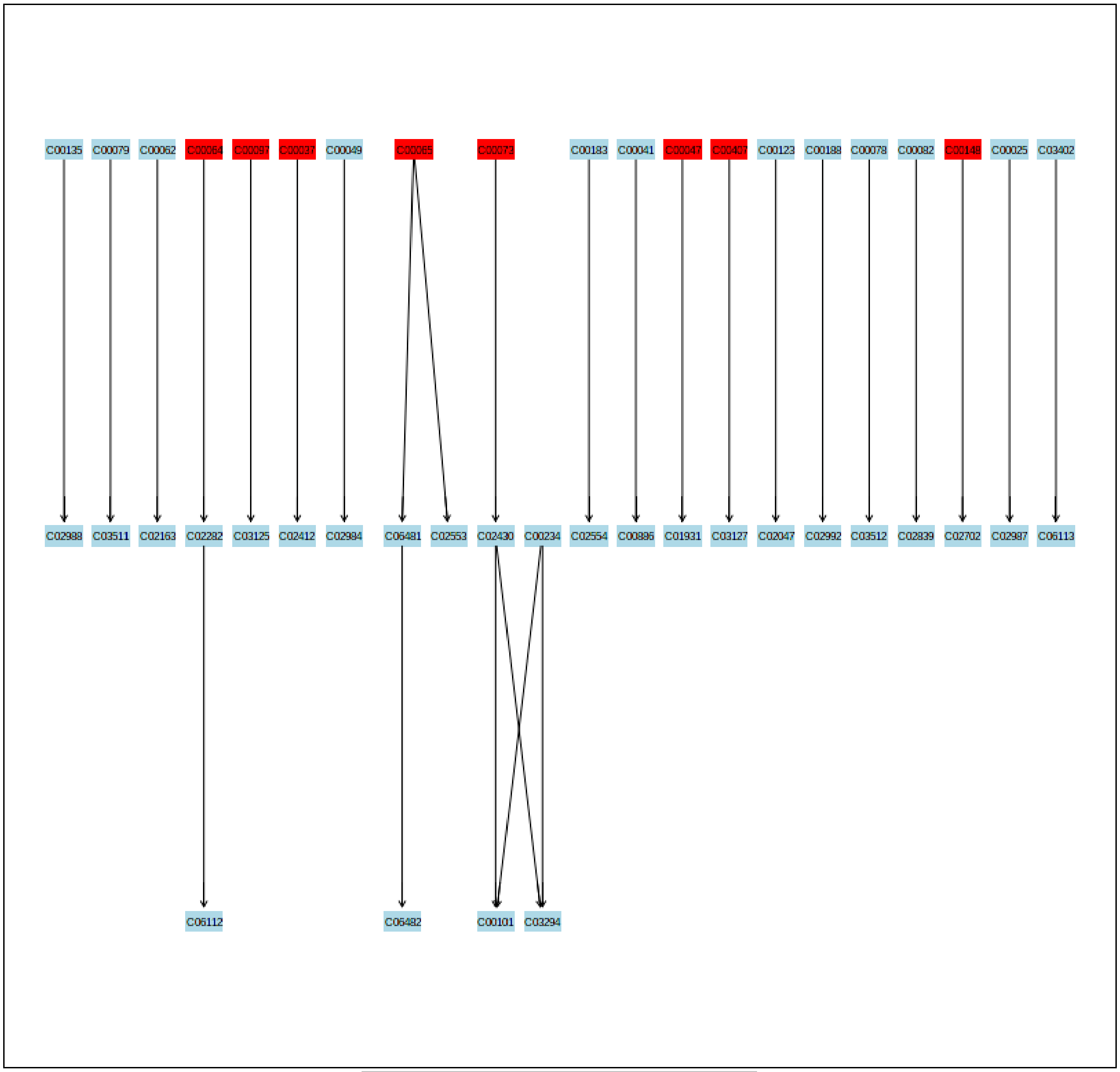

C00064: L-Glutamine; C00097: L-Cysteine; C00027: Glycine; C00065: L-Serine; C00072: L-Methionine; C00047: Lysine; C00407: Isoleucine; C00148: L-Proline

Supplementary Figure S2a(i). Metabolites putatively identified in *X. perforans* that are involved in aminoacyl tRNA biosynthesis

Pyrimidine metabolism

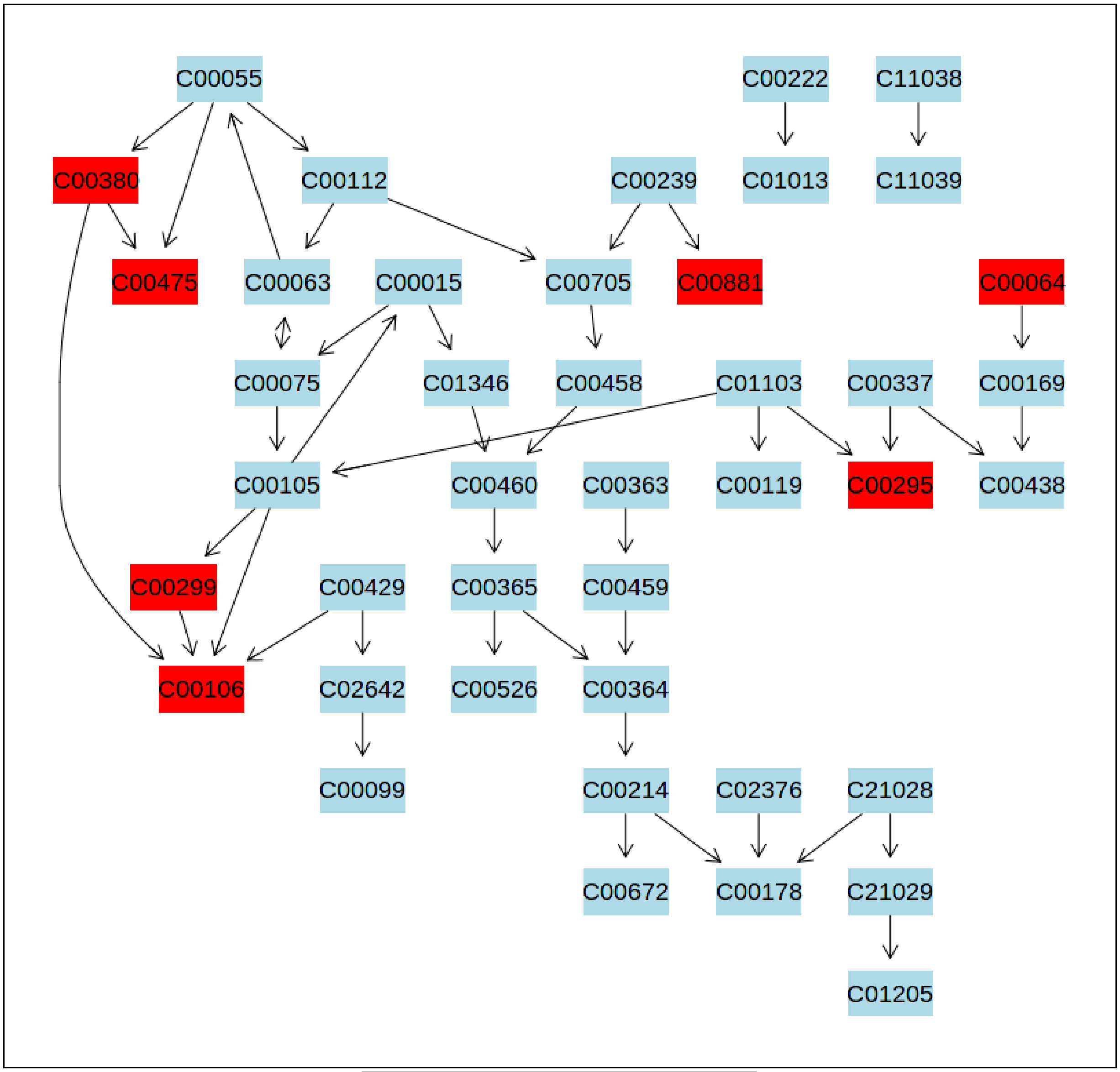

C00280: Cytosine, C00475: Cytidine; C00881: Deoxycitidne; C00064: L-Glutamine; C00295: Orotate; C00299: Uridine, C00106: Uracil

Supplementary Figure S2a(ii). Metabolites putatively identified in *X. perforans* that are involved in pyrimidine metabolism

Glycine, serine and threonine metabolism

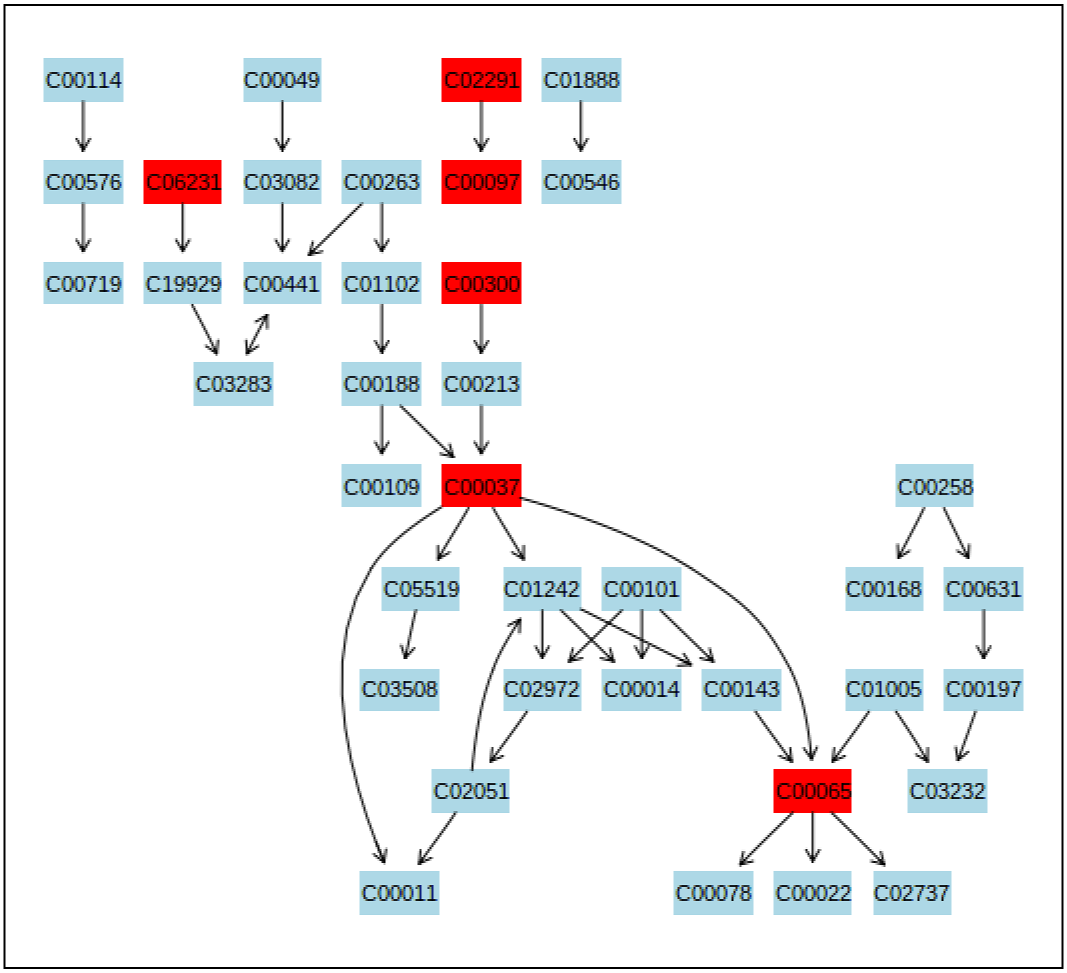

C02291: L-cystathionine, C06221: Ectoine, C00097: Cysteine, C00200: Creatine, C00065: L-Serine

Supplementary Figure S2a(iii). Metabolites putatively identified in *X. perforans* that are involved in glycine, serine and threonine metabolism

Cyanoamino acid metabolism

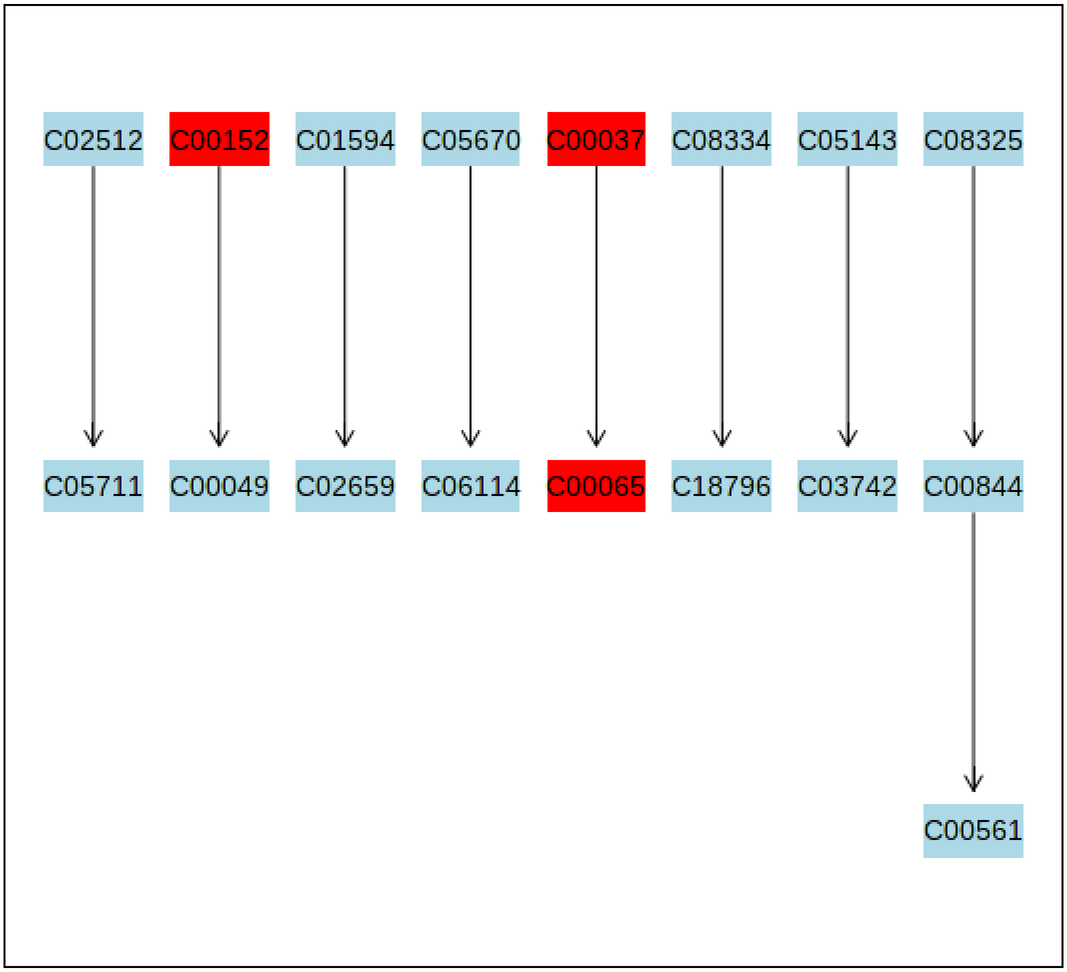

C00152: L-Asparagine, C00027: Glycine, C00065: L-serine

Supplementary Figure S2a(iv). Metabolites putatively identified in *X. perforans* that are involved in cyanoamino acid metabolism

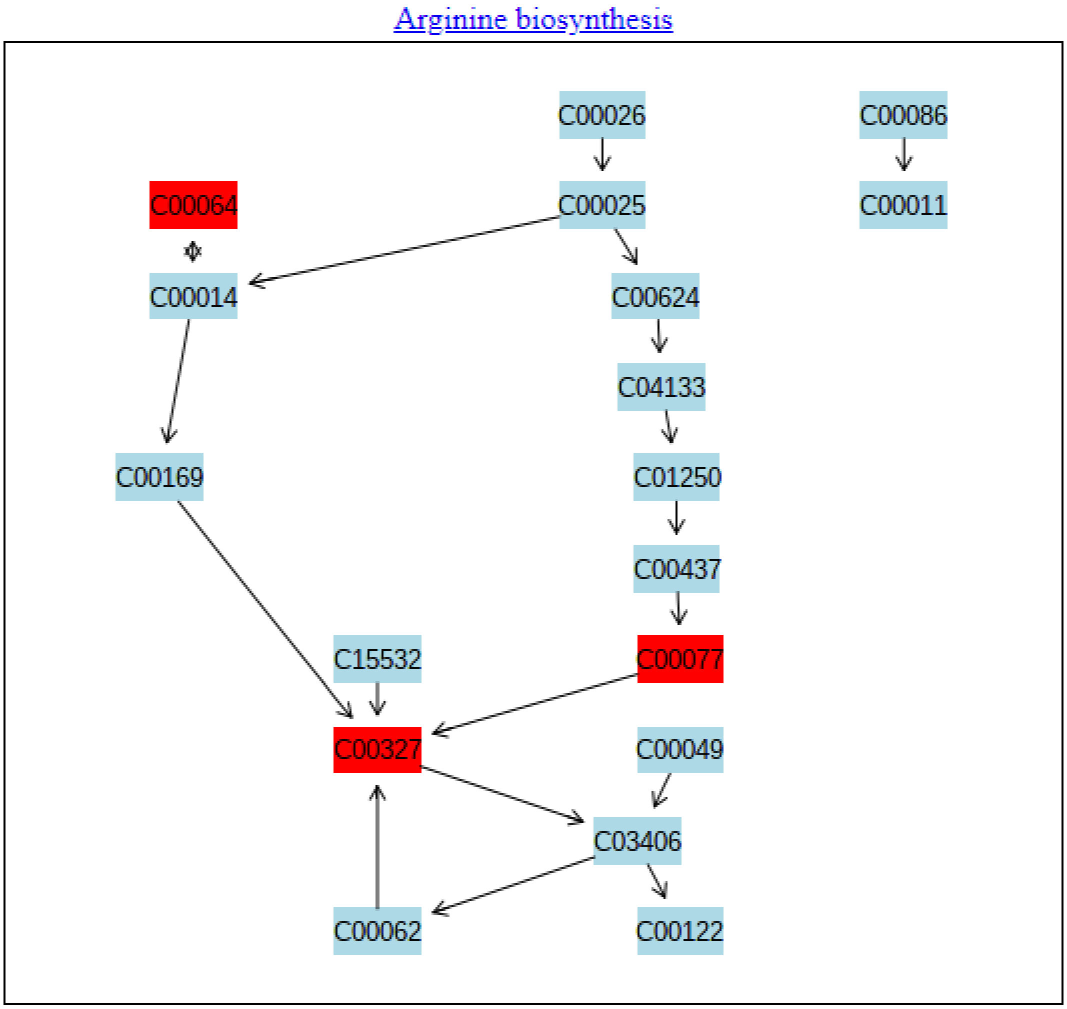

C0064: L-Glutamine, C00227: L-Citrulline, C00077: L-Ornithine

Supplementary Figure S2a (v). Metabolites putatively identified in *X. perforans* that are involved in arginine biosynthesis

Cysteine and methionine metabolism

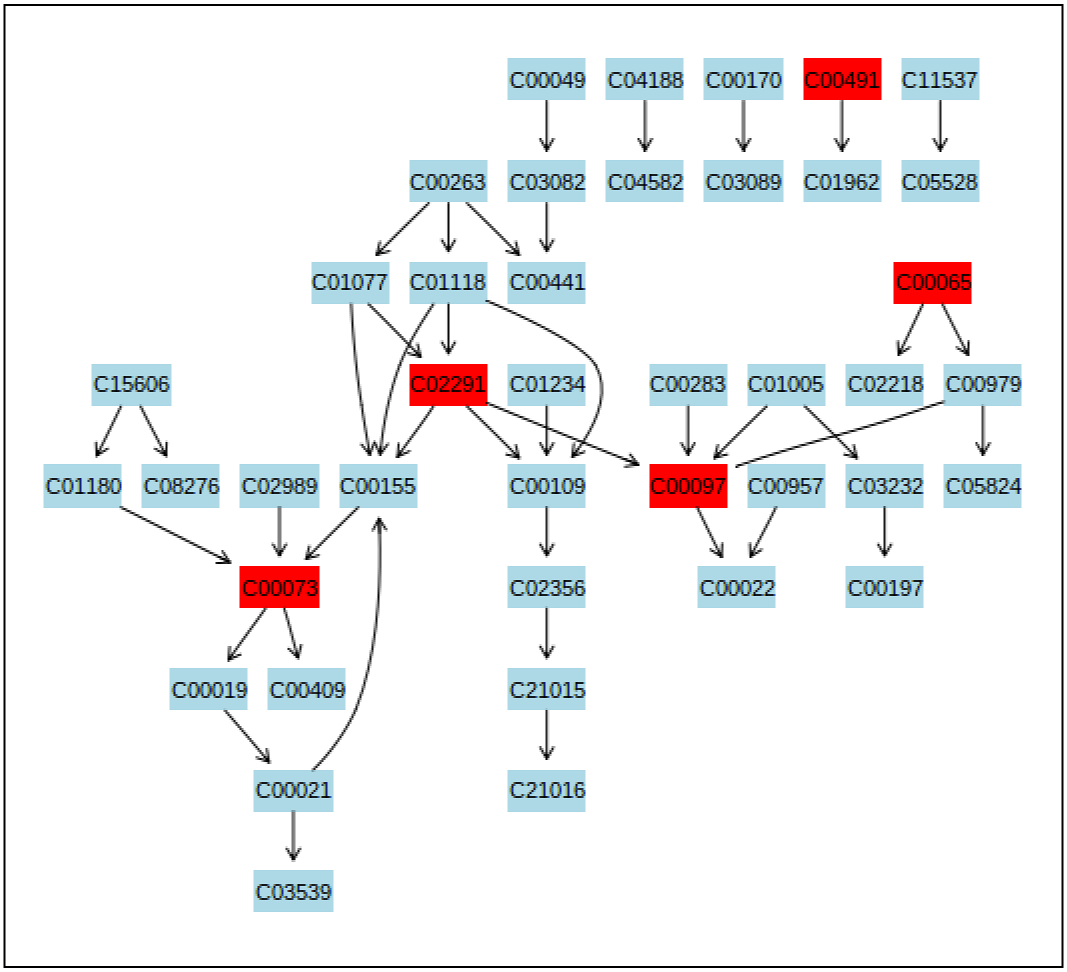

C00491: L-Cystine, C00065: L-Serine, C02291: L-Cystathionine, C00097: L-Cysteine, C00072: L-Methionine

Supplementary Figure S2a (vi). Metabolites putatively identified in *X. perforans* that are involved in cysteine and methionine metabolism

Sulfur metabolism

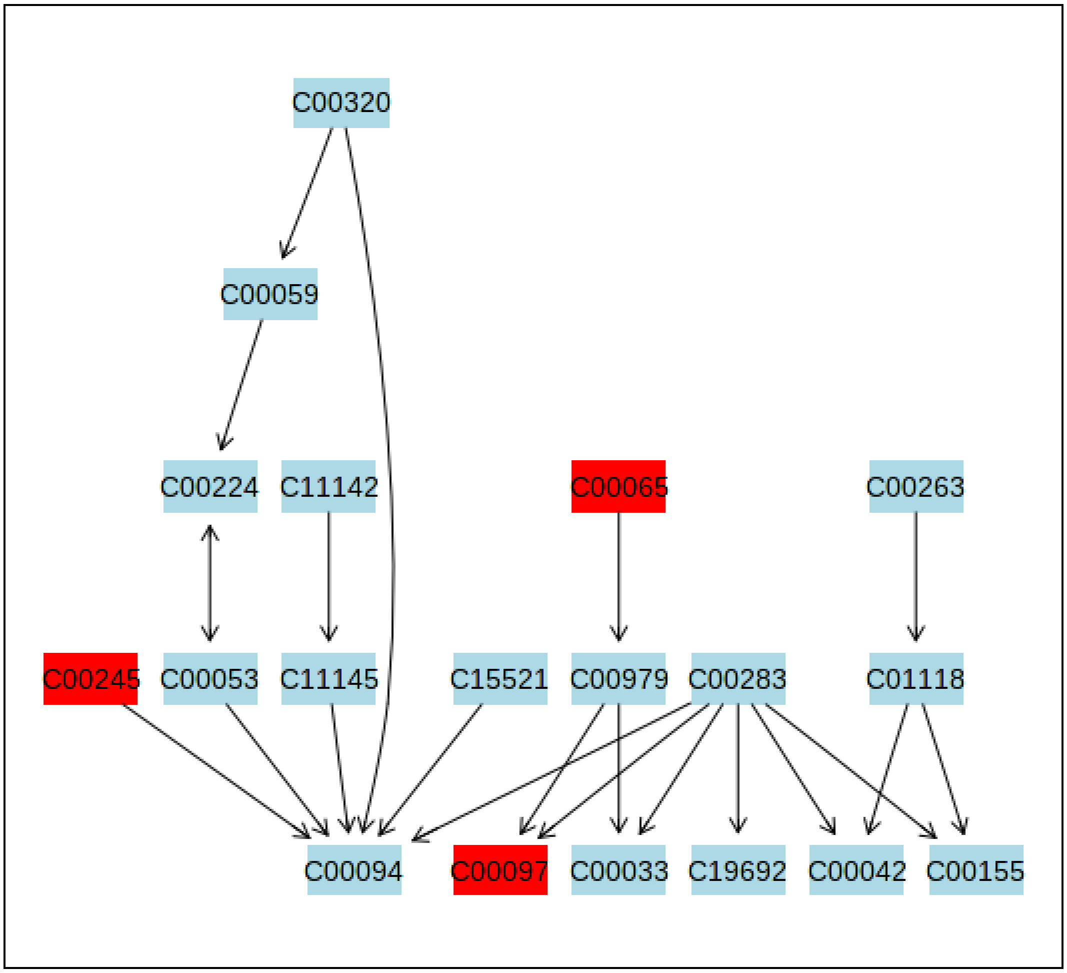

C00065: L-Serine, C00245: Taurine, C00097: L-Cysteine.

Supplementary Figure S2a(vii). Metabolites putatively identified in *X. perforans* that are involved in sulfur metabolism

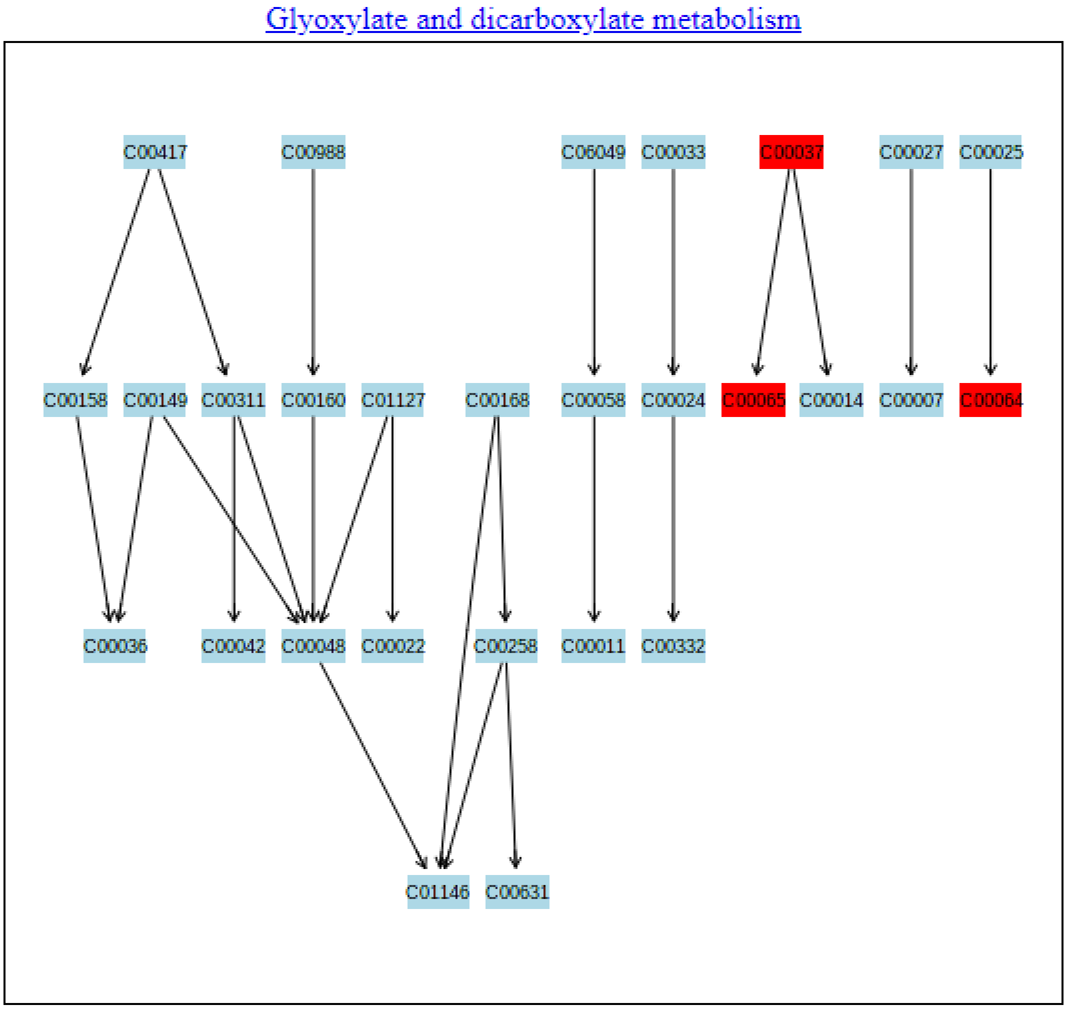

C00027: Glycine, C00065: L-Serine, C00064: L-Glutamine.

Supplementary Figure S2a(viii). Metabolites putatively identified in *X. perforans* that are involved in glyoxylate and dicarboxylate metabolism

Lysine degradation

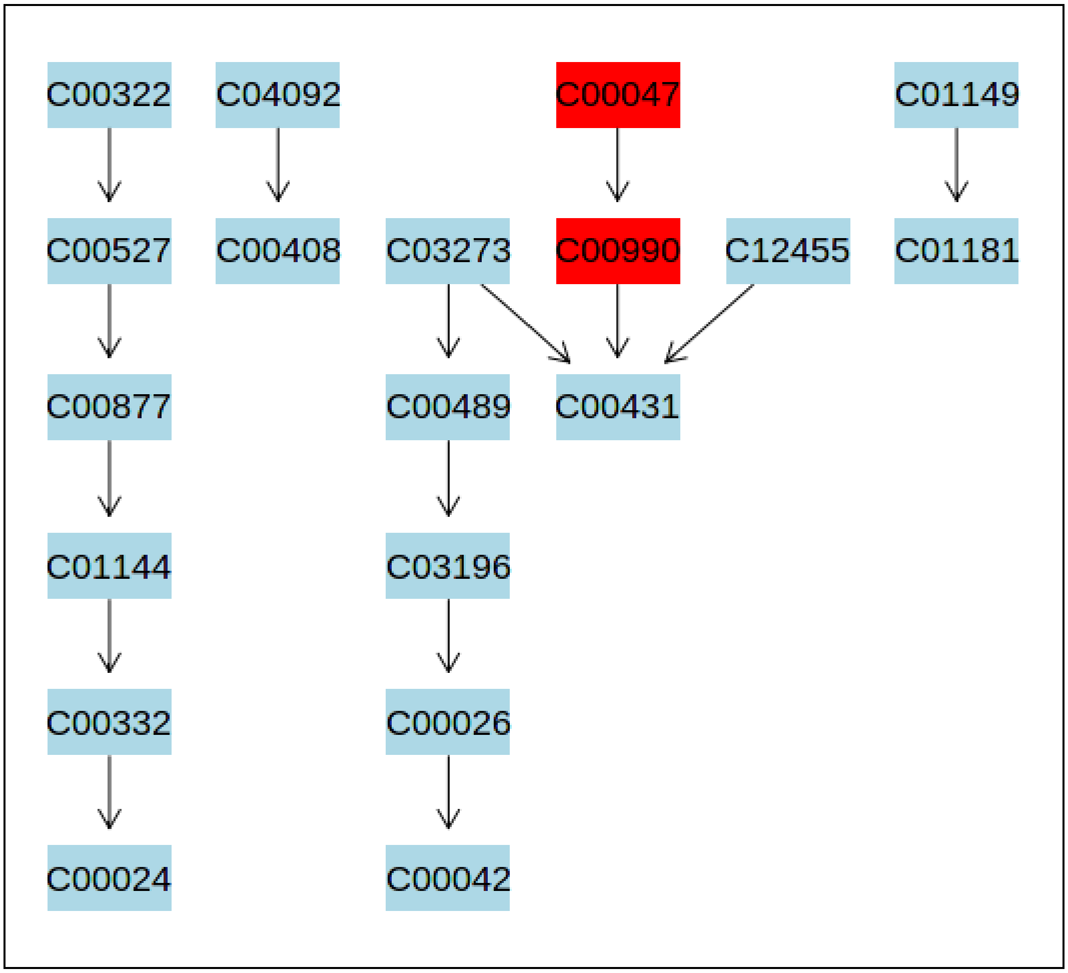

C00047: L-Lysine, C00990: 5-Aminopentanamide.

Supplementary Figure S2a(ix). Metabolites putatively identified in *X. perforans* that are involved in lysine degradation.

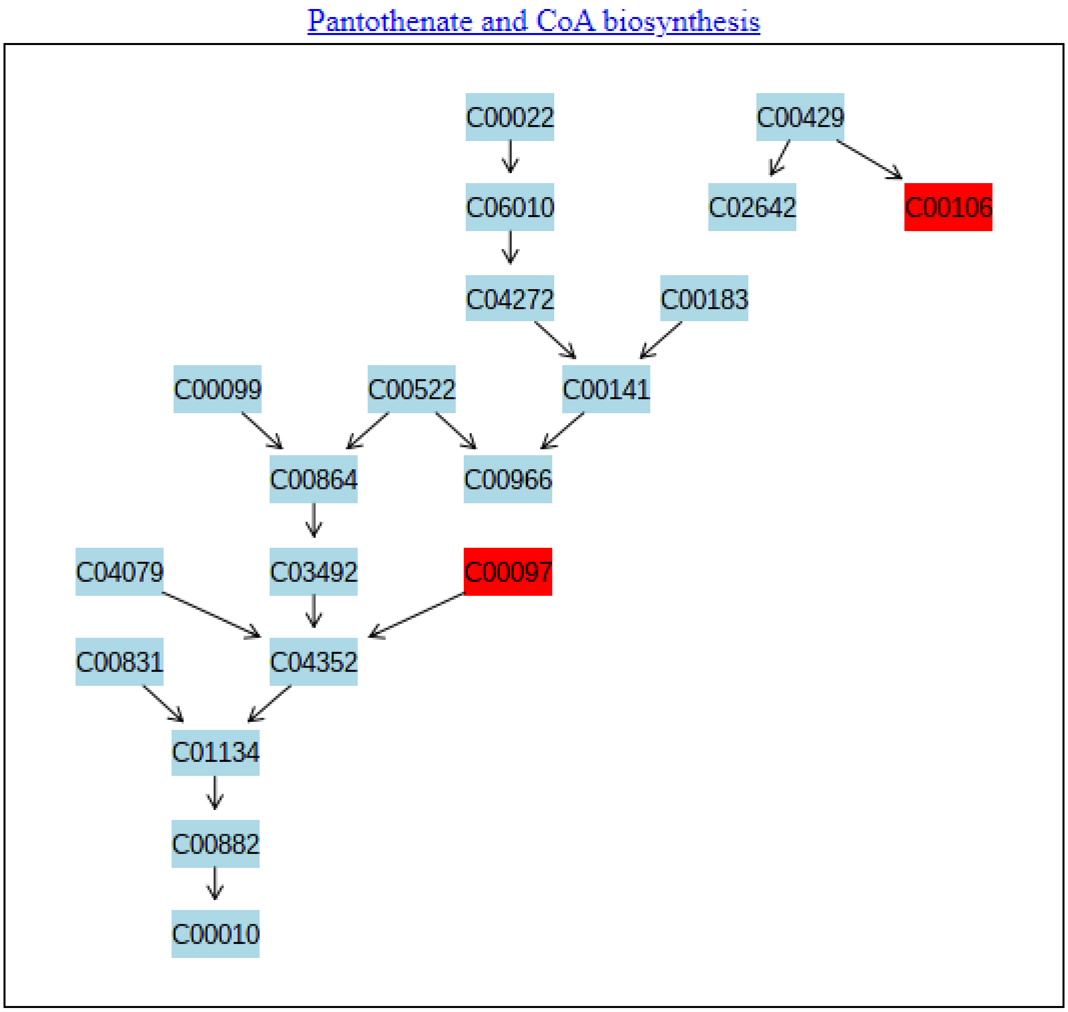

C00106: Uracil, C00097: L-Cysteine

Supplementary Figure S2a (x). Metabolites putatively identified in *X. perforans* that are involved in pantothenate and CoA biosynthesis.

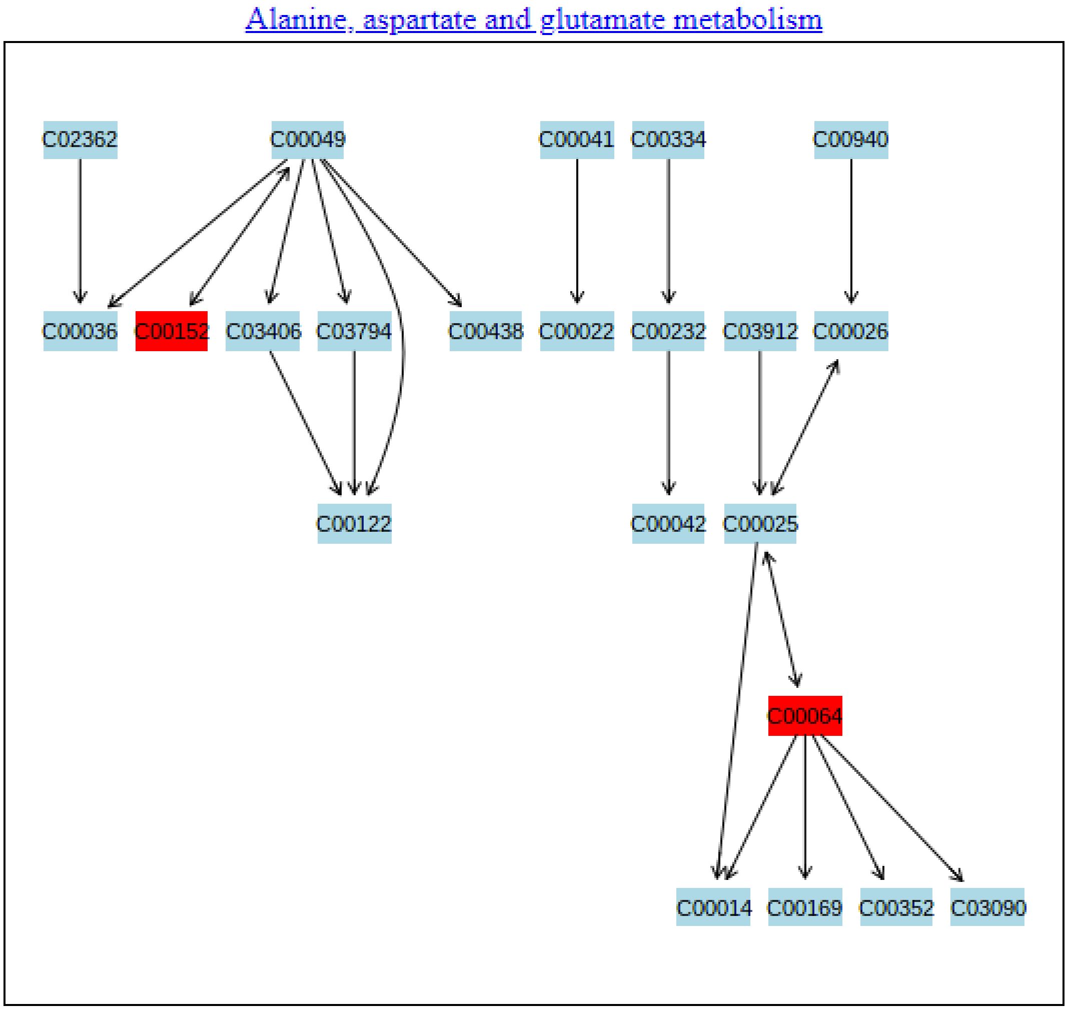

C00152: L-Asparagine, C00064: L-Glutamine

Supplementary Figure S2a(xii). Metabolites putatively identified in *X. perforans* that are involved in alanine, aspartate and glutamate metabolism.

Methane metabolism

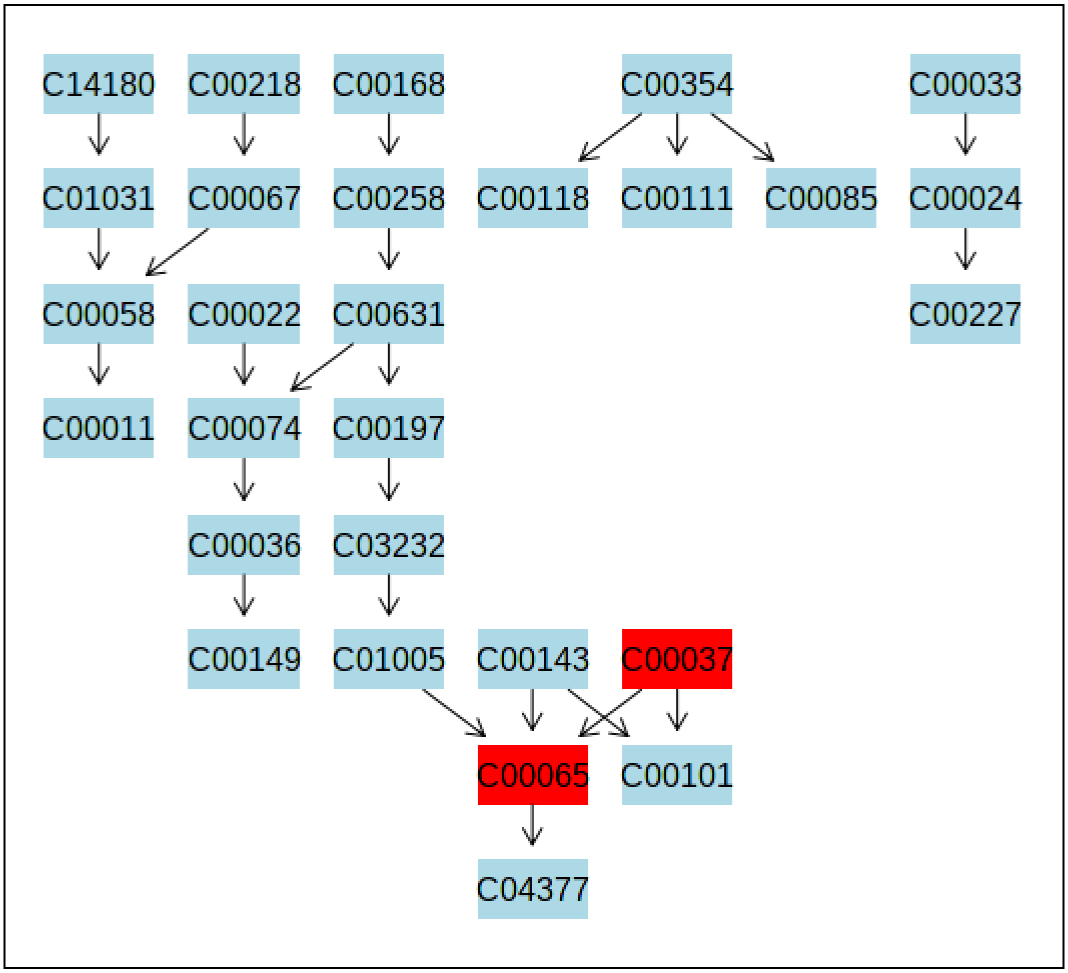

C00027: Glycine, C00065: L-Serine

Supplementary Figure S2a (xii). Metabolites putatively identified in *X. perforans* that are involved in methane metabolism.

NEGATIVE PHASE SIGNIFICANT METABOLITES AND PATHWAYS

Metabolites putatively identified in *X. perforans* that are involved

Metabolites putatively identified in *X. perforans* that are involved

Aminoacyl-tRNA biosynthesis

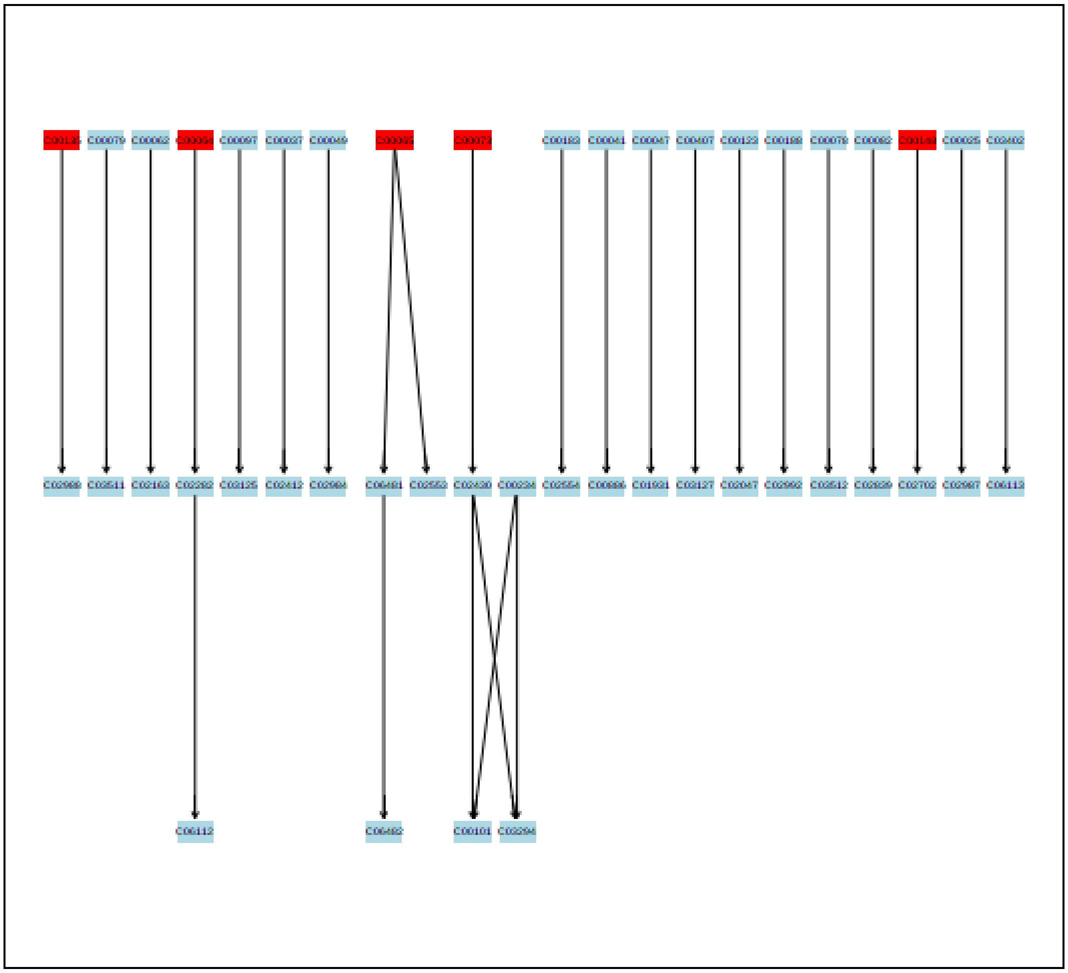

C00125: L-Histamine; C00064: L-Glutamine; C00065: L-Serine; C00072: L-Methionine; C00148: L-Proline

Supplementary Figure S2b(i). Metabolites putatively identified in *X. perforans* that are involved in aminoacyl-tRNA biosynthesis

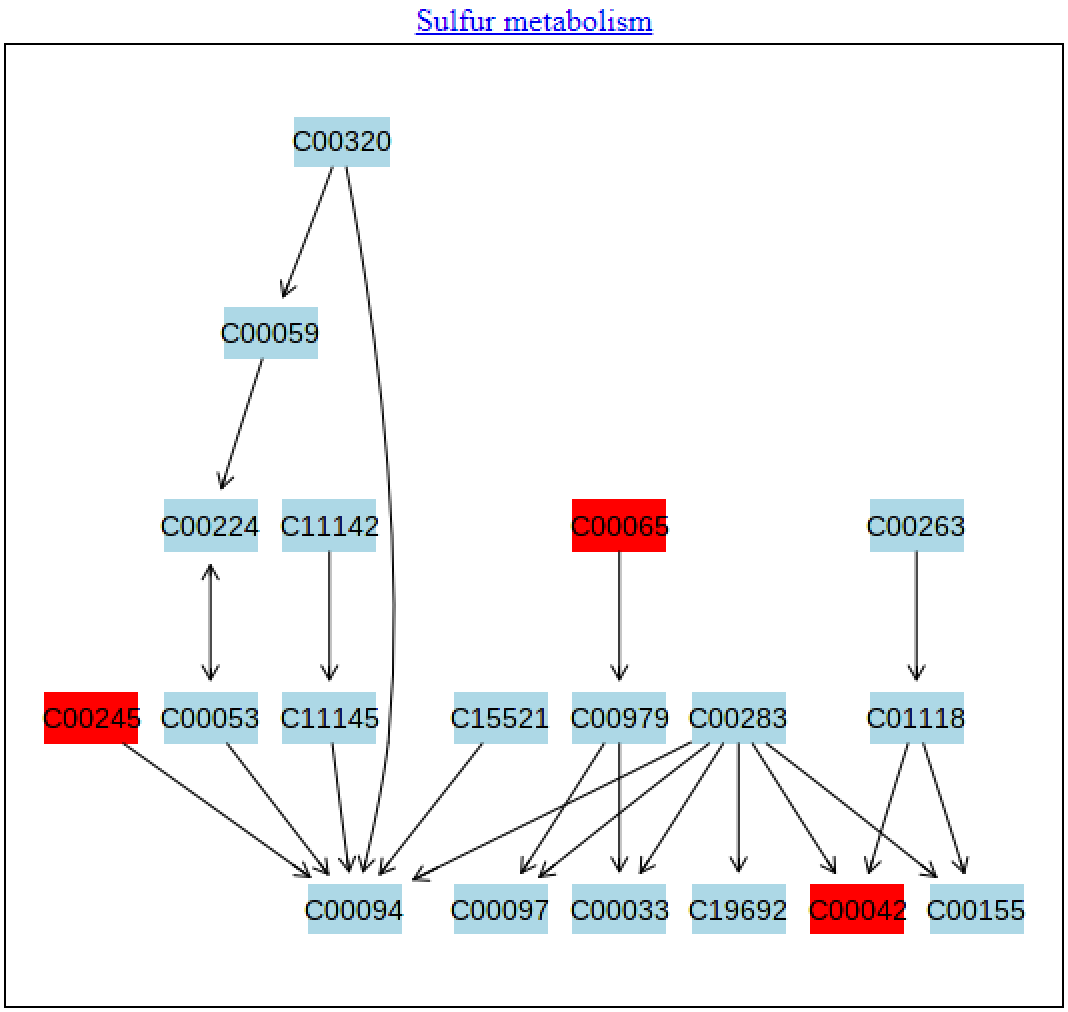

C00065: L-Serine, C00245: Taurine, C00042: Succinate.

Supplementary Figure S2b(ii). Metabolites putatively identified in *X. perforans* that are involved in sulfur metabolism

Pantothenate and CoA biosynthesis

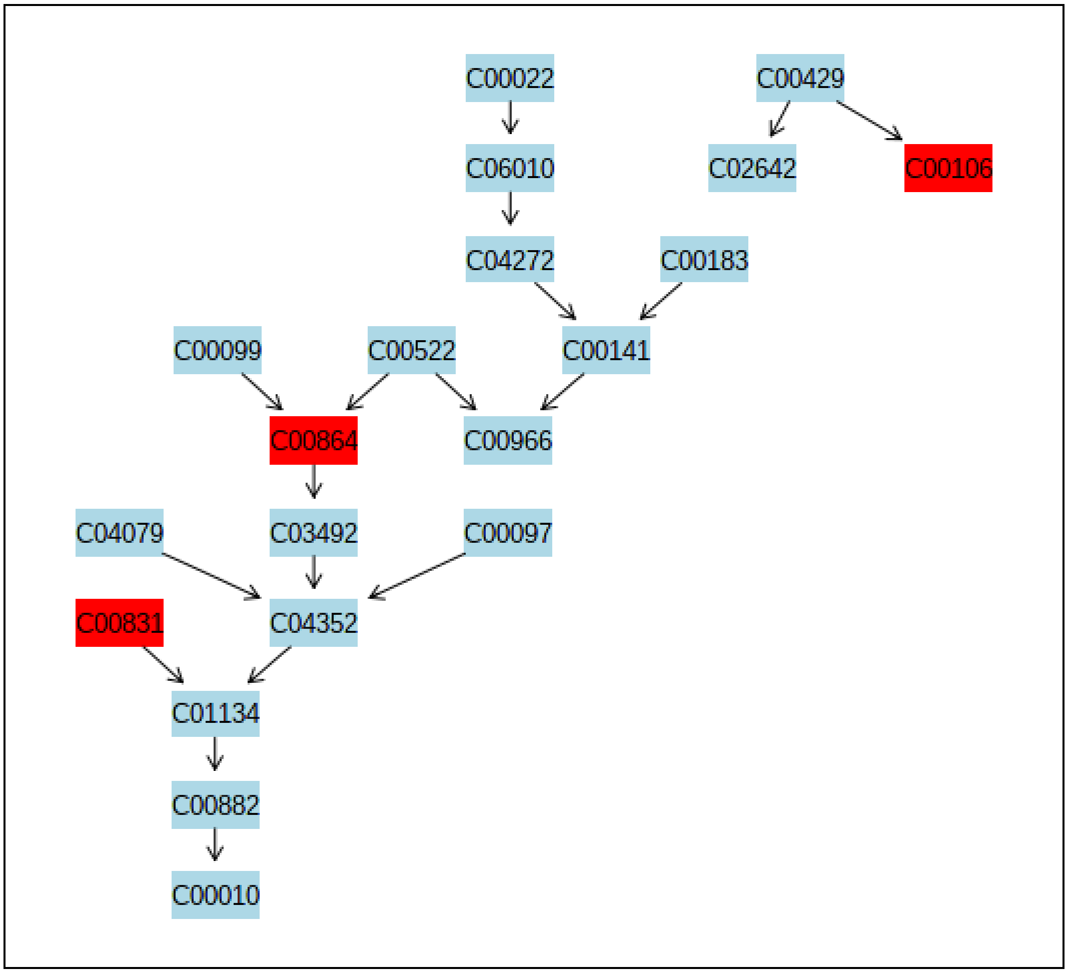

C00106: Uracil, C00864: Pantothenate C00821: Pantetheine

Supplementary Figure S2b(iii). Metabolites putatively identified in *X. perforans* that are involved in pantothenate and CoA biosynthesis

Alanine, aspartate and glutamate metabolism

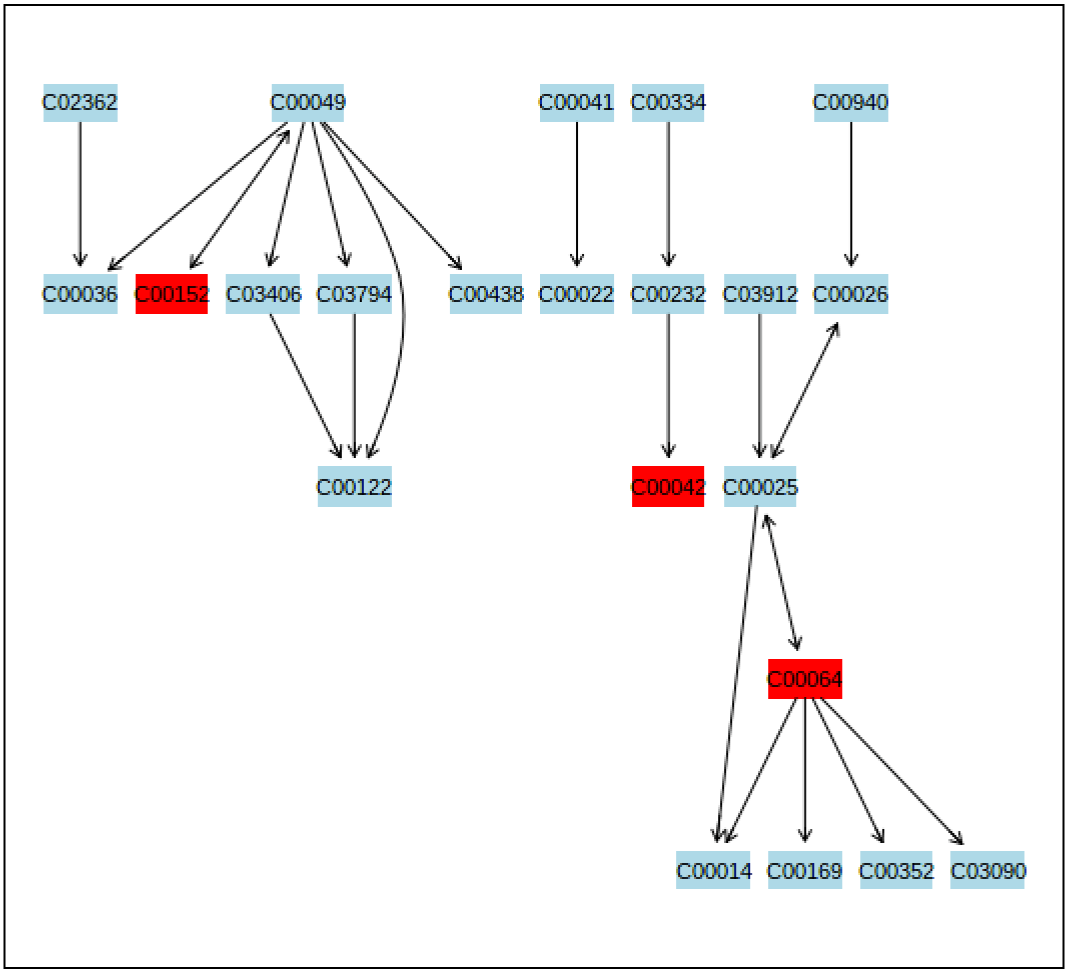

C00152: L-Asparagine, C00042: Succinate C00064: L-Glutamine

Supplementary Figure S2b(iv). Metabolites putatively identified in *X. perforans* that are involved in alanine, aspartate and glutamate metabolism

beta-Alanine metabolism

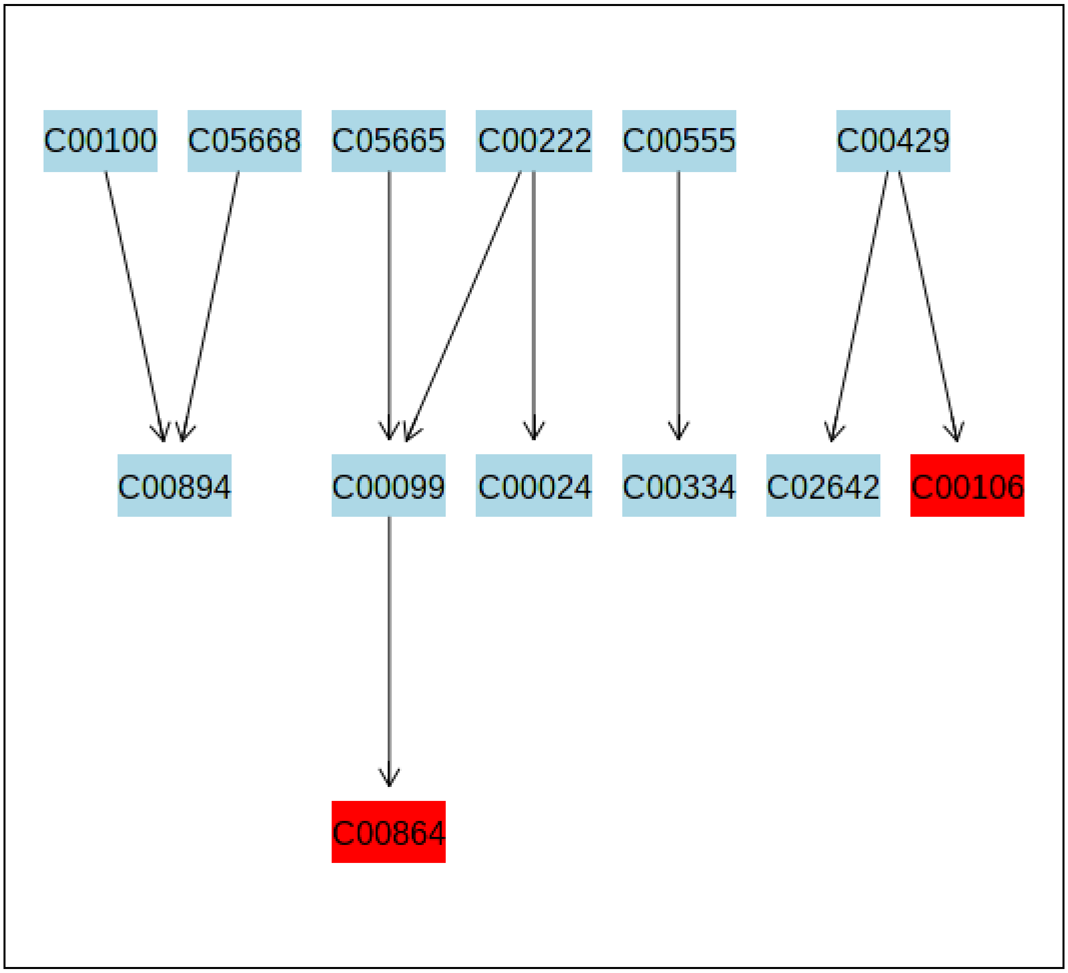

C00106: Uracil, C00864: Pantothenate

Supplementary Figure S2b(v). Metabolites putatively identified in *X. perforans* that are involved in beta-Alanine metabolism

Purine metabolism

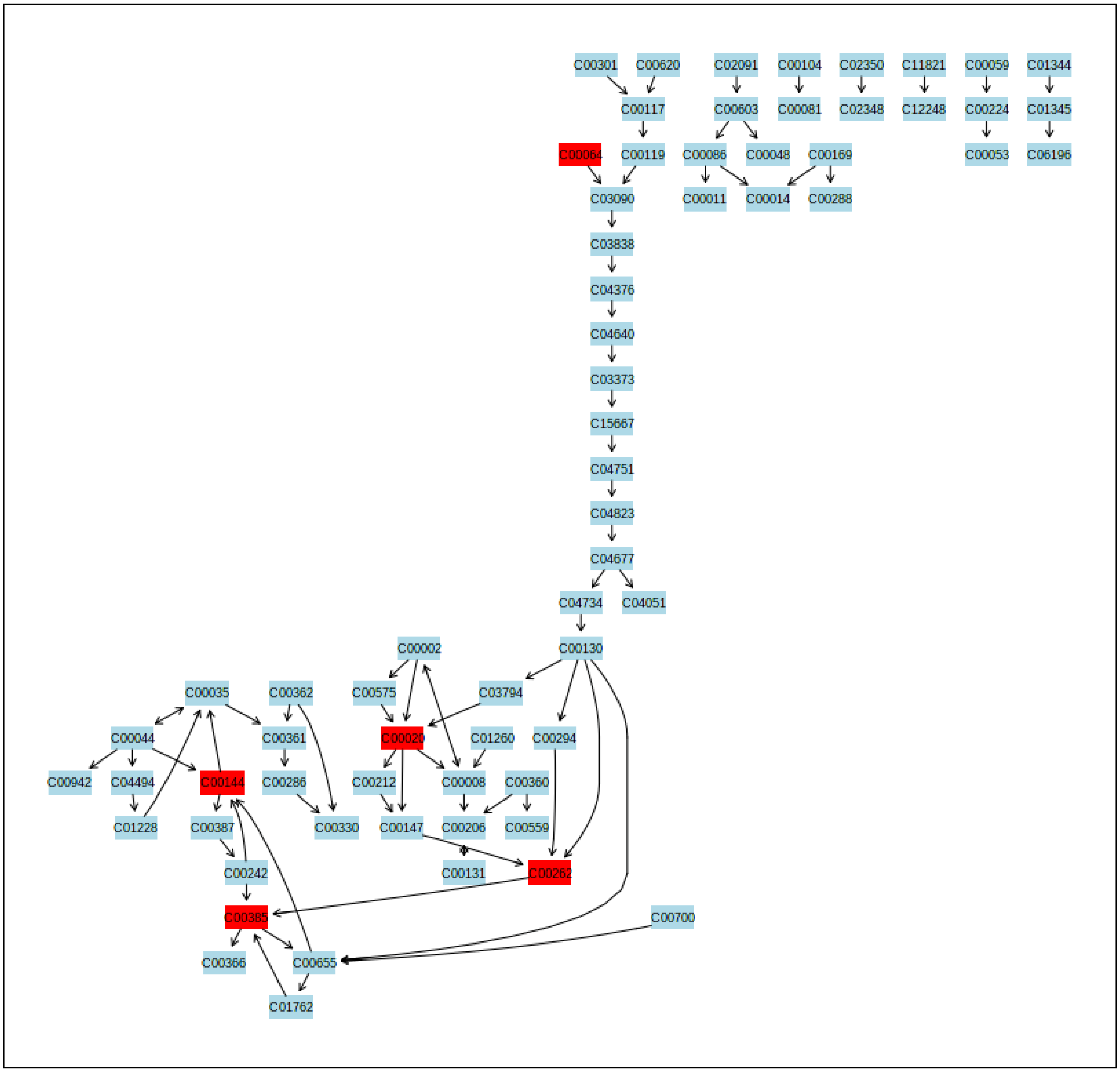

C00064: L-Glutamine, C00020: AMP, C00144: GMP C00262: Hypoxanthine; C00285: Xanthine

Supplementary Figure S2b(vi). Metabolites putatively identified in *X. perforans* that are involved in purine metabolism

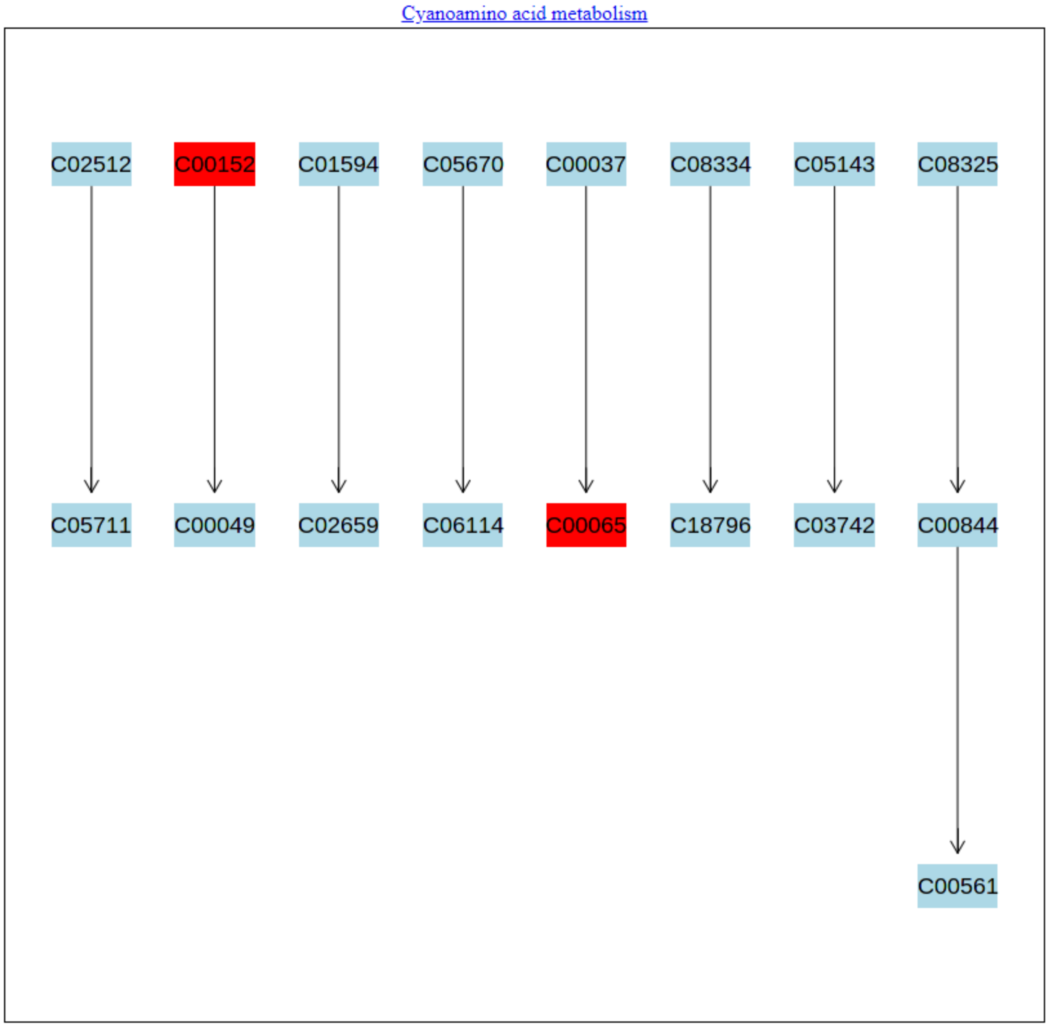

C00152: L-Asparagine, C00065: L-Serine

Supplementary Figure S2b(vii). Metabolites putatively identified in *X. perforans* that are involved in cyanoamino acid metabolism

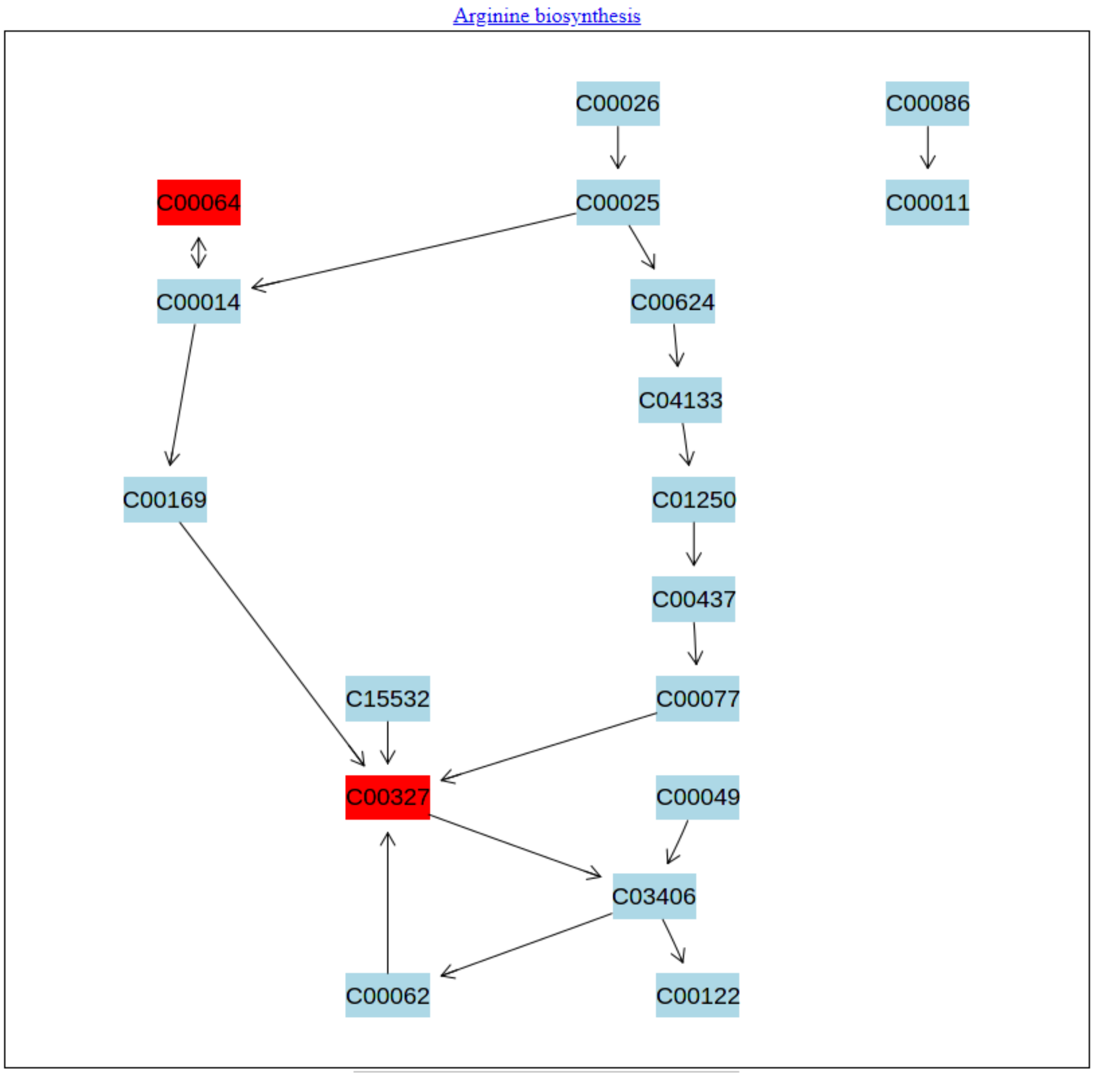

C00064: L-Glutamine, C00227: L-Citrulline

Supplementary Figure S2b(viii). Metabolites putatively identified in *X. perforans* that are involved in arginine biosynthesis

Citrate cycle (TCA cycle)

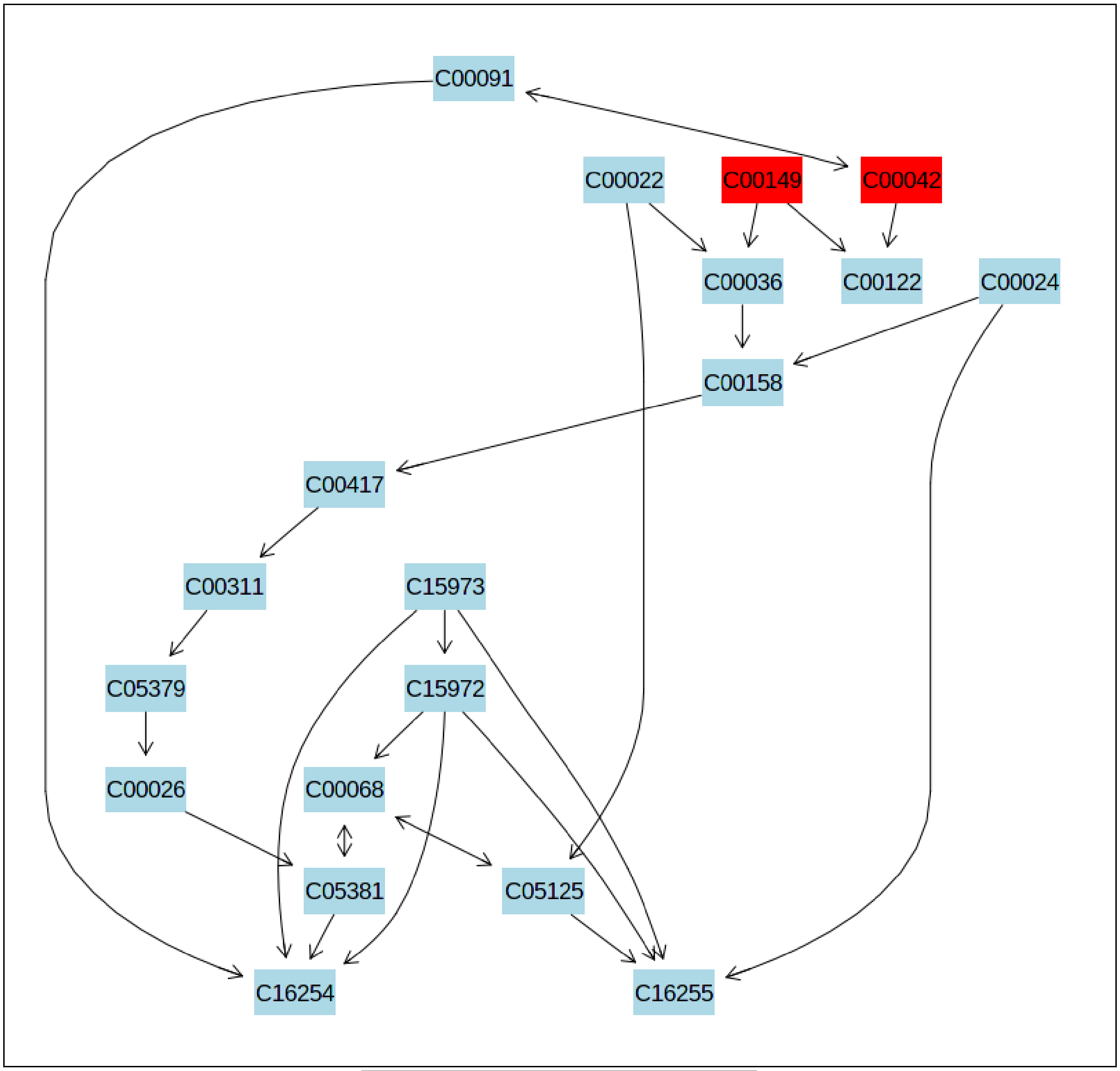

C00149: (S)-Malate, C00042: Succinate

Supplementary Figure S2b(ix). Metabolites putatively identified in *X. perforans* that are involved in TCA cycle.

Histidine metabolism

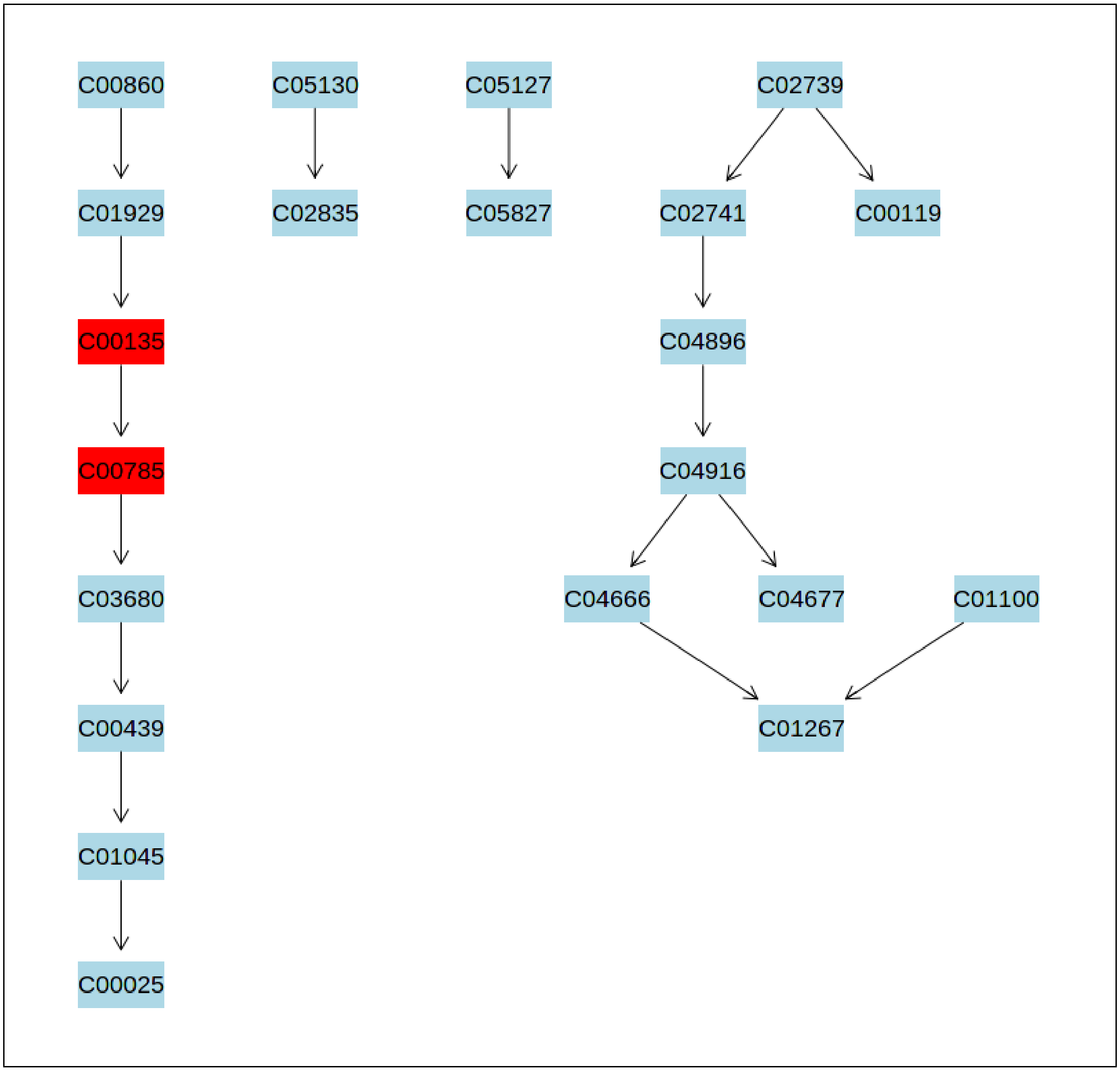

C00125: L-Histidine, C00785: Urocanate

Supplementary Figure S2b(x). Metabolites putatively identified in *X. perforans* that are involved in histidine metabolism.

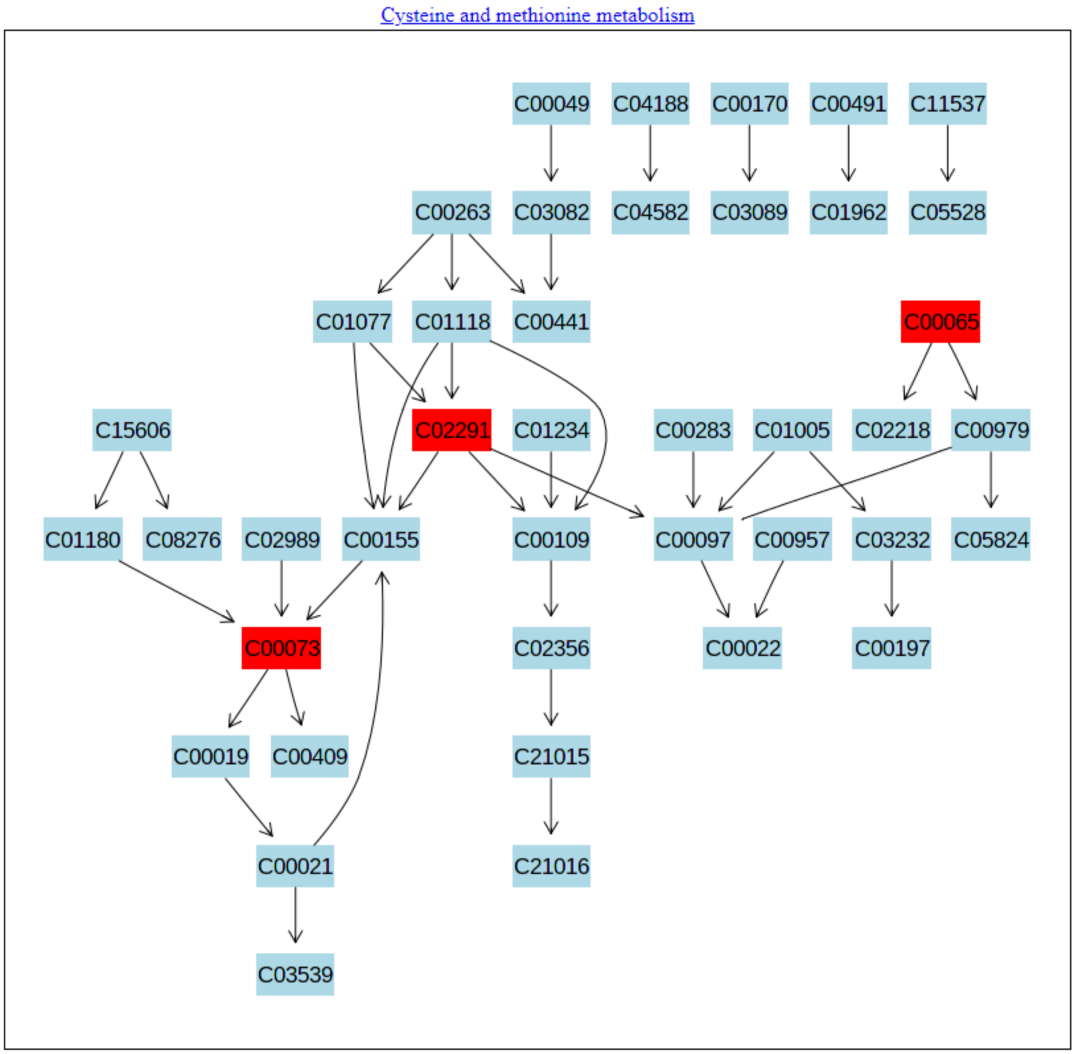

C00065: L-Serine, C02291: L-Cystathionine, C00072: L-Methionine

Supplementary Figure S2b(xi). Metabolites putatively identified in *X. perforans* that are involved in cysteine and methionine metabolism.
