# Supplementary material for: Metabolomics Insights into Chemical Convergence in Xanthomonas perforans and Metabolic Changes Following Treatment with the Small Molecule Carvacrol
Source: Metabolites. 2021 Dec 16;11(12):879. doi: 10.3390/metabo11120879 (PMC8706651; doi:10.3390/metabo11120879)
Supplement: Supplementary file 1 [file metabolites-11-00879-s001.zip › Supplementary File 3-revised.pdf]

Univariate, Multivariate, and Clustering Analyses Using MetaboAnalyst 5.0

The following analyses were carried out in MetaboAnalyst 5.0 (https://www.metaboanalyst.ca/), Chong et al. 2019).

Processing the Raw Data

MetaboAnalyst accepts a variety of data types generated in metabolomic studies, including compound concentration data, binned NMR/MS spectra data, NMR/MS peak list data, as well as MS spectra (NetCDF, mzXML, mzDATA). In our study, we utilized curated peak intensity table for known significant metabolites in both positive and negative ion modes. Features with more than 50% missing values were removed. For all analyses, Pearson r correlation was used.

Normalization of Data

Sample normalization was by median score and data were transformed using the generalized logarithm transformation. The density plots are based on all samples. The boxplots show at most 50 features due to space limit.

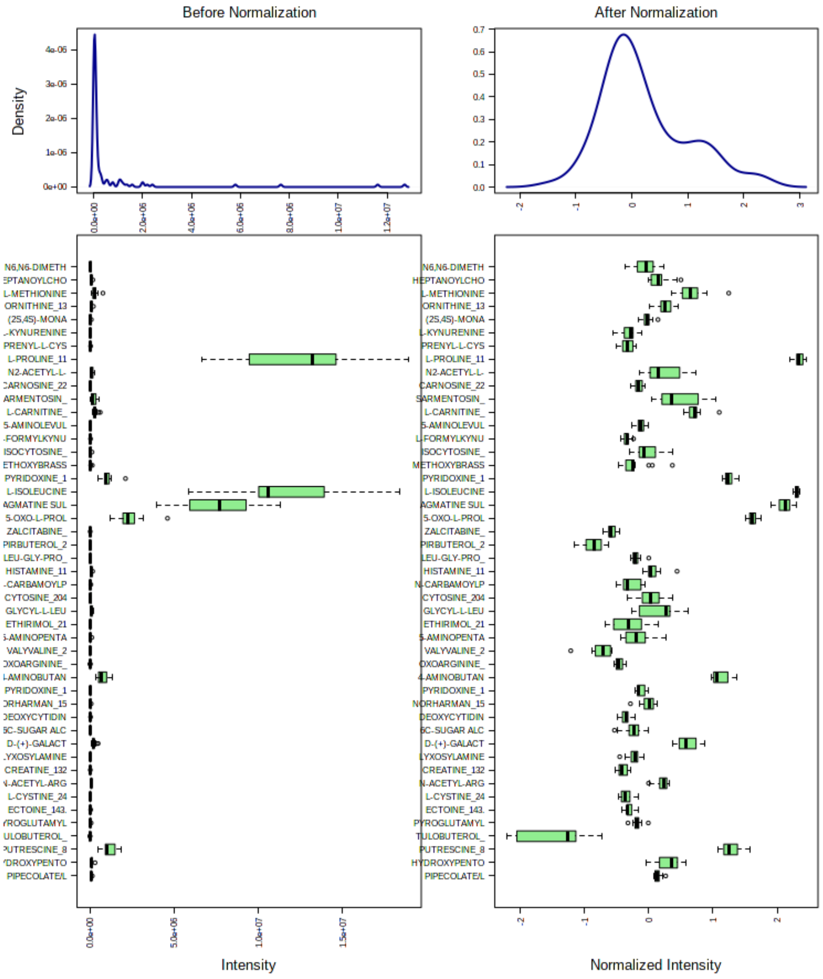

Supplementary Figure S3(i). Data normalization for positive Mode metabolites peak dataset.

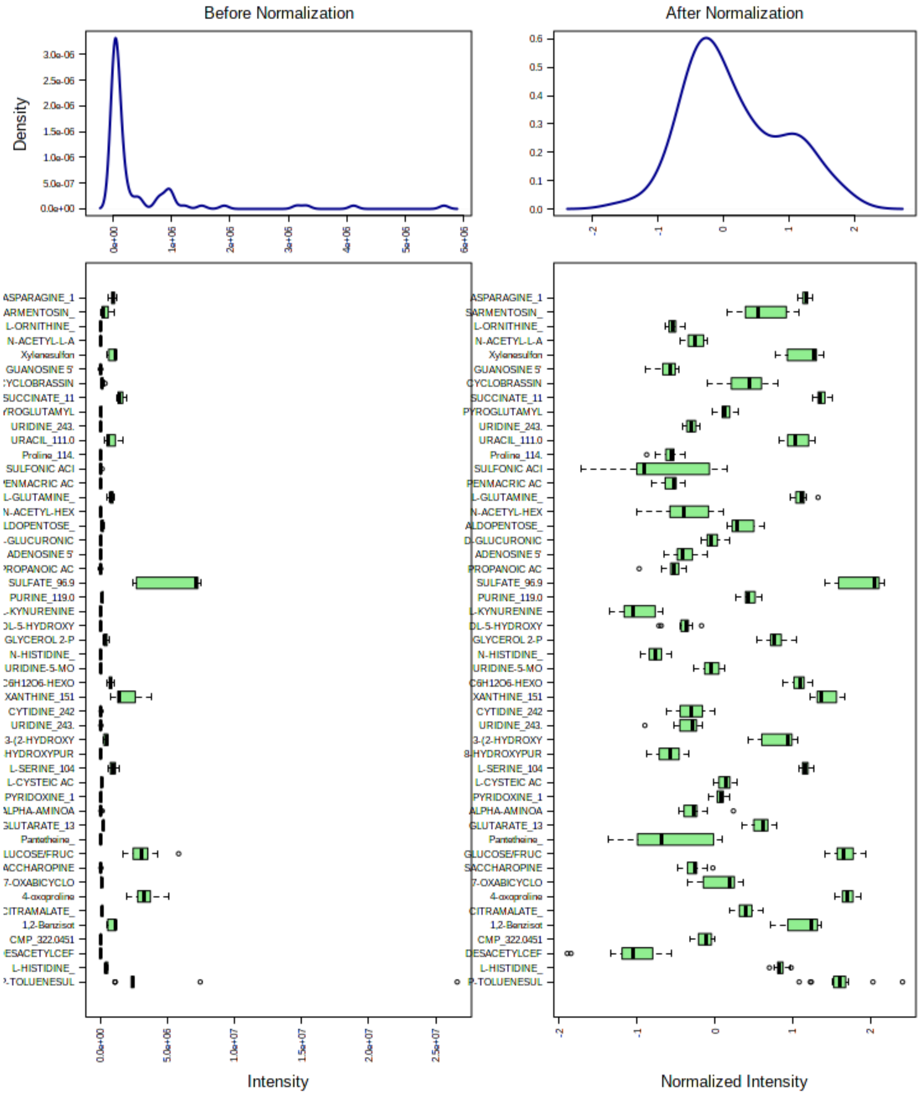

Supplementary Figure S3(ii). Data normalization for negative Mode metabolites peak dataset.

Correlation Heatmaps

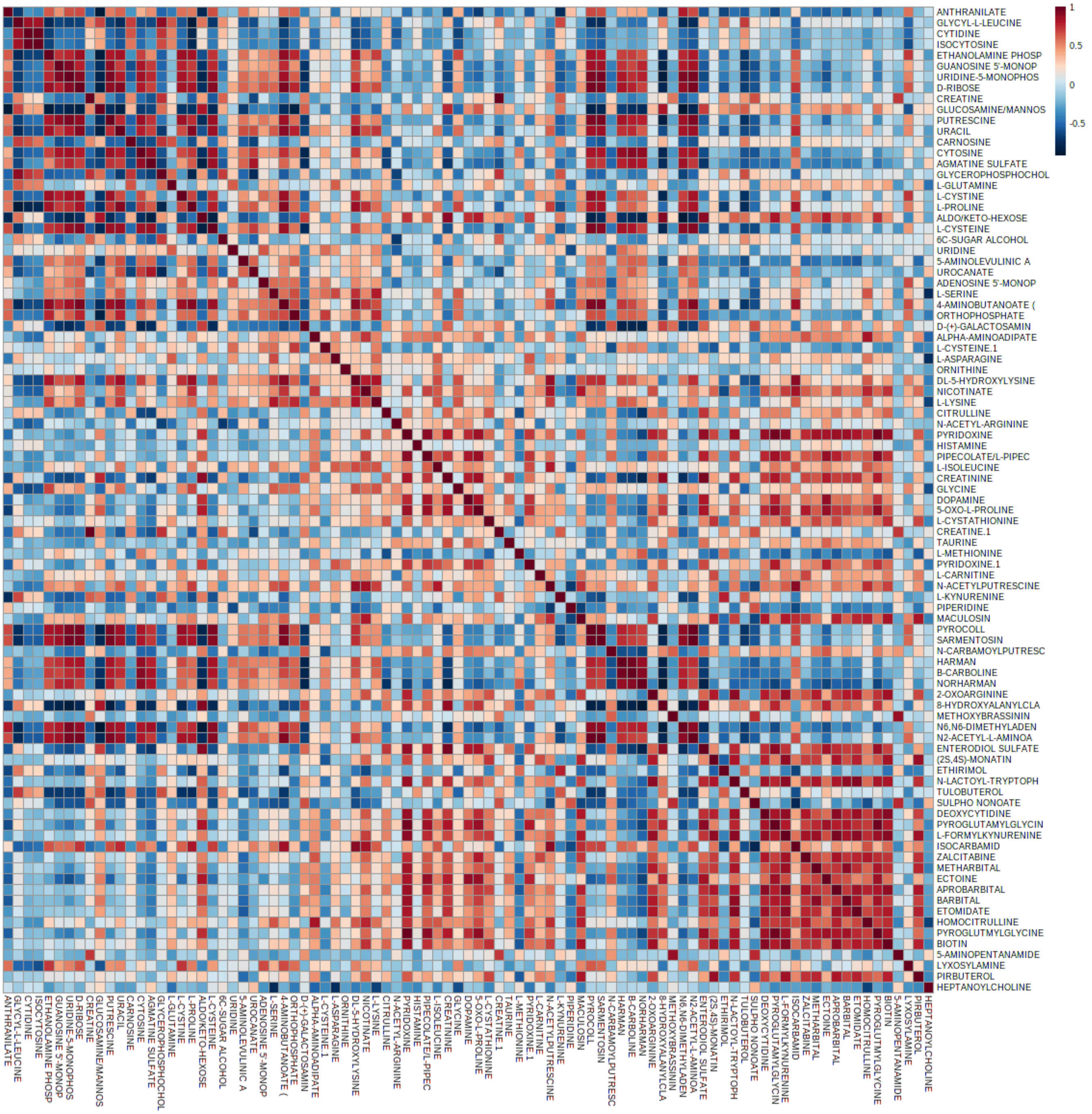

Supplementary Figure S3 (iii). Correlation heatmaps (based on Pearson r) for Metabolites in Positive ion Mode

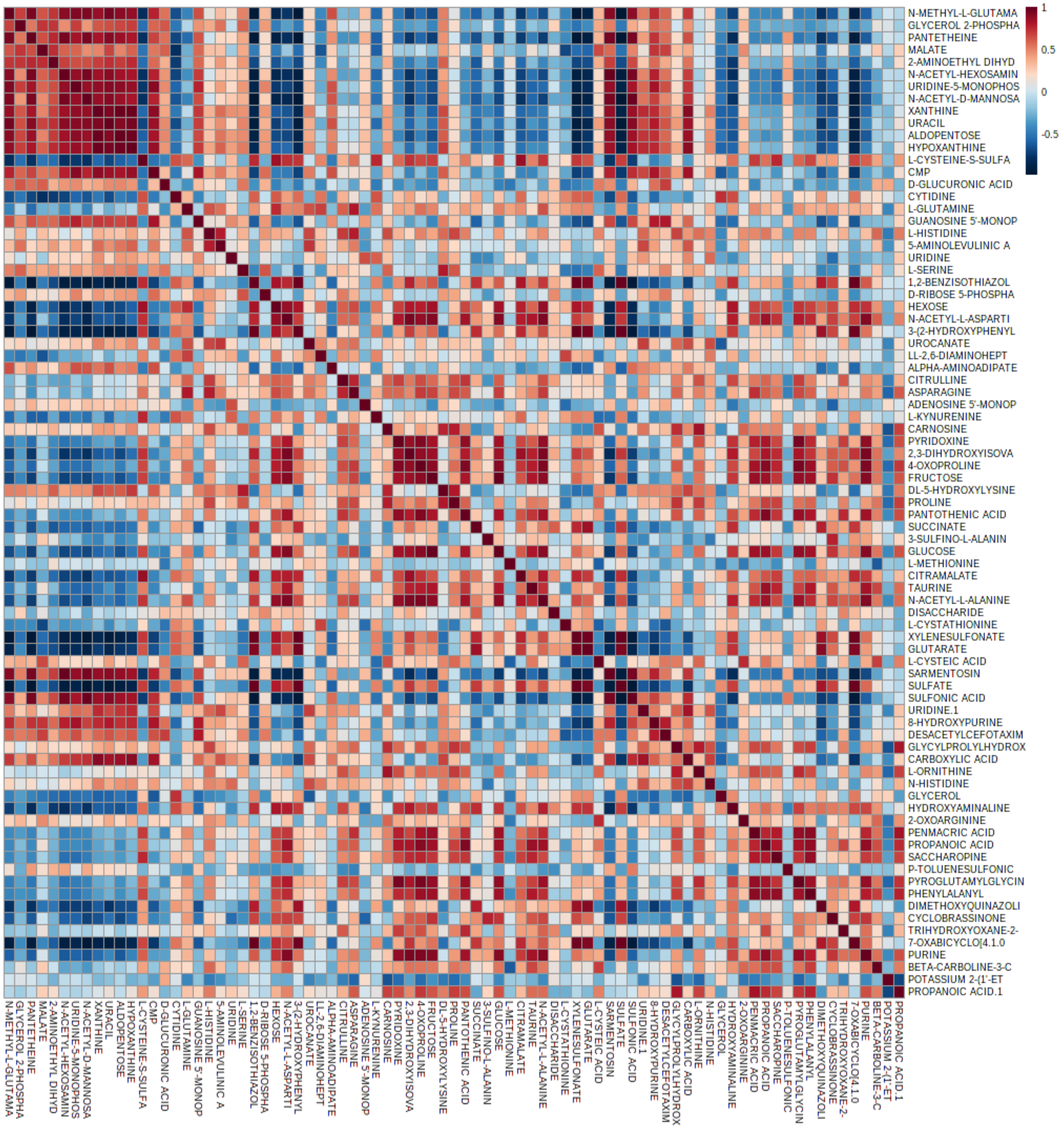

Supplementary Figure S3 (iv). Correlation heatmaps for (based on Pearson r) for Metabolites in Negative ion mode

Principal Component Analyses (PCA)

PCA is an unsupervised method aiming to find the directions that best explain the variance in a dataset (X) without referring to class labels (Y). The PCA analysis is performed using the `prcomp` package. The calculation is based on singular value decomposition.

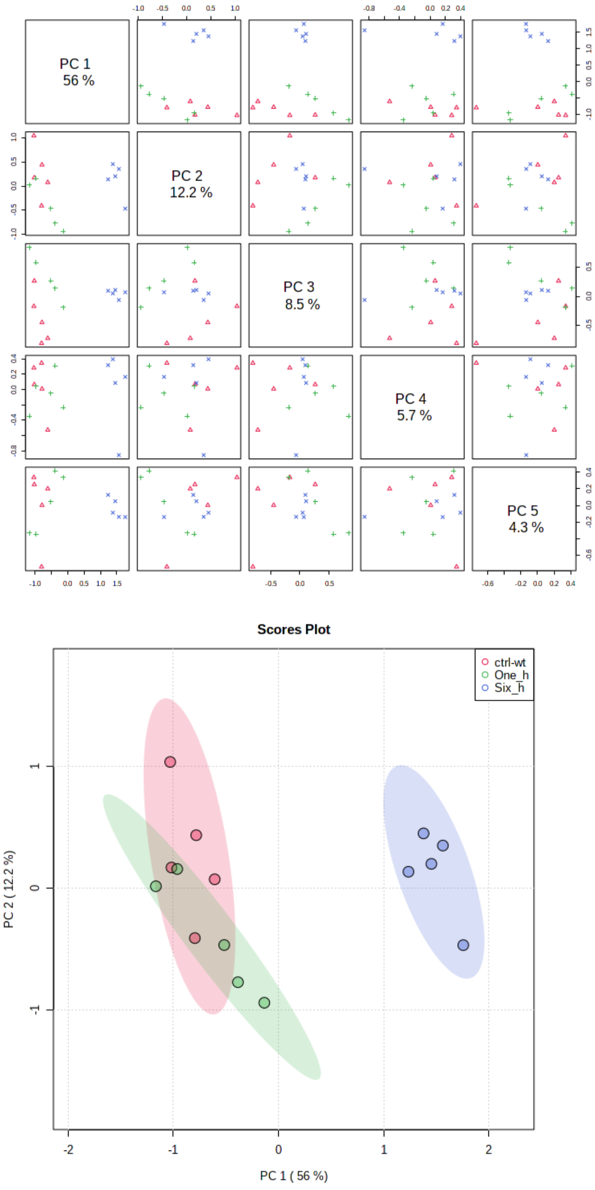

Supplementary Figure S3 (v). Principal Component Analyses (PCA) for Samples in Positive Ion Mode

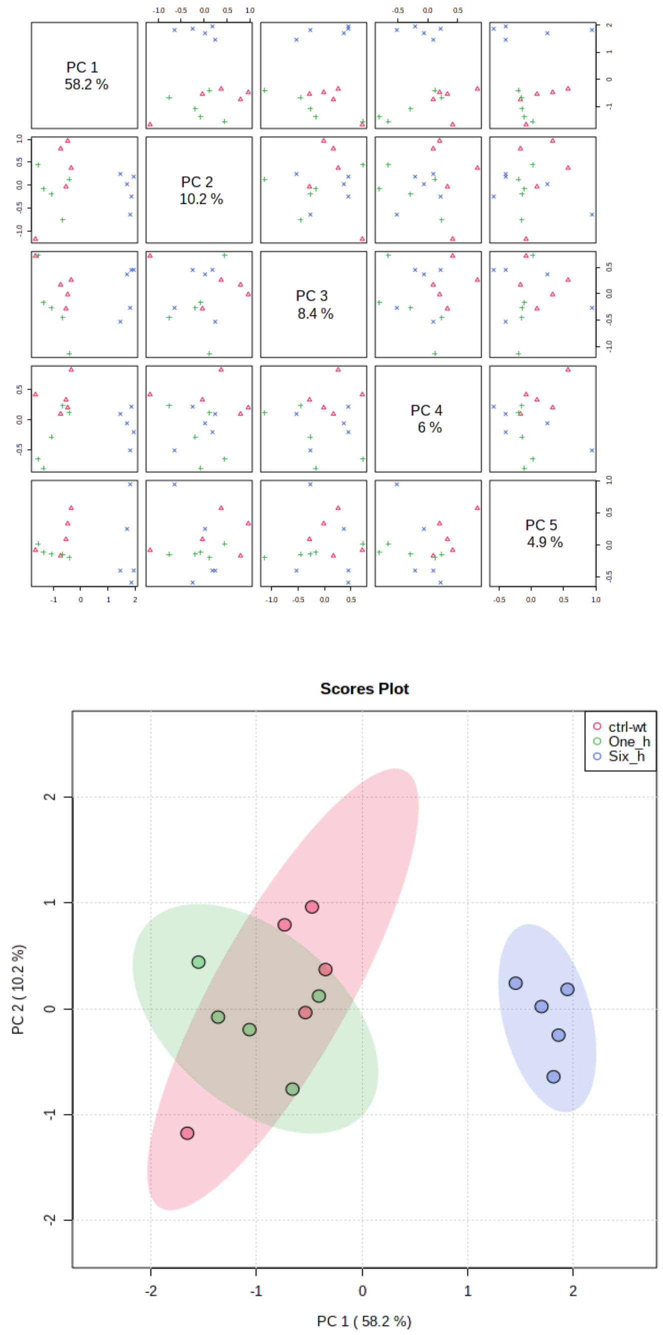

Supplementary Figure S3 (vi). Principal Component Analyses (PCA) for Samples in Negative Ion Mode

Partial Least Square Discriminant Analysis (PLS-DA)

PLS is a supervised method that uses multivariate regression techniques to extract via linear combination of original variables (X) the information that can predict the class membership (Y). The PLS regression is performed using the plsr function provided by R pls package (Wehrens and Mevik, 2007). The

classification and cross-validation are performed using the corresponding wrapper function offered by the caret package (Kuhn, 2008). To assess the significance of class discrimination, a permutation test was performed. In each permutation, a PLS-DA model was built between the data (X) and the permuted class labels (Y) using the optimal number of components determined by cross validation for the model based on the original class assignment. For each Mode, the overview score plots is followed by the 2-D scores plot.

Positive Ion Mode

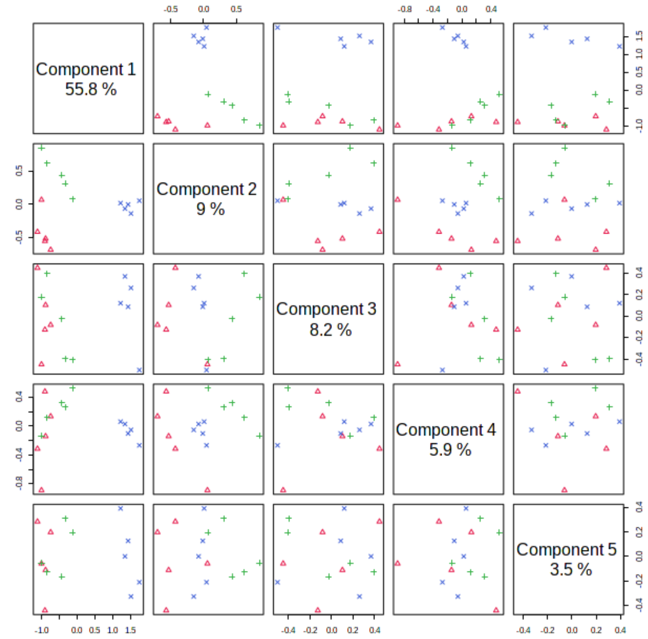

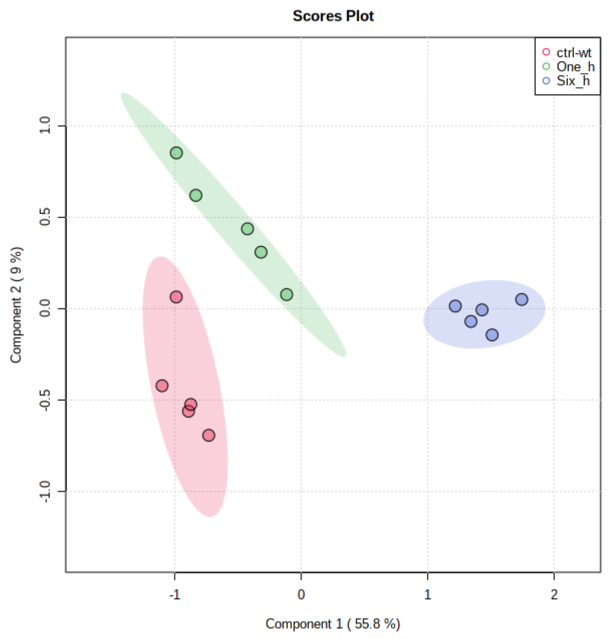

Supplementary Figure S3 (vii). Partial Least Square Discriminant Analysis (PLS-DA) for Samples in Positive Ion Mode

Negative Ion Mode

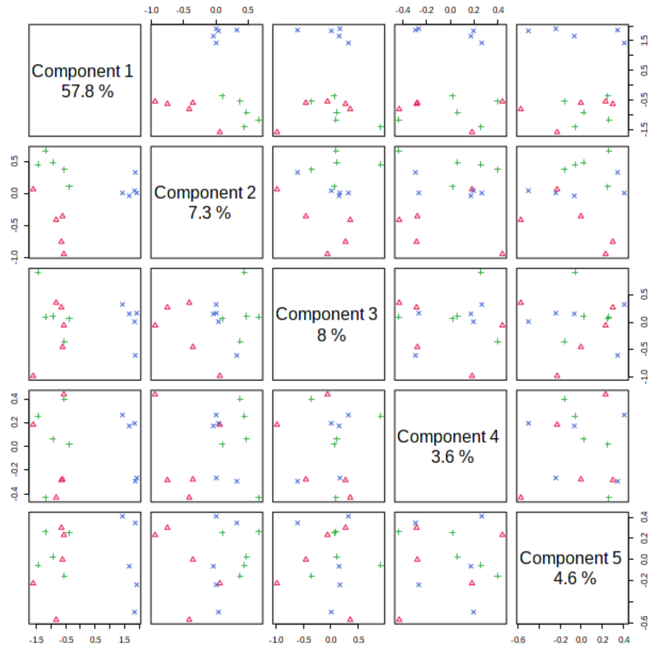

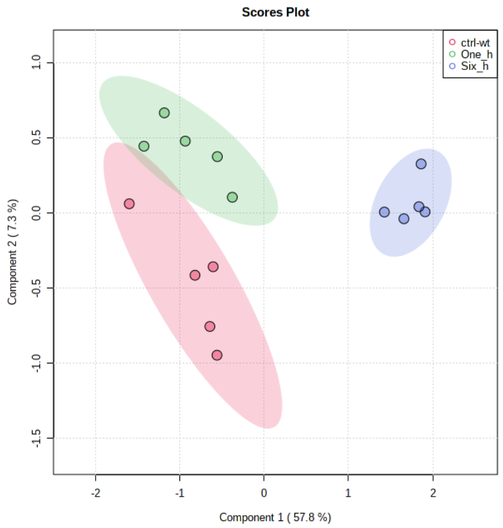

Supplementary Figure S3 (viii). Partial Least Square Discriminant Analysis (PLS-DA) for Samples in negative Ion Mode

Sparse PLS-DA

Positive Ion Mode

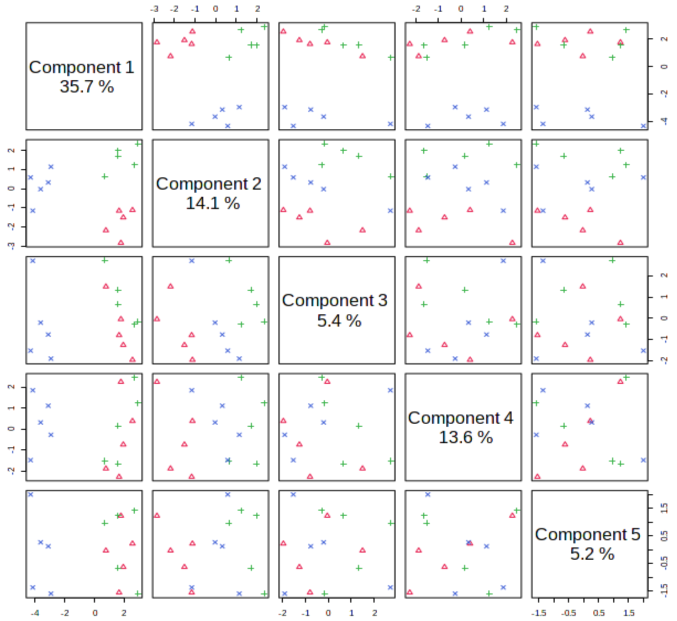

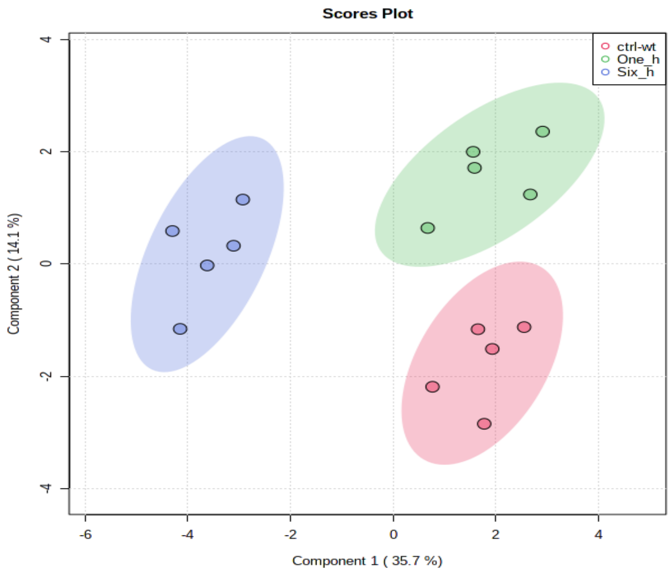

Supplementary Figure S3 (ix). Sparse Partial Least Square Discriminant Analysis (Sparse PLS-DA) for Samples in Positive Ion Mode

Negative Ion Mode

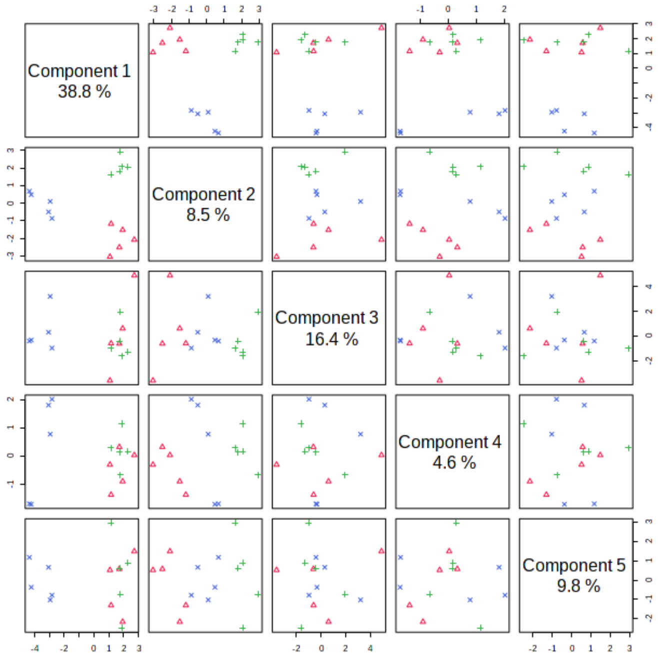

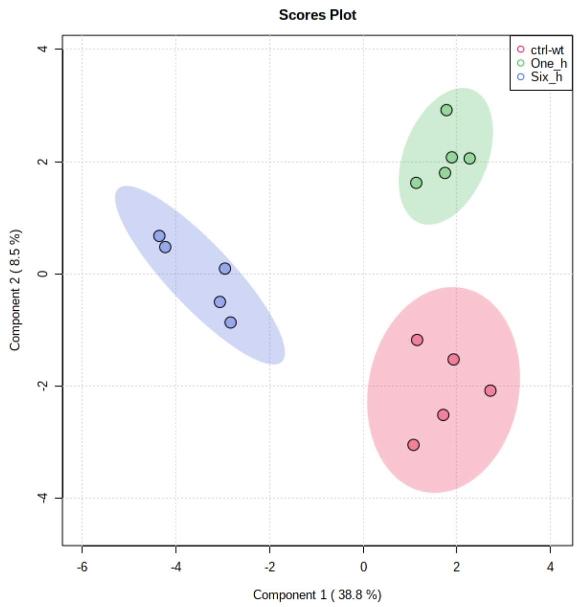

Supplementary Figure S3 (x). Sparse Partial Least Square Discriminant Analysis (Sparse PLS-DA) for Samples in Negative Ion Mode

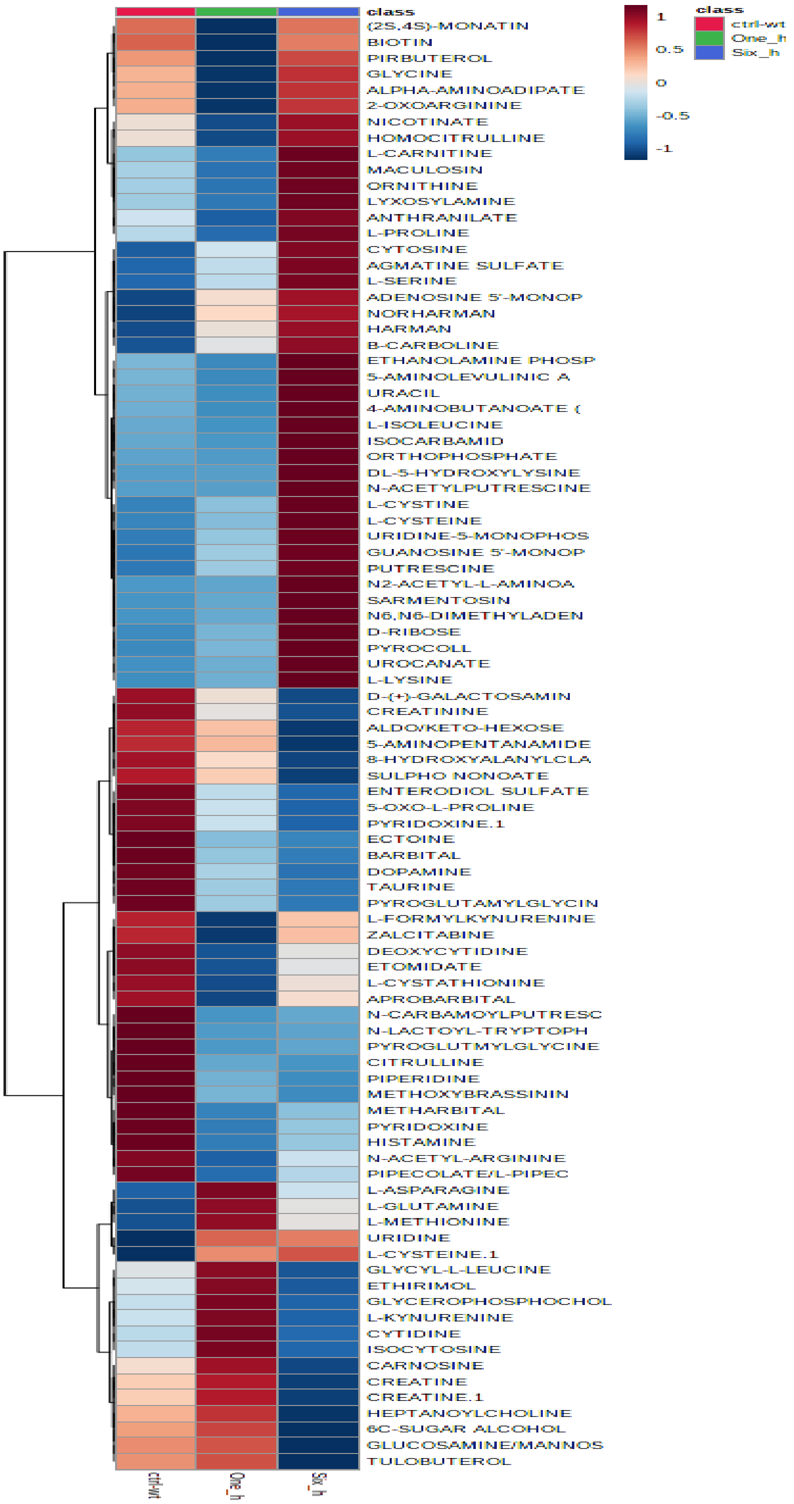

Supplementary Figure S3 (xi). Heatmap Showing variations in effect of carvacrol on *X perforans* utilizing putatively identified metabolites in the positive ion mode. The average variation of the five replicates for each treatment is shown for untreated wild type (ctrl-wt), 1h after treatment with carvacrol (One_h) and 6h after treatment with carvacrol (Six_h).

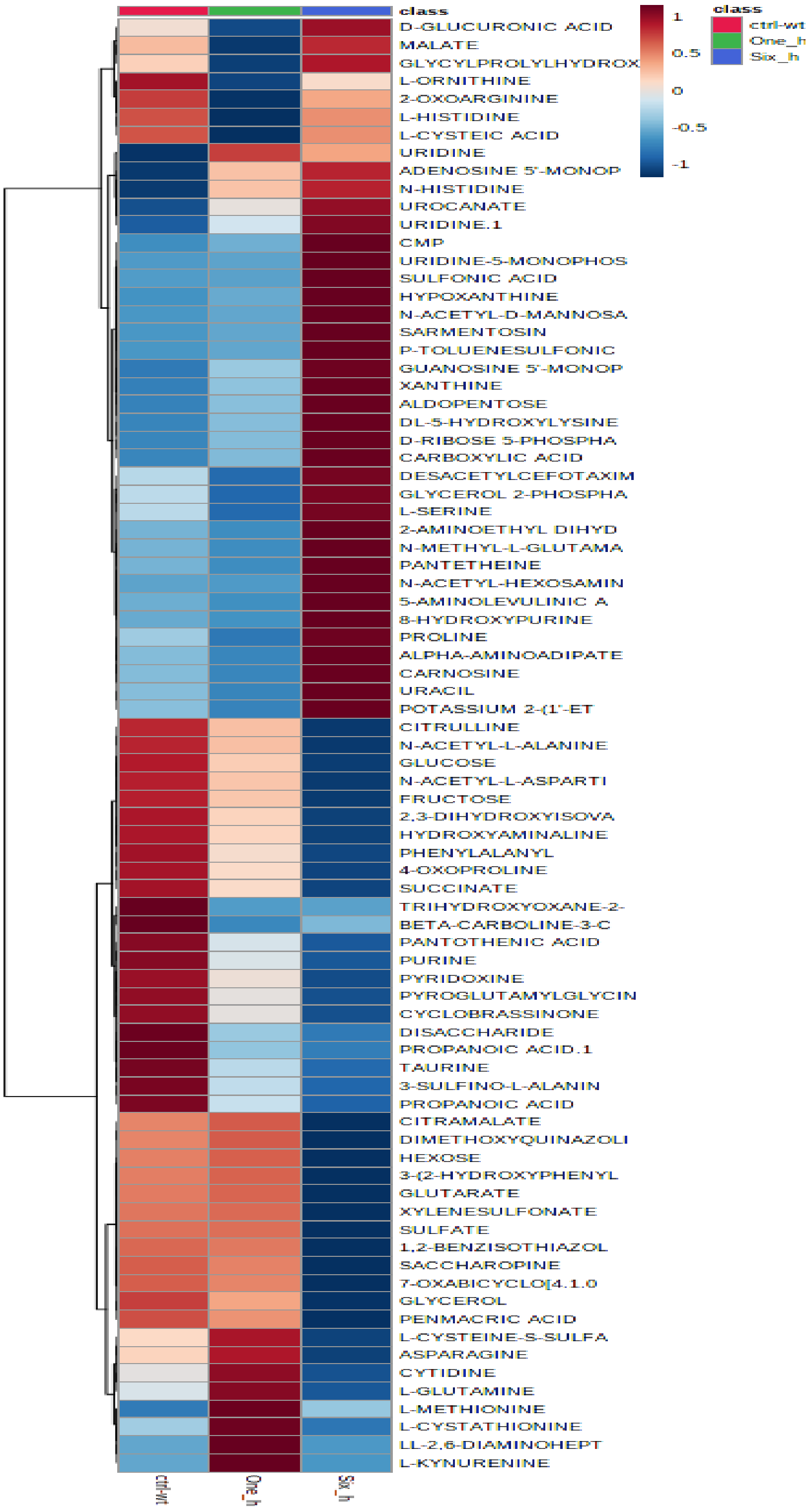

Supplementary Figure S3 (xii). Heatmap Showing variations in effect of carvacrol on *X perforans* utilizing putatively identified metabolites in the negative ion mode. The average variation of the five replicates for each treatment is shown for untreated wild type (ctrl-wt), 1h after treatment with carvacrol (One_h) and 6h after treatment with carvacrol (Six_h).

Supplementary Table S5a. Major patterns of variation of metabolites in Carvacrol treated Xp91-118 in Supplementary Figures S3xi and S3xii.

Group	Pattern of variation	Metabolites
A	Moderate intensity constitutively[1], very low intensity at 1 h and high intensity at 6 h	L-proline, L-serine, L-lysine, 5-aminolevulinic acid, ornithine, maculosin, L-carnitine, N-actylputrescine, alpha-aminoadipate, nicotinate, L-isoleucine, glycine
B	very low intensity constitutively, high intensity at 1 h and 6 h	L-methionine, L-glutamine, uridine
C	low/very low intensity constitutively and at 1 h but very high intensity at 6 h	3-aminobutanoate, uracil, ethanolamine phosphate, orthophosphate, urocanate, anthranilate, DL-5-hydroxylysine, adenosine 5-monophosphate, norharman, harman, beta-carboline, L-cystine, D-ribose, sarmentosin, uridine 5-monophosphate, putrescine, guanosine 5-monophosphate, pyrocoll, cytosine, agmatine sulfate, L-cysteine, Aldopentose, xanthine, N-acetyl hexoseamine, Asparagine, hypoxanthine, CMP, N-acetyl-D_mannoseamine, 5-aminolevulinic acid, Pantetheine, proline, L-serine, carnosine, Alpha-aminoadipate, Malate, D-Glucoronic acid, citrulline, L-Histidine, glycerol-2-phosphate
D	high intensity constitutively and reduced intensity at 1 and 6 h	creatine, pyridoxine, piperidine, N-carbamoylputrescine, dopamine, 5-oxo—L-proline, histamine, taurine, citrulline, pipecolate, glucose/fructose, 3-Sulfino-L-Alanine, N-Acetyl-L-Alanine, N-Acetyl-L-Aspartic acid, 2,3-dihydroxyisovalerate,
E	high intensity constitutively, reduced intensities at 1 h and slightly high intensity at 6 h	2-oxoarginine, L-asparagine, L-cystathionine, N-acetyl-arginine
F	Moderate intensity constitutively, high intensity at 1 h and very low intensity at 6 h	cytidine, isocytosine, L-kynurenine, glycyl-L-leucine, glycerophosphocholine
G	high intensity constitutively, reduced intensity at 1 h and very low intensity at 6 h	creatine, glucosamine, 6C-sugar alcohol

Supplementary Table S5b. Major patterns of variation of metabolites in Carvacrol treated Xp91-118 in Supplementary Figure S3 (xvix) and S3 (xx) continued.

Group	Pattern of variation	Metabolites
H	high intensity constitutively, low intensity at 1 h and very low intensity at 6 h	8-hydroxyalanylclavam, carnosine, aldo/keto-hexose, D-(+)-galactosamin
I	Moderate intensity constitutively, moderate/high intensity at 1h, low intensity at 6h	1,2-benzisothiazolin-3-one, 3-(2-hydroxyphenyl) propanoate, $C_6H_{12}O_6$-hexose/ketose/inositol, Xylenesulfonate, Citramalate, Glutarate, L-Cysteine-S-Sulfate,

[1]Constitutively refers to untreated wild type.

References

Wehrens, R. and Mevik, B. (2007). *pls: Partial Least Squares Regression (PLSR) and Principal Component Regression (PCR)*, 2007, R package version 2.1-0

Kuhn, M (2008). Contributions from Jed Wing and Steve Weston and Andre Williams. *caret: Classification and Regression Training*, 2008, R package version 3.35
